# Clinical forecasting of acute myeloid leukemia using *ex vivo* drug-sensitivity profiling

**DOI:** 10.1016/j.crmeth.2023.100654

**Published:** 2023-12-07

**Authors:** Aram N. Andersen, Andrea M. Brodersen, Pilar Ayuda-Durán, Laure Piechaczyk, Dagim Shiferaw Tadele, Lizet Baken, Julia Fredriksen, Mia Stoksflod, Andrea Lenartova, Yngvar Fløisand, Sigrid S. Skånland, Jorrit M. Enserink

**Affiliations:** 1 Department of Molecular Cell Biology, Institute for Cancer Research, Oslo University Hospital, Montebello, 0379 Oslo, Norway; 2 Centre for Cancer Cell Reprogramming, Institute of Clinical Medicine, Faculty of Medicine, University of Oslo, Blindern, 0318 Oslo, Norway; 3 Section for Biochemistry and Molecular Biology, Faculty of Mathematics and Natural Sciences, University of Oslo, Blindernveien 31, 0371 Oslo, Norway; 4 Department of Haematology, Oslo University Hospital, 0372 Oslo, Norway; 5 Department of Cancer Immunology, Institute for Cancer Research, Oslo University Hospital, Montebello, 0379 Oslo, Norway; 6 K.G. Jebsen Centre for B Cell Malignancies, Institute of Clinical Medicine, University of Oslo, 0372 Oslo, Norway

## Abstract

Current treatment selection for acute myeloid leukemia (AML) patients depends on risk stratification based on cytogenetic and genomic markers. However, the forecasting accuracy of treatment response remains modest, with most patients receiving intensive chemotherapy. Recently, *ex vivo* drug screening has gained traction in personalized treatment selection and as a tool for mapping patient groups based on relevant cancer dependencies. Here, we systematically evaluated the use of drug sensitivity profiling for predicting patient survival and clinical response to chemotherapy in a cohort of AML patients. We compared computational methodologies for scoring drug efficacy and characterized tools to counter noise and batch-related confounders pervasive in high-throughput drug testing. We show that *ex vivo* drug sensitivity profiling is a robust and versatile approach to patient prognostics that comprehensively maps functional signatures of treatment response and disease progression. In conclusion, *ex vivo* drug profiling can assess risk for individual AML patients and may guide clinical decision-making.

## Highlights

• Systematic evaluation of computational methods to analyze *ex vivo* drug-profiling data
• Identification of a drug fingerprint that forecasts chemotherapy outcome in patients
• Drug profiling predicts treatment outcome at least as well as current AML biomarkers

## Motivation

*Ex vivo* drug-sensitivity screening may be used to assess treatment options and predict treatment response. However, the effectiveness of this approach relies on the preservation of clinical cancer characteristics in *ex vivo* cell systems, and systematic data on accurate representation of drug-response profiles are lacking. Here, we used forecasting of clinical outcomes as a standard for exploring optimal scoring of drug sensitivities and management of noise and technical confounders. We show that *ex vivo* drug-sensitivity profiles robustly predict AML patient survival and provide valuable insights about treatment response and disease progression.

## Graphical abstract

**Figure:**
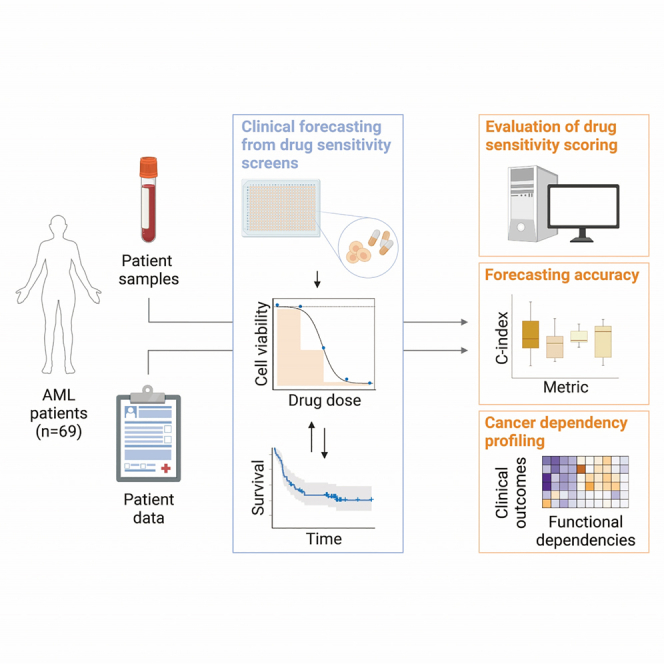

## Teaser

Predicting the response to chemotherapy for treatment of AML is a major challenge. Mutation analysis and gene panel diagnostics are routinely used to predict treatment outcome, but the predictive power of these methods is limited. Here, Andersen et al. demonstrate that *ex vivo* drug profiling can assess risk of individual AML patients and may guide clinical decision-making.

## Introduction

Acute myeloid leukemia (AML) is a heterogeneous cancer where the clonal expansion of myeloid progenitor cells (blasts) in the bone marrow and peripheral blood interfere with healthy hematopoiesis, resulting in immunodeficiency, thrombocytopenia, and anemia.^1^ The current 5-year survival rate of patients over 60 years of age is estimated to be 10%–15%.^2^ Most treatment-eligible individuals receive standard induction chemotherapy, which consists of a 3-day treatment with either 60 mg/m^2^ daunorubicin or 10–12 mg/m^2^ idarubicin and 100–200 mg/m^2^ cytarabine (Ara-C) intravenously for 7 days.^3^ Survival rates of older patients have not substantially improved over the past decades, underscoring the need for better clinical assessment and more accurate prognostic approaches.

Current risk stratification methods such as the European LeukemiaNet (ELN) guidelines for AML stratification are mainly based on parameters such as molecular pathology and genomic alterations.^4^ However, a substantial number of patients remain difficult to stratify, such as cytogenetically normal AML, and approximately 50% of patients are stratified as intermediate-risk patients for whom selection of the appropriate treatment regimen remains a major challenge.^4^^,^^5^^,^^6^ Various non-genomic methods have been developed for identification of clinically and biologically relevant molecular subtypes for risk stratification, such as flow and mass cytometry, transcriptomics, and proteomics,^7^^,^^8^^,^^9^^,^^10^^,^^11^^,^^12^ although clinical implementation of these methods has generally been slow.

Recently, *ex vivo* drug sensitivity profiling has been used as a precision medicine approach to identify potential compounds that may be repurposed for the treatment of various types of cancer, including AML.^13^^,^^14^^,^^15^^,^^16^^,^^17^^,^^18^^,^^19^^,^^20^ Here, leukemic cells derived from bone marrow aspirates or peripheral blood are incubated in the presence of various drugs at different concentrations. Drug responses are then quantified, and overall drug efficacy is scored from a dose-response relation. This approach allows for screening of a high number of compounds in just 3 days and has identified potential alternative treatment avenues for AML.^13^^,^^14^^,^^15^^,^^16^^,^^17^^,^^18^^,^^19^

The validity of drug sensitivity profiling requires that clinically relevant characteristics are conserved in *ex vivo* analyses; i.e., drug responses should reflect cancer dependencies, and drug sensitivities should be measurable reliably despite potential technical confounders and noise. Several quality control practices have been developed to ensure the fidelity of high-throughput drug testing.^21^^,^^22^^,^^23^^,^^24^ A common procedure is to use model curve fitting to de-noise dose-response data and summarize the drug sensitivities with the half-maximal effective concentration (EC_50_) or the area under the curve (AUC).^25^^,^^26^ Development of dose-range standardized function integrals based on the Hill equation, such as the drug sensitivity score (DSS), have improved the reproducibility of drug sensitivity profiles across independent studies.^27^^,^^28^^,^^29^ However, curve fitting of incomplete or non-sigmoidal drug responses remains a challenge for large-scale drug testing, and single output metrics from Hill models tend to yield incomplete information about the biological variability of a given drug response.^25^^,^^30^ Therefore, streamlined analysis pipelines typically offer multiple alternative dose-response evaluation and error-reporting tools.^23^^,^^31^

While *ex vivo* drug screening of cancer cells using clinically relevant chemotherapeutics has been shown to predict treatment outcome, a systematic evaluation of the methodology and clinical use of drug sensitivity profiling in more expansive drug sets has not been done.^32^^,^^33^^,^^34^^,^^35^ In this study, we evaluated the clinical information available in *ex vivo* drug sensitivity profiles within a cohort of 69 AML patients by using several statistical techniques and machine learning routines. Regularized regression was used to evaluate the clinical forecasting potential and risk-interpretability from *ex vivo* drug sensitivity profiles. We show that drug sensitivity profiling can be used as a tool for clinical forecasting and decision-making.

## Results

### Study approach, quality control, and comparison of metrics

**Figure 1:**
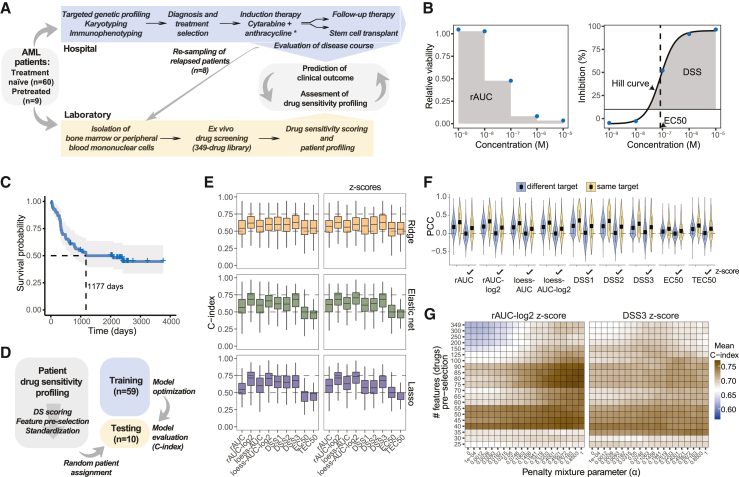
Survival prediction from *ex vivo* drug sensitivity profiles (A) Study overview and workflow. (B) Drug sensitivity metric computation. The gray areas indicate how the rAUC and DSS are calculated. (C) Survival curve of the study cohort. Ticks indicate censoring. (D) Machine learning routine for testing survival prediction from different drug sensitivity metrics and data processing operations. (E) C-index results (200 tests) for Cox models trained on different drug sensitivity metrics (left) or drug sensitivity *Z* scores (right). (F) Pearson correlation coefficients of drug sensitivities between drug pairs having the same or different target. (G) Mean test C-index results (50 tests) for Cox models trained on rAUC-log_2_ or DSS3 *Z* scores with various penalty mixture parameters (*α*) and feature pre-selection thresholds based on rAUC standard deviations.

To evaluate the clinical utility of *ex vivo* drug sensitivity profiling in AML, we systematically performed drug screens on bone marrow or peripheral blood samples from patients that were obtained at the time of diagnosis (Figure 1A and Table S1). For high-throughput curve fitting and multiparametric scoring of drug sensitivities, we used the Breeze pipeline, which performs Hill curve fitting on inhibitory responses to compute several drug sensitivity metrics, such as the EC_50_, toxicity EC_50_ (TEC_50_), and DSS1, DSS2, and DSS3.^23^^,^^27^ Here, DSS1 is the relative integral for Hill curves that exceed 10% inhibition, DSS2 adjusts DSS1 to normalize for strongly toxic responses at high concentrations, and DSS3 adjusts DSS2 to give weight to responses with high dose sensitivity (Figure 1B, see STAR Methods). We also used Breeze to compute the AUC under a locally estimated scatterplot smoothing (LOESS) curve fit (loess-AUC), as a more flexible model-free alternative to the Hill equation. In addition to these metrics, we calculated a raw AUC based on a stepwise normalized rectangular area under the relative viability dose response along a logarithmic concentration scale (rAUC; Figure 1B). Since the distributions of relative viabilities are positively skewed, we also performed a negative log_2_-transformation of the rAUCs, resulting in a weighted-average log_2_ fold change in cell viability (rAUC-log_2_). This step was equivalently performed on the loess-AUCs.

To obtain a measure of the quality of the drug screens we computed plate-wise Z′-factors and analyzed drug sensitivity profile correlations between patients to detect potential outliers (Figures S1A–S1D). Although some patient-specific plates had a suboptimal Z′-factor (Figure S1A), all negative plate controls (DMSO) were at least two standard deviations above the positive plate controls (BzCl, Figure S1B), and we did not observe any consistent association between plate control noise and patient profile correlations (Figure S1C). However, we did find substantial differences in profile correlations for different metrics, with DSS1-3, rAUC-log_2_, and loess-AUC-log_2_ yielding the best inter-patient correlations (median Pearson correlation coefficient [PCC] over 75%) and TEC_50_ and EC_50_ giving the worst (Figures S1C and S1D). Furthermore, intra-patient profile correlations between treatment-naive and relapsed samples were higher than inter-patient correlations (Figure S1D). Comparison of the different metrics revealed that low-confidence Hill curve fits reported by Breeze tend to be associated with low-sensitivity dose-responses (EC_50_ > 10^−7^ μM) (Figure S1E), as well as with responses yielding lower correspondence between different AUC metrics. Despite this, the overall correlation between the different metrics was high, indicating that inferences made from the drug screen dataset were sufficiently robust for these analysis methods.

### Assessing the predictivity of drug sensitivity metrics

Median survival in the patient cohort was 1,177 days (Figure 1C). To assess *ex vivo* drug sensitivity profiling in predicting patient survival, we compared several regularized Cox models trained on the different drug sensitivity metrics (Figure 1D). Predictive accuracy was assessed using a C-index over multiple randomized assignments of patient test data (Figure 1D). We also tested different regularization types, as Ridge and Elastic net regression tend to perform better than Lasso when there are grouped correlations between features (which is expected for drugs with similar modes of action).^36^ AUC-based metrics outperformed EC_50_ and TEC_50_ in predicting patient survival regardless of regularization technique (Figure 1E). Interestingly, the loess-AUC, which had a strong linear correlation with the rAUC (Figure S1E), improved the survival prediction for Elastic net and Lasso models, suggesting that de-noising dose-response data using a LOESS curve fit has a beneficial effect (Figure 1E, left). This was also the case for the DSS metrics for all three model types, with DSS3 outperforming DSS1 and DSS2 and resulting in lower overfitting (Figures 1E and S1H). Strikingly, log-transforming the AUCs resulted in a substantial improvement in prediction accuracy, performing at least as good as DSS3 (Figure 1E, left), indicating that distributional skewness in relative viabilities has a negative impact on appropriate drug sensitivity scoring.

To counter potential batch effects, we standardized the drug sensitivity distribution for each patient (Figure S1F). This technique transforms the drug sensitivity metrics to a respective *Z* score, assuming that the major differences in patient distributions mainly reflect technical influences and that only changes in the relative magnitude of specific drug sensitivities are relevant. This operation further improved the prediction accuracy for the AUC-based metrics, with rAUC-log_2_ and loess-AUC-log_2_ yielding the best performances with respective median C-index scores of 71% and 74% using Lasso (Figure 1E, right). In contrast, patient standardization of the DSS metrics had little effect on the predictivity and caused a slight reduction in performance for models trained with the Lasso penalty (Figure 1E). To better understand these effects, we measured the inter-drug profile correlations, which revealed library-wide multicollinearity that was removed when standardizing the metrics but maintained to a certain degree for drug pairs targeting similar proteins (Figure 1F). Here, DSS1 and DSS2 showed a slightly stronger correlation than DSS3 and other AUC metrics.

Scaling the drug distributions (Figure S1F) to equalize the penalty of drugs with differences in drug sensitivity variance caused a reduction in prediction accuracy using most metrics except for EC_50_ and TEC_50_, where it caused an improvement (Figure S1I). This suggests that drugs generating weak and noisy responses have a negative impact on the model performances. Given the large number of drugs relative to the number of patients, we therefore tested the effect of pre-selecting features ranked by their standard deviations (to remove weak drug responses) as well as a greater range of Elastic net penalties (Figure S1G). These operations, in particular feature pre-selection, improved the survival predictions further, with rAUC-log_2_ yielding C-index averages over 77% for several Elastic net models (Figures 1G and S1J). Altogether, these results show that there is high prognostic value in patients’ *ex vivo* drug sensitivity profiles.

### Versatility of clinical forecasting and integration with AML biomarkers

**Figure 2:**
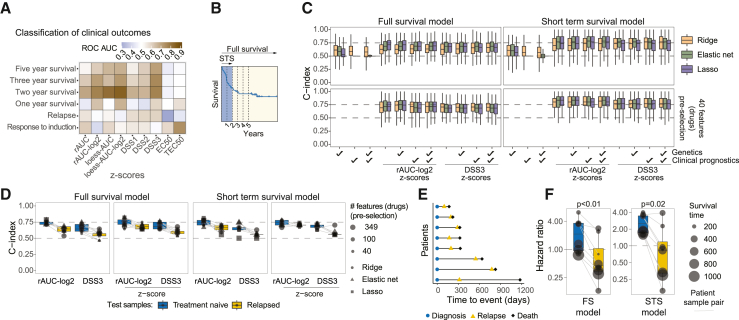
Versatility of drug sensitivity profiling for clinical outcome predictions (A) Average ROC-AUC for classification of various binarized clinical outcomes using different drug sensitivity metrics. The values are averaged over all the ROC-AUC scores from models with different penalties and pre-selection thresholds in Figure S2A. (B) The first 2 years after diagnosis characterized by a 40% drop in survival was defined as an initial phase for short-term survival (STS) modeling using Cox regression. The STS models were compared with the full survival (FS) models that were trained on data from the entire study interval. (C) Test C-index results (200 tests) for Cox models trained on different dataset compositions based on clinical feature sets and rAUC-log_2_ or DSS3 *Z* scores. The lower panels represent pre-selection of 40 features based on rAUC standard deviations, and the right panels represent prediction results from short-term survival modeling. (D) Test C-index on samples from treatment-naive and relapsed patients, for different models trained on rAUC-log_2_ or DSS3, or their corresponding *Z* scores. The two right panels represent prediction results from short-term survival modeling. (E) Overview of the time of relapse in relation to the time of death for the eight patients in the relapse cohort. (F) Predicted hazard ratio (relative to population median) on samples from treatment-naive and relapsed patients for Elastic net models trained on rAUC-log_2_ using 100 pre-selected features. Right panel represents short-term survival modeling. p values were computed using a paired Wilcoxon test.

To evaluate the prognostic versatility and robustness of *ex vivo* drug profiling, we also tested the forecasting potential of other clinical outcomes, including survival status after different periods. We found that the accuracy of AUC-based metrics at predicting the initial response to induction therapy (as measured by the presence of persistent leukemia in the patients), long-term survival, or probability of relapse was limited (Figures 2A and S2A). In contrast, the survival rates during the first 2 years after diagnosis, which is characterized by a sharp reduction in survival (Figure 2B), were predicted by AUC-based metrics with very high accuracy (Figures 2A and S2A). Furthermore, while rAUC-log_2_ and loess-AUC-log_2_ excelled over DSS metrics at short-term survival prediction, DSS3 robustly performed better than all other metrics in predicting long-term survival (Figures 2A and S2A). These results show that drug sensitivity profiles predict well the initial treatment phase but also have potential for long-term survival forecasting.

We next compared the effectiveness of *ex vivo* drug sensitivity profiling in predicting survival to the predictiveness of other AML patient data, such as genetic features and/or prognostic clinical features that included ELN risk stratifications, age, and sex. We also performed the analysis on 2-year truncated short-term survival data to evaluate the performance on the initial treatment response phase.

As expected, due to coverage sparsity, the Ridge models performed better on the genetic and clinical feature sets (Figure 2C). The genetic feature set provided a prediction with a median C-index of 59% overall and 60% short term, which is close to what has been reported previously using much larger cohorts.^37^ Combining the different clinical feature sets did not improve predictivity, most likely due to redundancy in information and low sample size (Figures 2C, S2B, and S2C). Interestingly, *ex vivo* drug screening performed at least as well as or better than the genetic or clinical feature sets at predicting treatment outcome (Figure 2C). Combining genetic or clinical feature sets with the entire drug dataset did not substantially improve predictivity, and combining genetic features with a reduced drug dataset using feature pre-selection only resulted in a slightly improved but non-significant combination effect (Figure 2C, left lower panel). Furthermore, the feature-reduced rAUC-log_2_ dataset showed prediction accuracy of 83% for two-year survival (Figure 2C, right lower panel), which did not improve further when combined with the genetic or clinical feature sets. There were also no complementary effects between the datasets when testing for other clinical outcomes (Figure S2D). This marked a 23% improvement over the prediction using genetic features alone.

Since drug sensitivities may change during the progression of a disease and in response to treatment, we evaluated the survival prediction on relapsed patient samples and compared to their treatment-naive counterparts. Here, the survival models were trained by completely excluding data from both treatment-naive and relapsed samples for the patients that were tested. Interestingly, despite a major reduction in predictivity, standardizing the rAUC-log_2_ scores, but not DSS3, was able to nearly rescue the performance on relapsed samples (Figures 2D and 2E), indicating that the relative risk prediction between patients sampled from the same disease stage is preserved. Surprisingly, this was despite a significant reduction in predicted patient hazard for relapsed samples overall (Figures 2F and S2E). This suggests that whereas the cancer dependencies relevant for initial survival prognostics of high-risk patients evolve during the course of therapy, the relative patient identity characteristics remain preserved.

### Removal of confounding factors in *ex vivo* drug sensitivity profiles

Due to the major differences in clinical forecasting performance between the different drug sensitivity metrics, we performed principal component analysis (PCA; Figure S3A) to identify potential confounding factors that may cause variability in the datasets, such as artifacts associated with curve fitting, batch covariates, instrument and sample type, and biological/clinical covariates based on genetic and diagnostic phenotypes.

**Figure 3:**
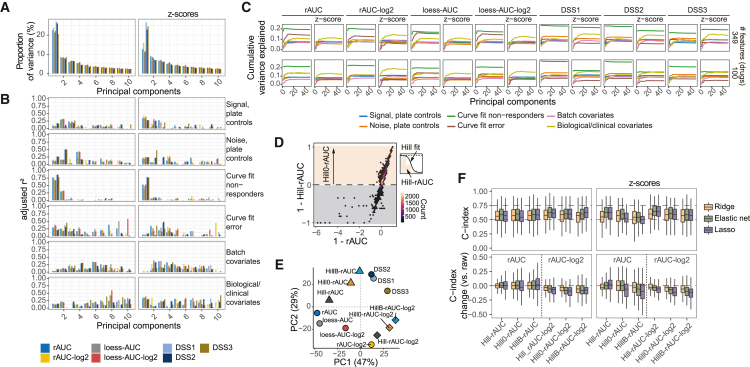
Exploring confounding factors in *ex vivo* drug sensitivity profiles (A) Percent variance explained by principal components for different drug sensitivity metrics and *Z* scores. (B) Principal component variance explained by different patient sample characteristics for different drug sensitivity metrics and *Z* scores (to the right). (C) Cumulative variance explained by different patient sample characteristics, for different drug sensitivity metrics and *Z* scores, with or without feature pre-selection based on rAUC standard deviations to remove weak and noisy drug responses. (D) Correlation between rAUC and Hill-rAUC estimated from a stepwise normalized rectangular area under the relative viability dose-response predicted by a Hill curve fit. Hill0-rAUC represents only inhibitory responses by setting all non-inhibitory values to zero. (E) PCA plot of the first two principal components indicating the similarity between the different AUC- and DSS-based drug sensitivity metrics compared in the study. The direction (sign) of all the metrics were harmonized, and the PCA was done without scaling. (F) Test C-index (200 tests) for Cox models trained on Hill-based rAUC or rAUC-log_2_ scores or corresponding Z scores (upper panels) and their computed C-index change from the non-curve fit raw rAUC or rAUC-log_2_ counterparts (lower panels).

The first principal component identified over 20% of the total variance (Figure 3A, left) and was strongly associated with curve fit non-responders (>75%, Figure 3B, left). Moreover, noise in plate controls and curve fit error had strong associations with the first two to four components of DSS1, DSS2, and DSS3, whereas the rAUCs and loess-AUCs were affected across multiple components throughout the dataset (Figure 3B, left). Strikingly, for any drug sensitivity metric, the first few components had very weak association with biological/clinical covariates, suggesting that the major cause of variability in drug sensitivities stems from patient response averages, noise, and potential curve fit issues. These effects were rectified to some extent by standardizing the drug sensitivities per patient and removing weak and noisy responses using feature pre-selection, particularly for rAUC and rAUC-log_2_ (Figures 3A–3C and S3B). These operations also increased the variance explained by biological/clinical covariates, while decreasing the effect of other sample characteristics, such as number of patient non-responders, curve fit error, and noise in the plate controls (Figures 3B and 3C). For the rAUCs and loess-AUCs metrics, these results highlighted their sensitivity to noise (represented by curve fit error), which was strongly reduced when the metrics were standardized (Figure 3C).

Interestingly, considering the strongest drug responses (with feature pre-selection) improved the relative variance explained by biological/clinical covariates only for rAUC-log_2_ and loess-AUC-log_2_ but not the DSS metrics (Figure 3C). To investigate whether this was related to loss of information from curve fitting or removing non-inhibitory responses, we performed Hill curve fitting and computed AUCs from the rectangular area under the predicted dose-responses (Hill-rAUC; Figures 3D and S3C–S3E). The Hill-rAUC was further adjusted for non-inhibitory responses (Hill0-rAUC) for a more direct comparison with the inhibition-only curve fits from Breeze protocol (HillB-rAUC) and the DSS metrics (Figure 3E). All of these metrics were also log_2_-transformed to enable comparison with the corresponding non-curve fit AUCs. These operations indicated that there was little benefit on survival prediction from curve fitting alone and a further negative influence of zero-inflating non-inhibitory dose-responses (Figure 3F). Moreover, in particular, removing variation from non-inhibitory responses replicated many of the covariance characteristics observed for the DSS metrics using PCA (Figure S3G).

**Figure 4:**
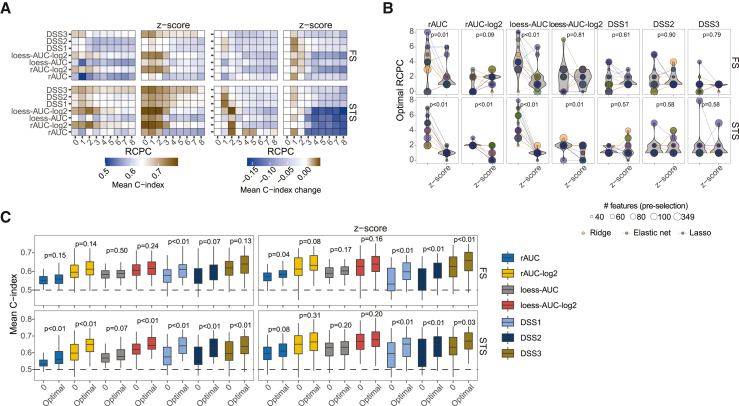
Effect of removing confounding principal components on survival prediction (A) Mean test C-index (left four panels) or C-index change (right four panels) for different survival models trained on different drug sensitivity metrics or *Z* scores with different numbers of principal components removed. The C-index changes are computed from the respective reference datasets (RCPC = 0). The values are averaged over all the C-index test scores from models with different penalties and pre-selection thresholds in Figure S4A. The lower panels represent results for short-term survival (STS) models. (B) Number of components removed to achieve the highest mean test C-index for different drug sensitivity metrics or *Z* scores shown in Figure S4A. The lower panels represent results for short-term survival (STS) models. p values were computed using a paired Wilcoxon test. (C) Mean test C-index results (50 tests) for Lasso survival models comparing zero or the optimal number of components removed for 50 datasets generated under weighted random sampling of features for different drug sensitivity metrics or *Z* scores (shown in Figure S4C). The lower panels represent results for short-term survival (STS) models. p values were computed using a paired Wilcoxon test.

To test whether the dominant covariances in the data represented confounding factors that interfered with accurate prediction of patient survival, we performed a removal of confounding principal components (RCPC) procedure.^38^ For most drug sensitivity metrics, removing at least one or two principal components improved prediction accuracy (Figures 4A, 4B, S4A, and S4B), which was more profound for short-term survival predictions. Interestingly, both log_2_-transformation and standardization appeared to negate the beneficial effect of RCPC on rAUC and loess-AUC (Figures 4B, 4C, and S4B–S4E), which compared to DSS metrics had a sharper decline in prediction performance when removing a greater number of principal components (Figures 4A and S4D), indicating loss of valuable information about patient survival. Finally, standardizing the rAUCs and loess-AUCs resulted in a significant decrease in the number of component subtractions required for achieving optimal performance for short-term survival (Figures 4B and S4E). These results indicate that processed *ex vivo* drug screening data can contain consequential confounders that may bias the dose-response curve fit and drug sensitivity scoring, and that standardization or RCPC can be used as a straightforward and reliable method for diagnosing and de-confounding the data.

### Drug sensitivity risk associations

**Figure 5:**
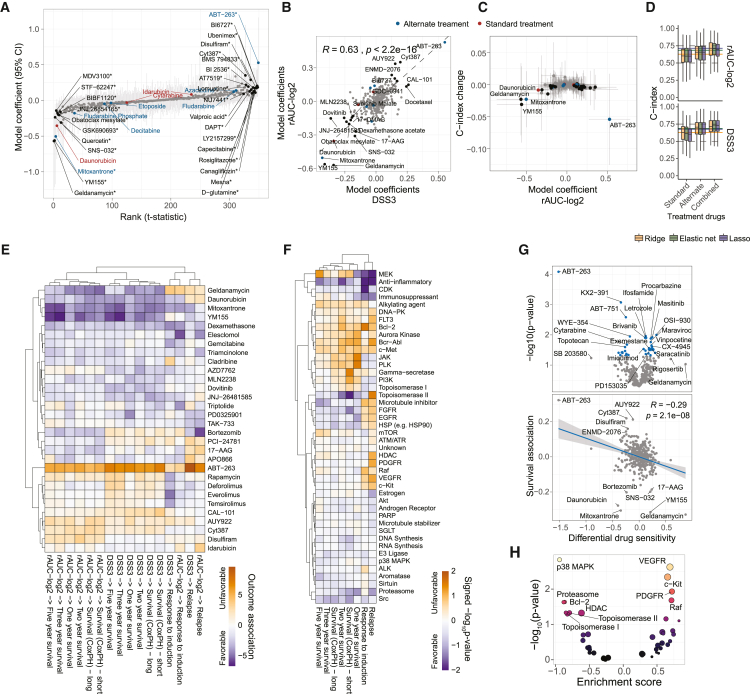
Clinical associations of *ex vivo* drug sensitivities (A) Bootstrapped ridge survival coefficients representing risk association of AUC-log_2_*Z* scores for 349 drugs. Gray vertical bars indicate 95% confidence intervals for each individual compound. Significance (∗) was determined when 95% of the bootstrapped coefficients did not include or cross zero. (B) Correlation between estimated ridge survival coefficients for rAUC-log_2_*Z* scores and DSS3 *Z* scores. The drugs with the strongest coefficients in both models are labeled. (C) Association between mean survival coefficients and mean test C-index change (50 tests) in response to drug withdrawal using ridge regression on rAUC-log_2_*Z* scores. Horizontal and vertical bars indicate standard deviations. Selected outliers are labeled. Standard or alternative AML treatment drugs are color-coded in (A)–(C). Significant drugs from (A) are color-coded as black in (C). (D) C-index results (200 tests) for Cox models trained on rAUC-log_2_ and DSS3 *Z* scores for *ex vivo* treatment drug responses. The horizontal lines indicate the respective C-index test medians for the full models in Figure 1E. (E) Clustering of normalized ridge coefficients from models trained against different clinical outcomes using rAUC-log_2_ and DSS3 *Z* scores. (F) Clustering of drug target enrichment p values based on directional drug-set enrichment on ranked coefficients from ridge models trained against different clinical outcomes using rAUC-log_2_*Z* scores. (G) Differential drug sensitivity (for rAUC-log_2_*Z* scores) between samples from relapsed and treatment-naive patients, with paired t test p values (upper) and association with ridge survival coefficients (lower). Blue dots indicate significance. The Pearson correlation coefficient (R) and p-values are indiciated in the lower panel. (H) Drug target association with differential sensitivity, using directional drug-set enrichment on differential drug sensitivities from (G).

Statistical analysis of the library-wide survival associations revealed that different models showed overall good correlation in their learned coefficients (Figures 5A, 5B, S5A, and S5B), where the strongest survival associations also corresponded to high contributions to a model’s prediction accuracy (Figures 5C and S5C). Given that many compounds in the library have overlapping targets, there was substantial redundancy in single-drug withdrawals with respect to model performance (Figure 5C). In-depth investigation of specific drug responses that were significantly associated with survival revealed that *ex vivo* sensitivity to anthracyclines predicted a favorable outcome (Figure 5A). Daunorubicin, one of the major components of 7 + 3 chemotherapy, had one of the most significant associations with survival, while idarubicin also had a favorable (albeit statistically non-significant) survival association. Epirubicin, another anthracycline used in treatment of several types of cancers, and the anthracycline analog mitoxantrone were also significantly associated with survival (Figure 5A). Interestingly, we found that *ex vivo* sensitivity to the Bcl-2/Bcl-XL/Bcl-w inhibitor ABT-263 (navitoclax) was strongly associated with increased risk (Figure 5A). ABT-263 is an experimental drug with a mechanism of action similar to the more selective Bcl-2 inhibitor venetoclax, which has been reported to significantly improve survival of AML patients who are ineligible for standard chemotherapy (please note that venetoclax had not been approved for AML therapy at the time of sample collection, which is why it was absent from the drug library).^39^ Strikingly, models trained on sets of treatment drugs alone achieved similar survival forecasting performance as the full library, and combining standard and alternate treatment drugs had a complementary effect on prediction accuracy (Figures 5D and S5D). This shows that improved prognostic value from drug sensitivity profiling can be achieved by carefully selecting drug libraries with non-redundant information about key cancer dependencies.

Hierarchical clustering of learned risk associations against different clinical outcomes revealed interesting correlations (Figures 5E and S5E). For instance, sensitivity to the mTOR inhibitors rapamycin, everolimus, deforolimus, and temsirolimus was associated with initial positive response to induction therapy but also with increased risk of relapse and decreased long-term survival. This implies that dependence on mTOR may be an early prognostic marker for acquired resistance to chemotherapy. Similarly, whereas sensitivity to daunorubicin was most positively associated with short-term survival and response to induction therapy, it was also associated with increased risk for relapse (Figure 5E).

Enrichment analysis of drug targets associated with Cox model coefficients demonstrated a library-wide risk association of sensitivity to kinase inhibitors, including JAK and PI3K signaling (Figures 5F and S5F–S5H). On the other hand, relapse risk was associated with sensitivity to multiple receptor tyrosine kinase inhibitors, and sensitivity was significantly increased in relapsed samples (Figures 5F–5H and S5F–S5I). Conversely, sensitivity to topoisomerase II-targeting chemotherapeutics in treatment-naive samples, which was associated with a favorable short-term survival and increased risk of relapse, was decreased in samples from relapsed patients (Figures 5F–5H). Consistent with previous studies,^40^^,^^41^ *ex vivo* sensitivity to agents that target anti-apoptotic proteins, particularly ABT-263, was strongly reduced in relapsed samples (Figure 5G), indicating an increased apoptotic threshold or development of cross-resistance. This was also accompanied with a library-wide negative correlation trend between differential relapse sensitivity and risk association in treatment-naive samples, where several prognostic drug markers for adverse response to standard treatment diminished in sensitivity after relapse (Figure 5G, lower panel). We conclude that *ex vivo* drug sensitivity profiles can serve as prognostic markers and may provide insight into relevant cancer dependencies and mechanisms of disease progression.

## Discussion

In this retrospective study, we used *ex vivo* drug sensitivity screening to predict the survival of AML patients subjected to standard chemotherapy. An important topic that has been the subject of much discussion is how to appropriately score drug sensitivities in large-scale drug screens. Although sigmoid-constrained curve fitting and response scoring is a valuable tool in large-scale drug screens, it is inherently sensitive to dose coverage in the dynamic range, and model parameters tend to be unreliable.^25^^,^^30^^,^^42^ Consistent with this, we observed that the EC_50_ was inferior in predicting patient survival compared to various scoring metrics based on the average dose-response efficacy. To combat the noisy nature of *ex vivo* drug screens, a common approach is to only consider the area under inhibitory Hill-response curves that cross a certain threshold, exemplified by the DSS.^27^ We also observed that this compression of drug response variability resulted in zero-inflated data with substantial loss of information. Some of this is likely due to bias associated with Hill curve fitting, introducing confounding covariation that could be countered by removing the leading principal components in the data.

An alternative approach to the DSS that worked well without or in combination with model-free curve fitting was log-transforming averages of the relative viabilities. This is a common re-scaling procedure that when used in this context effectively reduces the leverage of noisy and weak data points around one or above (non-inhibitory responses) and improves the separability of strongly inhibiting drug responses. For these drug sensitivity metrics, which retained the full range of drug response variation, patient standardization (*Z* scoring) generally improved representation of clinical information in the data even further. This operation removes patient variation in general levels of drug sensitivity, which is responsible for substantial drug-wise correlations (multicollinearity) as observed by others.^43^^,^^44^ This property has previously been linked to biological differences in gene expression and where conditioning on this feature can improve the inferences between datasets.^43^^,^^44^ Here, using RCPC to remove this feature completely improved short-term survival forecasting, indicating that it served as a negative confounder for utility of specific drug sensitivity variations. Moreover, while the burden of confounding factors may vary between datasets, the use of PCA to investigate which sample features dominate the leading components can serve as a valuable tool to determine the approximate number of components to remove.^38^ This can further be used in conjunction with other batch correcting methods to harmonize data from different centers.^45^

In our study, we used a drug library that not only includes standard chemotherapeutics but also a wide variety of other compounds, and we identified informative “drug sensitivity fingerprints” that may not only help guide treatment choice but that can also provide valuable prognostic information. Importantly, merging the data from drug sensitivity profiling with information from genetic biomarkers did not further improve the predictivity of drug profiling alone. This suggests that predictive information in genetic profiles is already captured by *ex vivo* drug sensitivities and indicates that *ex vivo* drug profiling can be further developed into a prognostic tool through rational design of focused drug libraries that fully cover cancer dependencies. Examples of drugs that should be included in such next-generation libraries for risk stratification are BCL2, mTOR, and HSP90 inhibitors, which, as already discussed above, are clearly associated with risk. Furthermore, we found that the five top-ranking drugs associated with high risk include three JAK2 inhibitors and one STAT3 inhibitor, which is consistent with previous findings that high JAK2/STAT3 activity causes resistance to chemotherapy.^46^^,^^47^^,^^48^ We are currently analyzing data from other large-scale *ex vivo* drug screening efforts, such as the FIMM and BEAT-AML datasets,^49^^,^^50^^,^^51^ which we expect will identify additional compounds associated with risk. Such analyses will not only be important for inter-center validation of data but also for development of effective harmonization methods.

Although progress has been made to update recommendations for AML risk stratification and treatment guidelines, prediction accuracies in large and heterogeneous cohorts remain modest.^4^^,^^37^ One reason for this is that AML is in part driven by non-genetic alterations,^52^^,^^53^ including epigenetic alterations and rewiring of metabolic pathways and signaling circuits, which are not easily detected by more conventional methods, such as genomics and transcriptomics. Functional *ex vivo* drug profiling may be better suited for identifying such cancer dependencies.^50^^,^^51^ Thus, with further research, we envision that *ex vivo* drug profiling can be a useful tool in the clinical decision-making process to stratify patients into treatment groups.

We conclude that *ex vivo* drug profiling reveals cellular dependencies leading to chemoresistance and cancer progression, and it robustly predicts patient survival and response to induction therapy.

### Limitations of the study

This study was conducted within a single center on a relatively homogeneous and small patient cohort. Because AML is genetically a heterogeneous disease, the mutation profiles were sparsely distributed in the patient population, which resulted in low statistical power when cross-examining genetic data with *ex vivo* drug sensitivities for patient risk group stratification and treatment response prediction. Thus, future studies on larger patient cohorts from multiple centers will be instructive for evaluating the clinical forecasting performance of drug sensitivity profiles across different age groups and sub-classes of AML. This may also allow for development of more flexible multimodal machine learning strategies that generalize across cohorts and can provide biologically informed risk categorization based on the optimal combination of genetic and drug response screen results.

In this study, we focused on variations of intensive chemotherapy given as a first-line treatment in the clinic and not on targeted therapies. The accuracy of clinical outcome predictions may vary depending on the selected therapy and the preservation of treatment-specific cancer determinants to *ex vivo* cell cultures. Given that several targeted therapies have entered the clinic, it will be important to perform similar studies on these patient cohorts. Ideally, this will identify a focused set of drugs that are highly predictive of the outcome of chemotherapy as well as targeted therapies. Using the systematic approaches that we have outlined here on more heterogeneous patient groups subjected to a wider array of treatment options may further refine the use of drug sensitivity screens as a tool for stratifying patients in appropriate treatment groups.

## STAR★Methods

### Key resources table

**Table:** 

REAGENT or RESOURCE	SOURCE	IDENTIFIER
**Biological samples**		

Patient-derived mononuclear cells isolated from bone marrow aspirates and/or peripheral blood samples	Department of Haematology at Oslo University Hospital in Norway	N/A

**Chemicals, peptides, and recombinant proteins**		

Selleck Anti-Cancer Compound Library L3000, 349 compounds	Selleck Chemicals	Z88971
Benzethonium chloride, BzCl	Santa Cruz Biotechnology	Sc-239299

**Critical commercial assays**		

LymphoPrep™ gradient centrifugation	Stemcell	# 07801
Mononuclear Cell Medium (MCM)	PromoCell	C-28030
CellTiter-Glo 2.0	Promega	G9243

**Deposited data**		

Clinical data	This study	Request to lead contact
Dose-response data (Dose response data and Hill curve fits_2023-07.csv)	This study	https://doi.org/10.5281/zenodo.10055032
Drug information data (Selleck_Drug information.xlsx)	This study	https://doi.org/10.5281/zenodo.10055032

**Software and algorithms**		

Statistical programming environment R	The R Project for Statistical Computing	https://www.r-project.org/, version 4.2.1
R scripts	This study	https://zenodo.org/doi/10.5281/zenodo.10055032

**Other**		

Echo 550 liquid handling robot	Labcyte Inc.	Center for Molecular Medicine Norway, University of Oslo, https://www.med.uio.no/english/research/core-facilities/chemical-biology-screening/
EnVision 2104 Multilabel plate reader	Perkin Elmer	N/A

### Resource availability

#### Lead contact

Further information and requests should be directed to Jorrit M. Enserink: jorrit.enserink@ibv.uio.no.

#### Materials availability

This study did not generate new unique reagents.

#### Data and code availability

• The clinical data in their complete form are freely available from the lead contact upon request.
• The drug screen data and all code is freely available at https://github.com/Enserink-lab/DSCoxTools. DOIs are listed in the key resources table.
• Any additional information required to reanalyze the data reported in this work is available from the lead contact upon request.

### Experimental model and study participant details

#### Patient cohort

Bone marrow aspirates and/or peripheral blood samples were collected from 69 adult patients diagnosed with AML and treated at the Department of Haematology at Oslo University Hospital in Norway between 2015 and 2018 (two relapse samples were collected in 2019). 60 of the patients were treatment naive and nine had undergone previous treatment (Tables S1 and S1A). From eight of the 69 patients we also received a sample at relapse. Drug screening data for the relapse samples were excluded from modeling.

The study was performed in accordance with the Declaration of Helsinki and samples were collected following written informed consent. The study was approved by the Regional Committee for Medical Research Ethics South-East Norway (REK 2015/2012). Patient data that were collected included survival, sex, age, WHO patient performance status, routine diagnostic workup consisting of flow cytometry and genetic biomarkers investigated by G-banding, RT-qPCR, FISH and fragment analysis of FLT3 and NPM1, as well as ELN2022 risk stratification and FAB classification (see Table S1 for an overview of patient characteristics). The median age was 60 years. Most patients were subjected to standard treatment, which consisted of a 30 min infusion of anthracycline [daunorubicin (60 mg/m^2^) or idarubicin (10–12 mg/m^2^)] for three days in combination with a 24-h infusion of cytarabine (Ara-C) for seven days, although the length and dosage of each treatment were in some cases altered depending on the patient’s age and general condition. Some patients received other drugs in addition to standard treatment (see Table S2 for details).

### Method details

#### *Ex vivo* drug screening

The experimental procedures were designed following methods that were state-of-the-art at the time of study start.^14^ In brief, fresh bone marrow or peripheral blood samples were collected in tubes with heparin or EDTA at the Department of Haematology at Oslo University Hospital (see Table S3). Typically, the samples were processed within 60 min after the sample was collected. In a few rare instances, when patient samples were collected at night or during the weekend, the samples were stored at 4°C for up to 16 h before processing. Mononuclear cells were subsequently purified as previously described^8^ using Lymphoprep gradient centrifugation (Stemcell) and cultured in Mononuclear Cell Medium (MCM, PromoCell C-28030) supplemented with 1% Penicillin + Streptomycin (PS) (Gibco,15140-122).

A total of 10,000 cells (25 μL) in MCM + PS was added to each well in pre-drugged 384-well plates (Greiner Bio-One) using a Multi Drop Combi peristaltic dispenser (Thermo Scientific). The number of cells was adjusted for specific samples with lower cell counts without a noticeable change in the outcome of the experiments. The Selleck Anti-Cancer Compound Library L3000 was used for drug screening, which consists of 349 anti-cancer drugs dissolved in dimethyl sulfoxide (DMSO) (see Table S2 for the complete list). The compounds were distributed in seven 384-well plates using five 10-fold dilution steps (1 nM–10.000 nM). Eight positive (benzethonium chloride, BzCl) and negative (DMSO only) controls were added to each plate. Drug handling was performed at the Center for Molecular Medicine Norway, University of Oslo, using an Echo 550 liquid handling robot (Labcyte Inc.). After incubation for 72 h at 37°C in a humidified environment with 5% CO_2_, relative cell viability was quantified using the CellTiter-Glo 2.0 Cell Viability Assay (Promega), as a fast, sensitive, and high-throughput friendly readout, which is also used as a standard readout in other centers.^14^^,^^54^ The luminescence readout was measured in an EnVision 2104 Multilabel plate reader (PerkinElmer) in counts per second (CPS). Relative cell viability was computed from CPS values normalized to negative (DMSO) and positive (BzCl) controls within each plate.

#### Dose-response analysis

Relative cell viabilities (*y*) were computed from CPS values using min-max normalization to the median CPS of the positive and negative controls within each plate. All negative response values were adjusted to zero. Plate quality was assessed by a Z′-factor and strictly standardized mean difference (SSMD) of the plate controls. The Z′-factor measures the difference between positive plate controls (BzCl) and negative plate controls (DMSO). These controls were included in each assay plate for each patient sample. A Z′-factor between 0.5 and 1 is considered excellent, values between 0 and 0.5 may be acceptable, while values below 0 are indicative of a poor essay.^55^ The average drug sensitivity was measured as a normalized rectangular area under the curve (rAUC) of the raw relative viability response for every patient-drug combination using the formula:

$$rAUC=\sum_{c=2}^{5}\frac{y_{c}\left(\log\;x_{c}-\log x_{c-1}\right)}{\log\;x_{5}-\log x_{1}}$$

Here *x*_*c*_ indicates a given concentration with relative viability *y*_*c*_, ranging from the lowest concentration *x*_*1*_ to the highest concentration *x*_*5*_. Before their use in models all rAUCs were centered around zero inhibition: 1−rAUC. Alternatively, the rAUCs were negative log_2_-transformed ($–log_{2}\left(rAUC\right)$) to counter the skewness of the relative viability scale.

To score drug sensitivities using metrics based on dose-response curve fitting we used routines adapted from the Breeze application.^23^ Briefly, Breeze transforms the scale to percent inhibition and the following parametrization of the Hill equation is fitted to the data:

$$f\left(x\right)=R_{\min}+\frac{\left(R_{\max}-R_{\min}\right)}{1-10^{n\left(logEC50-logx\right)}}$$

where $R_{\min}$ is the baseline inhibition (typically held to zero), $R_{\max}$ is the maximum inhibitory response for the drug, and n is the slope (Hill coefficient). In Breeze, only growth inhibitory responses are considered. The algorithm constrains the curve fits with $R_{\min}=0$, n=[0.1,2.5] and $R_{\max}=\left[0,100\right]$. All EC50s are constrained to a value between the maximum and minimum concentration of the experiment. EC50 values for non-inhibitory responses are set to the maximum concentration. TEC50 is computed by setting EC50 to the maximum concentration for the models with R_max_ under 25%. Before further use, both EC50 and TEC50 were log-transformed and mean-subtracted per drug.

The drug sensitivity score (DSS) is computed from an analytical solution to the integral ,

$$I=\int_{x_{t}}^{x_{5}}f\left(x\right)dx$$

where *x*_*t*_ is the concentration at an activity threshold *t* set to 10%. Thus, DSS can only take positive values with $DSS1=\frac{\left(I-t\left(\log\;x_{5}-\log x_{t}\right)\right)}{\left(\left(100-t\right)\left(\log\;x_{5}-\log x_{1}\right)\right)}$, $DSS2=DSS\frac{1}{\log\;R_{\max}}$, and $DSS3=DSS2\frac{\left(x_{5}-x_{t}\right)}{\left(x_{5}-x_{1}\right)}$^27^.

We also used Breeze to compute an AUC based on a LOESS curve fit (referred to as loess-AUC), which models both growth promoting and inhibitory responses. For bidirectional Hill curve fitting, we modified the Breeze algorithm by setting $R_{\max}=\left[-100,100\right]$, and thus allowing fitting of non-inhibitory responses. Hill-rAUC was computed by predicting the relative viability and using the rAUC formula as indicated before.

Patient-wise standardization of drug sensitivity metrics (*s*), was performed as follows to compute a drug sensitivity *Z* score:

$$z_{dr,pt}=\frac{s_{dr,pt}-\mu_{pt}}{\sigma_{pt}}$$

Here μ_pt_ and σ_pt_ is the drug sensitivity mean and standard deviation per patient respectively. For scaling of the drugs, a drug-wise standardization procedure was done over the drug sensitivity metrics or drug sensitivity z-scores.

#### Clinical data processing

The clinical data were grouped into two feature sets, with binarized dummy variables for categorical data. Clinical prognostic features included age at the time of diagnosis, sex, WHO patient performance status, and ELN2022 risk stratifications. Genetic features included specific mutations and chromosomal rearrangements with coverage for at least three patients. Missing data were zero-imputed. The survival times were computed from the date of diagnosis until the registered date of death, and censoring times were computed to the last recorded visiting date. Response to induction was binarized based on reaching blast clearance (remission) or not after induction therapy. Relapse was binarized for the sub-population of patients having reached complete remission.

#### Model training and cross-validation

Regularized Cox models were trained on the different drug sensitivity metrics and the binarized clinical variables using the glmnet package.^56^ Ridge, Lasso, or Elastic net models were trained by setting the penalty mixture parameter (***α***) to 0, 1, or 0.4, respectively, unless otherwise specified, and screening over a sequence of penalties using leave-one-out cross-validation (due to the low sample size). The model with the best average cross-validation (partial likelihood) deviance score was selected for testing or further analysis of model coefficients. The Cox model coefficients represent the change in log-hazard ratio (log-HR) as a function of drug sensitivity. Regularized binomial models were used for logistic regression of binarized clinical outcomes, and optimized using the same procedure.

Pre-selection of model features prior to model training was based on thresholds for standard deviations in drug sensitivity (as indicated), where the number of drugs with the greatest drug sensitivity standard deviations were selected. For a random sampling of features (p = 100), the rAUC standard deviation was used as sampling weights.

#### Model testing

For testing the models, a random sample of 10 patients was withheld from the training procedure, with a fixed proportion of deceased to surviving patients to maintain the approximate proportions in the complete dataset (5 deceased and 5 surviving for the full survival models, and 4 deceased and 6 surviving for the short term survival models). The prediction accuracy on test data was scored with Harrell’s concordance index (C-index), which defines the proportion of patient pairs with concordance between their observed survival times and predicted risk. It is especially suited for clinical studies as it takes into account the censoring of patients where data is not recorded after a given point.^57^ The training and testing procedure was repeated 50 or 200 times, as indicated. For testing of models predicting binary clinical outcomes, a 5-fold training and testing procedure was performed and prediction accuracy was measured using an area under the receiver operating characteristic curve (ROC AUC).

#### Analysis of confounding factors

Principal component analysis (PCA) was performed using singular value decomposition (SVD) on scaled matrices of drug sensitivity metrics or z-scores, and removal of confounding principal components (RCPC) was performed by reconstructing the datasets after removing the leading singular values and singular vectors, followed by rescaling of the metrics (see Figure S3A). SVD was performed on scaled matrices of drug sensitivity metrics (or z-scores) such that $\frac{\left(s_{dr,pt}-\mu_{dr}\right)}{\sigma_{dr}}=\sum_{k}u_{dr,k}{\sqrt{\lambda }}_{k}v_{k,pt}$, where *u*_*dr,k*_ and *v*_*k,pt*_ are components of ***u***_*k*_ and ***v***_*k*_ which are left and right singular vectors of the principal axis *k* with variance $\lambda_{k}$. The variance explained by each principal component was computed as $\rho_{k}=\frac{\lambda_{k}}{\sum_{k}\lambda_{k}}$.

The association of specific sample characteristics with a principal component was assessed using linear regression against ***v***_*k*_ and measured with the adjusted r-squared ($r_{adj}^{2}$). The patient sample characteristics with the following features were evaluated: log average CPS signal for DMSO and BzCl; average CPS noise measured as the coefficient of variation for DMSO and BzCl as well as their SSMD; the number of curve fit non-responders measured as the number of DSS3 values equal zero or EC50 or TEC50 values at maximum; curve fit error features, which included the number of low-confidence curve fits reported by Breeze, and the average log-EC50 standard error, mean absolute error and max residual error per patient reported by Breeze; batch covariates, which included time period clusters for screen executions (defined using k-means clustering), instrument batch, cell seeding number (over or under 10000 cells) and blast source (PBMC or BM); biological/clinical covariates, which included genetic and cytogenetic features, age, sex, ELN2022 risk stratifications, clinical history (primary AML or secondary after antecedent myelodysplastic syndromes or other causes), and AML FAB classifications. Cumulative variance explained by a sample characteristic up to a component K was calculated as $\sum_{k=1}^{K}\rho_{k}r_{adj,k}^{2}$.

PCA on dose-response data was done such that $\frac{\left(y_{\left(dr,c\right),pt}-\mu_{\left(dr,c\right)}\right)}{\sigma_{\left(dr,c\right)}}=\sum_{k}u_{\left(dr,c\right),k}{\sqrt{\lambda }}_{k}v_{k,pt}$, where *y*_*(dr,c),pt*_ represent the raw, processed or Hill fitted drug response or curve fit residuals of a patient (columns) and a drug at a specific concentration (rows). When indicated, standardization was applied over the columns.

To test the removal of potentially confounding principal components, the drug sensitivity metrics (or z-scores) were reconstructed as ${s^{\prime }}_{dr,pt}=\sigma_{dr}\sum_{k>l}u_{dr,k}{\sqrt{\lambda }}_{k}v_{k,pt}+\mu_{dr}$, where *l* represents the minimum number of principal components removed. RCPC testing was performed for different Cox models and different dataset sizes based on the feature pre-selection described earlier (Figures 4A, 4B, S4A, and S4B) and for Lasso trained on 50 randomly sampled datasets (Figures 4C and S4C—S4E).

#### Variable importance

The importance of individual drugs was assessed by their contribution to predictivity through a variable withdrawal test, and the statistical significance of the model coefficients. The contribution to predictivity was measured by training and testing models leaving out individual drugs one-by-one and computing the change in C-index from the model containing all drugs. The statistical significance of model coefficients was estimated by training 200 models on bootstrapped datasets and computing the mean and confidence interval for the fitted coefficients.

#### Model coefficient clustering

Estimated Ridge coefficients from models predicting different clinical outcomes were normalized by the standard deviation, and drugs with a normalized coefficient greater than three were selected for hierarchical clustering using euclidean distance and ward.D2. Before the clustering, the sign of coefficients for the different models were harmonized such that positives and negatives were associated with unfavorable and favorable clinical outcomes, respectively.

#### Enrichment test

Parametric enrichment analysis was performed using the GSEA function from ClusterProfiler based on ranked Ridge model coefficients or differential drug sensitivity z-scores, where drug sets were defined based on drug target or class association.^58^

### Quantification and statistical analysis

Statistical significance was assessed using paired Wilcoxon test for Figures 2F, 3B, 3C, S2E, S4B, and S4E. For predicted hazard ratio comparison between relapse and treatment-naïve samples in Figures 2F and S2E, data points were paired based on patient identity (n = 8). For the other Fig., data points were paired based on methodological parameters (Figures 3B and S4B) or random sample identity (Figures 3C and S4E). Significance was indicated for p values ≤0.05.

The similarities in drug sensitivity profiles between drugs or between samples/patients in Figures 1F, S1C, S1D, S1E, S1C, and S3E, were measured using the Pearson correlation coefficient (PCC, or denoted as *R* in the scatterplots).

Statistical significance of drug-wise survival associations based on Ridge model coefficients in Figures 5A and S5, was determined based on the 95% confidence intervals, where significance (∗) was determined when 95% of the bootstrapped coefficients did not include or cross zero.

For analysis of differential drug sensitivity z-scores between relapse and treatment-naïve samples in Figure 5G, statistical significance was evaluated using a paired t-test, with pairing based on patient identity (n = 8). Significance was indicated for p values ≤0.05. Statistical significance of enriched drug sets in Figures 5F, 5H, and S5F—S5I were assessed using the permutation test option of GSEA.

## Supplemental information

Document S1. Figures S1–S5 and Tables S1–S3

Document S2. Article plus supplemental information
