# Supplementary material for: Clinical forecasting of acute myeloid leukemia using ex vivo drug-sensitivity profiling
Source: Cell Rep Methods. 2023 Dec 7;3(12):100654. doi: 10.1016/j.crmeth.2023.100654 (PMC10753296; doi:10.1016/j.crmeth.2023.100654)
Supplement: Document S1. Figures S1–S5 and Tables S1–S3 [file mmc1.pdf]

Cell Reports Methods, Volume *3*

Supplemental information

Clinical forecasting of acute myeloid leukemia using *ex vivo* drug-sensitivity profiling

Aram N. Andersen, Andrea M. Brodersen, Pilar Ayuda-Durán, Laure Piechaczyk, Dagim Shiferaw Tadele, Lizet Baken, Julia Fredriksen, Mia Stoksflod, Andrea Lenartova, Yngvar Fløisand, Sigrid S. Skånland, and Jorrit M. Enserink

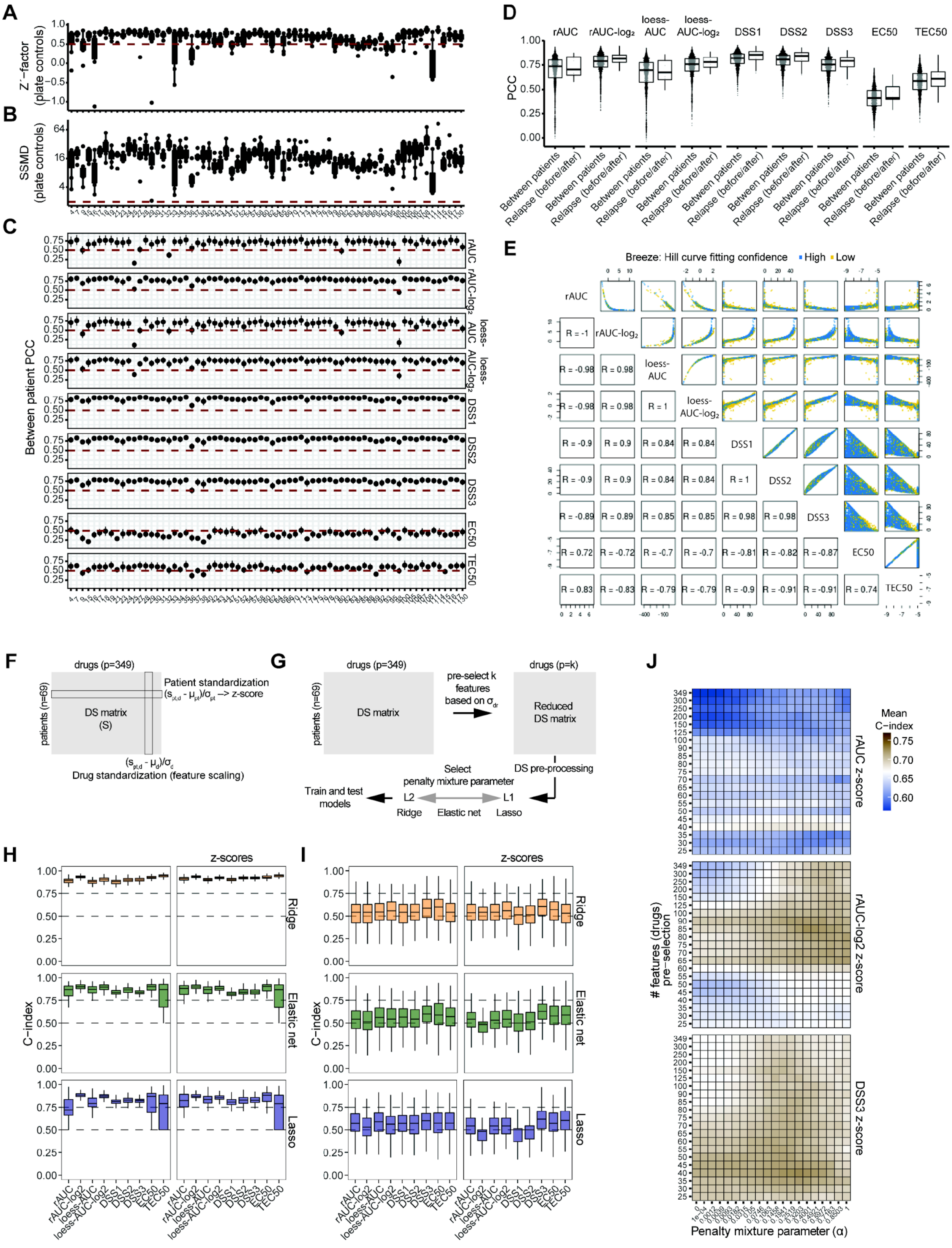

Figure S1. Drug screen quality controls, related to Figure 1. ***A,*** Z′-factor for DMSO and BzCl plate controls per patient. ***B,*** SSMD for DMSO and BzCl plate controls per patient. ***C,*** Mean between-patient drug sensitivity profile correlation per patient for different drug

sensitivity metrics. Dot and bar represent the mean and standard deviation for one patient against the remaining cohort. ***D,*** Correlations in drug sensitivity profiles, either between patients or for the same patient before and after relapse. ***E,*** Correlations between the different drug sensitivity metrics. High and low confidence Hill curve fits reported by Breeze are color-coded. ***F,*** Standardization procedures used in the study. Drug sensitivity z-scores were generated by standardizing each patient distribution. Drug sensitivity feature scaling was done by standardizing each drug distribution. ***G,*** Overview of feature pre-selection procedure and penalty type testing. ***H,*** Training C-index results (200 tests) for Cox models trained on different drug sensitivity metrics (*left*) or drug sensitivity z-scores (*right*). ***I,*** Test C-index results (200 tests) for Cox models trained on different drug sensitivity metrics (*left*) or drug sensitivity z-scores (*right*) with feature scaling (drug-wise standardization). ***J,*** Mean test C-index results (50 tests) for Cox models trained on rAUC, rAUC-$\log_2$, or DSS3 z-scores with various penalty mixture parameters (α) and feature pre-selection thresholds based on standard deviations of the respective drug sensitivity metric (rAUC, rAUC-$\log_2$, or DSS3).

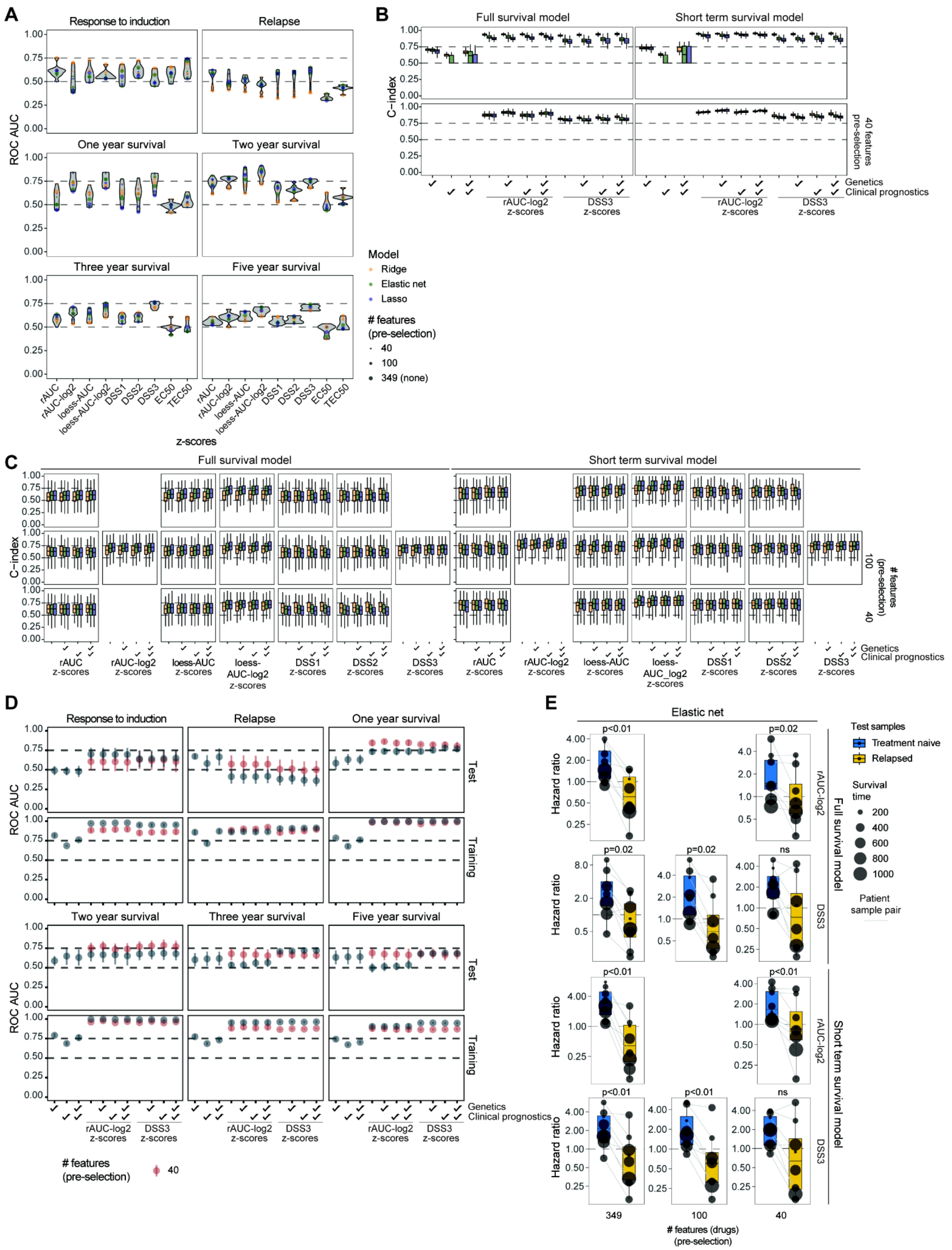

Figure S2. Supplement to versatility of drug sensitivity profiling for clinical outcome predictions, related to Figure 2. ***A,*** Average ROC-AUC score from 5-fold testing of classification of various binarized clinical outcomes using different drug sensitivity metrics,

model penalties and pre-selection thresholds. ***B,*** Training C-index results (200 tests) for Cox models trained on different dataset compositions based on clinical feature sets and rAUC-log_2 or DSS3 z-scores. The lower panels represents pre-selection of 40 features based on rAUC standard deviations, and the right panels represent prediction results from short term survival modeling. ***C,*** Test C-index results (200 tests) for Cox models trained on different dataset compositions based on clinical feature sets and drug sensitivity z-scores with different feature pre-selection cutoffs. Results from short term survival modeling are shown on the right. ***D,*** Testing and training ROC-AUC scores for classification of various binarized clinical outcomes using Ridge models trained on different dataset compositions based on clinical feature sets and rAUC-log_2 or DSS3 z-scores. The dot and bar indicate mean and standard deviation from 5-fold testing and training. E, Predicted hazard ratio (relative to population-median) on treatment-naive and relapsed samples for Elastic Net survival models trained on rAUC-log_2 or DSS3 z-scores with or without feature pre-selection. Bottom two panel rows represent short term survival modeling.

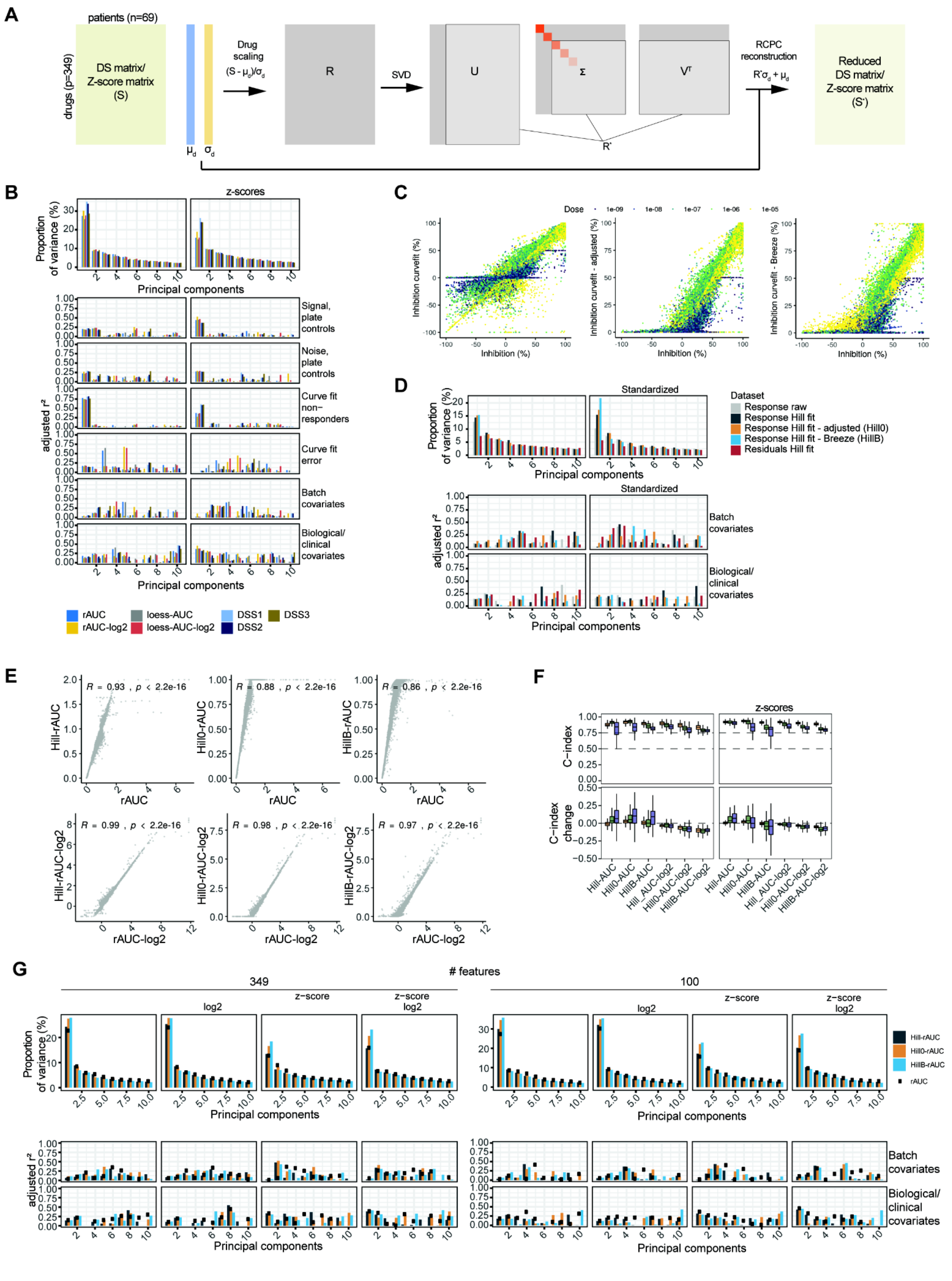

Figure S3. Supplement to exploration of confounding factors with PCA, related to Figure 3. ***A,*** Overview of PCA procedure based on SVD, and reconstruction using RCPC. ***B,*** Percent variance explained by principal components (*upper panels*) and principal component

variance explained by different patient sample characteristics (*lower panels*) for different drug sensitivity metrics and z-scores with feature pre-selection (100 features) based on rAUC standard deviations. ***C,*** Comparison between observed inhibition and Hill curve predicted inhibition. *Left panel* compares with Hill curve fitting for both inhibitory and non-inhibitory drug responses, *middle panel* compares with Hill curve fitting where non-inhibitory responses are set to zero, and the *right panel* compares with Hill curve fitting of inhibitory responses using Breeze. ***D,*** Percent variance explained by principal components (*upper panels*) and principal component variance explained by different patient sample characteristics (*lower panels*) for different non-standardized (*left*) and standardized (*right*) dose response datasets from ***C***, as well as the Hill curve fitting residuals. ***E,*** Comparison between Hill-based rAUC or rAUC-log_2 scores with non-curve fit raw rAUC or rAUC-log_2 scores. ***F,*** Training C-index results (200 tests) for Cox models trained on Hill-based rAUC or rAUC-log_2 scores or corresponding z-scores (*upper panels*), and their computed C-index change from the non-curve fit raw rAUC or rAUC-log_2 counterparts (*lower panels*). ***G,*** Percent variance explained by principal components (*upper panels*) and principal component variance explained by different patient sample characteristics (*lower panels*) for different Hill-based rAUC or rAUC-log_2 scores or corresponding z-scores with or without feature pre-selection based on rAUC standard deviations. The dots represent the corresponding results from the non-curve fit raw rAUC or rAUC-log_2 counterparts.

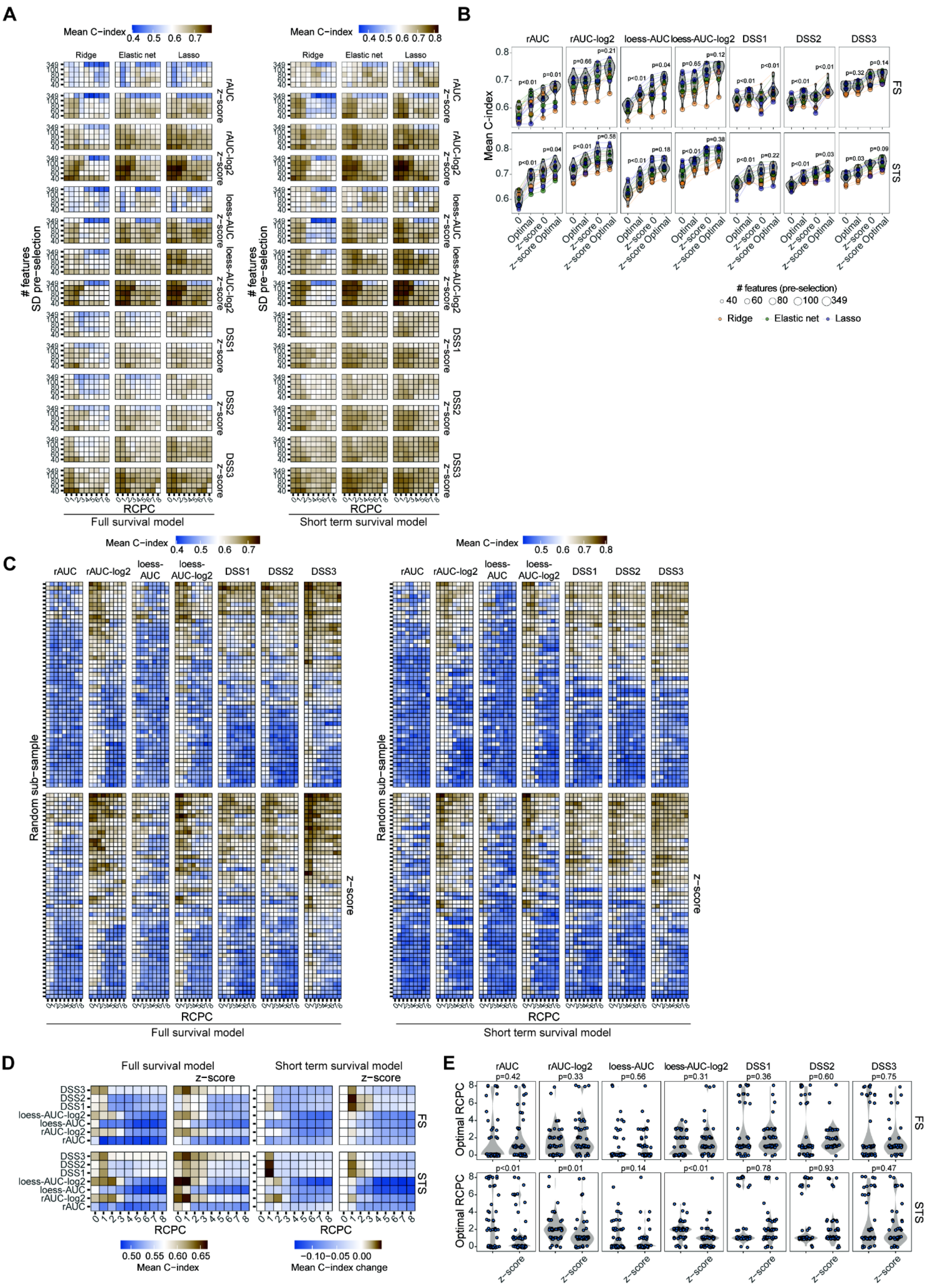

Figure S4. Supplement for testing removal of confounding principal components, related to Figure 4. ***A*,** Mean test C-index results (50 tests) for Ridge, Elastic net, and Lasso

survival models trained on different drug sensitivity metrics or z-scores with various feature pre-selection thresholds (based on rAUC standard deviations), and different numbers of principal components removed. *Left panels* represent results for full survival models and *right panels* represent results for short term survival models. ***B,*** Mean test C-index results (50 tests) for different survival models comparing zero or the optimal number of components removed for different drug sensitivity metrics or z-scores (shown in S6A). The *lower panels* represent results for short term survival models. p-values were computed using a paired Wilcoxon test. ***C,*** Mean test C-index results (50 tests) for Lasso survival models trained on 50 datasets generated under weighted random sampling of features, using different drug sensitivity metrics or z-scores, and different numbers of principal components removed. *Left panels* represent results for full survival models and *right panels* represent results for short term survival models. ***D,*** Sample average for test C-index (*left four panels*) or C-index change (*right four panels*) from the results in S6C. The C-index changes are computed from the respective reference datasets (RCPC = 0). ***E,*** Number of components removed to achieve the highest mean test C-index for different drug sensitivity metrics or z-scores in S6C. The *lower panels* represent results for short term survival models (STS). p-values were computed using a paired Wilcoxon test.

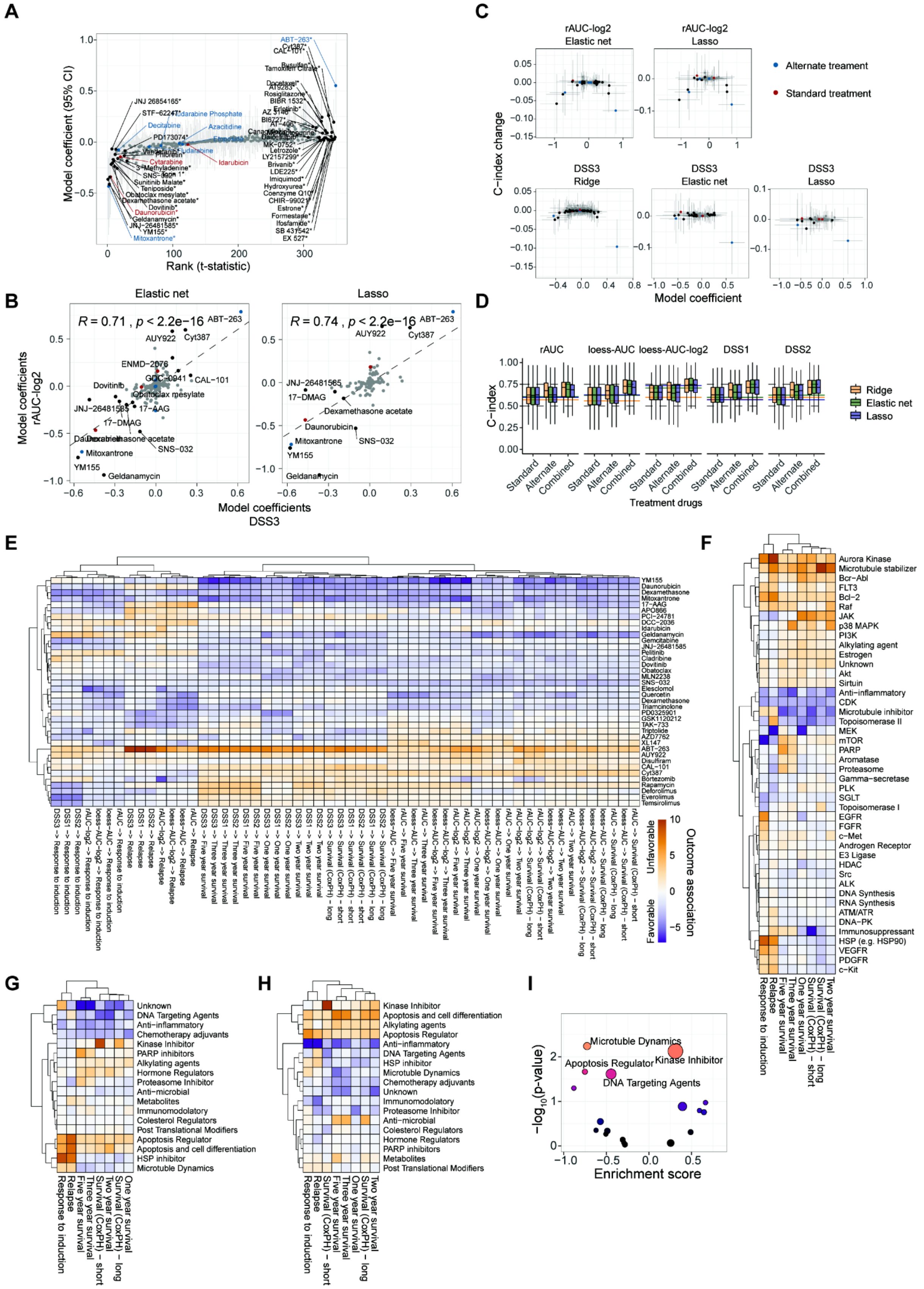

Figure S5 Supplement to clinical associations of *ex vivo* drug sensitivities, related to Figure 5. ***A,*** Bootstrapped Ridge survival coefficients representing risk association of DSS3

z-scores for 349 drugs. The vertical bars indicate the 95% confidence interval. Significance (*) was determined when 95% of the bootstrapped coefficients did not include or cross zero. ***B,*** Correlation between estimated Lasso and Elastic net survival coefficients for rAUC-$\log_2$ z-scores or DSS3 z-scores. The drugs with the strongest coefficients in both models are labeled. ***C,*** Association between mean survival coefficients and mean test C-index change (50 tests) in response to drug withdrawal for different models. The horizontal and vertical bars indicate the standard deviations, respectively. Standard or alternate AML treatment drugs are color-coded in *A-C*. The outliers are the same as marked in figure B. ***D,*** C-index results (200 tests) for Cox models trained on different drug sensitivity z-scores for treatment drugs. The horizontal lines indicate the respective C-index test medians for the full models in *Fig. 1E*. ***E,*** Clustering of normalized Ridge coefficients from models against different clinical outcomes using different drug sensitivity z-scores. ***F,*** Clustering of drug target enrichment p-values based on directional drug-set enrichment on ranked coefficients from Ridge models against different clinical outcomes using DSS3 z-scores. ***G,*** Clustering of drug class enrichment p-values based on directional drug-set enrichment on ranked coefficients from Ridge models against different clinical outcomes using rAUC-$\log_2$ z-scores. ***H,*** Clustering of drug class enrichment p-values based on directional drug-set enrichment on ranked coefficients from Ridge models against different clinical outcomes using DSS3 z-scores. ***I,*** Drug class association with differential sensitivity, using directional drug-set enrichment on differential drug sensitivities from *Fig. 5G*.

Table S1, Patient characteristics, related to STAR Methods.

ID	Age	Sex	Leukemia status	FAB class	Karyotype	ELN2022
4	40-49	Female	Primary	M1	Normal	Intermediate
7	50-59	Male	Primary	M2	Normal	Intermediate
9	60-69	Male	Primary	M5	Other	Intermediate
15	60-69	Male	Primary	M2		Adverse
16	60-69	Female	Secondary	Other	Normal	Intermediate
17	60-69	Male	Secondary	Other	Normal	Intermediate
18	40-49	Female	Primary	M1	Complex	Adverse
19	60-69	Female	Primary	M1	Normal	Intermediate
21	60-69	Male	Primary	M1		Intermediate
23	60-69	Female	Primary	M4	Normal	Intermediate
24	<40	Female	Primary	M1	Other	Intermediate
25	>70	Male	Unknown	M0		Favorable
27	60-69	Female	Secondary	M4/M5	Normal	Intermediate
28	50-59	Male	Primary	M4/M5	Normal	Intermediate
29	50-59	Female	Primary	M4 eos	Other	Favorable
30	40-49	Female	Primary	M4	Normal	Intermediate
31	50-59	Female	Primary	M2	Normal	Intermediate
32	60-69	Male	Primary	M1	Normal	Intermediate
33	60-69	Female	Primary	M2	Complex	Adverse
34	60-69	Male	Primary	M1	Normal	Intermediate
35	60-69	Female	Secondary		Complex	Adverse
36	<40	Female	Primary	M5	Normal	Intermediate
37	50-59	Male	Primary	M0	Other	Adverse
38	60-69	Male	Primary	M0	Normal	Intermediate
40	60-69	Male	Primary	M5	Other	Intermediate
42	60-69	Female	Primary	M2	Other	Adverse
43	60-69	Male	Secondary	M2	Normal	Intermediate
44	60-69	Male	Primary	M6	Normal	Favorable
47	40-49	Male	Primary	M5	Other	Adverse
51	40-49	Female	Primary	M4		Favorable
52	<40	Female	Primary	M1		Adverse
54	60-69	Female	Secondary	M4	Normal	Favorable
57	50-59	Female	Secondary	M5	Normal	Favorable
58	50-59	Female	Primary	M4	Normal	Intermediate
60	60-69	Female	Primary	M3	Other	Favorable
62	60-69	Female	Secondary	M5	Normal	Intermediate
64	50-59	Female	Primary	M5	Other	Intermediate
65	60-69	Male	Primary	M5	Normal	Adverse
66	60-69	Male	Primary	Other	Other	Adverse
70	50-59	Female	Primary	M3	Normal	Favorable

71	40-49	Female	Primary	M5	Other	Intermediate
73	<40	Female	Primary	M2	Other	Adverse
74	50-59	Male	Primary	M4	Other	Adverse
75	40-49	Female	Primary	M1	Normal	Intermediate
76	50-59	Male	Primary	M1	Normal	Intermediate
78	60-69	Male	Secondary	M4	Normal	Intermediate
79	50-59	Male	Primary	M7	Other	Favorable
80	<40	Male	Primary	M6	Normal	Favorable
82	60-69	Male	Primary	Other	Complex	Adverse
83	>70	Female	Unknown		Normal	Intermediate
84	60-69	Female	Primary	M2	Other	Favorable
85	40-49	Female	Primary	M0	Other	Adverse
88	>70	Female	Primary	M4	Normal	Intermediate
89	50-59	Female	Primary	M2	Normal	Favorable
92	<40	Female	Primary	M1	Normal	Intermediate
93	<40	Male	Primary	M2	Normal	Favorable
95	50-59	Female	Primary	M4/M5	Normal	Favorable
98	50-59	Male	Secondary	Other	Normal	Intermediate
100	50-59	Male	Primary	M4 eos	Other	Intermediate
105	60-69	Male	Primary	M1/M2	Complex	Adverse
106	>70	Female	Primary	M1/M2	Normal	Intermediate
107	>70	Male	Primary	M4/M5	Other	Favorable
108	50-59	Male	Primary	M3	Normal	Favorable
111	60-69	Female	Primary		Normal	Intermediate
114	40-49	Female	Primary	M1/M2	Other	Adverse
115	60-69	Male	Primary	M1/M2	Complex	Adverse
116	60-69	Male	Primary	M1/M2	Normal	Intermediate
117	>70	Male	Primary		Other	Adverse
130	>70	Female	Secondary	Other	Other	Adverse

Table S2, Overview of treatment and disease course, related to STAR Methods.

ID	Induction	HMAS	Allogenic BM transplant	Persistent leukemia post first induction treatment	Relapse	Treatment Naive	Cures given
100	Standard therapy	Yes	No	No	No	Yes	1-2
105	Standard therapy	No	Yes	No	No	Yes	1-2
106	Standard therapy	No	No	Yes	Yes	Yes	3-5
107	Standard therapy	No	No	No	No	Yes	1-2
108	Other	No	No	No	No	Yes	1-2
111	Standard therapy	Yes	No	No	Yes	Yes	1-2
114	Standard therapy	No	Yes	Yes	Yes	Yes	1-2
115	Standard therapy	No	Yes	Yes	Yes	Yes	3-5
116	Standard therapy	No	Yes	Yes	Yes	Yes	3-5
117	Standard therapy	No	No	Yes	Yes	Yes	3-5
130	Other	No	No	Yes	Yes	No	3-5
15	Standard therapy + Lenalidomide	No	Yes	No	No	Yes	1-2
16	Other	No	No	No	Yes	Yes	1-2
17	Standard therapy	No	Yes	No	Yes		1-2
18	Standard therapy	No	Yes	No	No	Yes	1-2
19	Standard therapy	No	Yes	No	Yes	Yes	3-5
21	Standard therapy	No	No	No	Yes	Yes	1-2
23	Standard therapy + Lenalidomide	No	No	No	No	Yes	1-2
24	Standard therapy + Lenalidomide	No	Yes	No	No	Yes	1-2
25	Standard therapy	No	No	Yes	No	Yes	1-2
27	Standard therapy	No	Yes	Yes	Yes		3-5
28	Standard therapy	No	Yes	No	Yes	No	3-5
29	Standard therapy + Lenalidomide	No	No	No	No	Yes	1-2
30	Standard therapy	No	No	No	Yes	Yes	3-5
31	Standard therapy	No	Yes	No		Yes	3-5
32	Standard therapy	No	Yes	Yes	No	Yes	3-5
33	Standard therapy	No	No	No	No	Yes	1-2
34	Standard therapy	No	Yes	No	Yes	Yes	3-5
35	Other	No	Yes	Yes	Yes	Yes	3-5
36	Standard therapy	No	Yes	Yes	Yes	No	3-5
37	Standard therapy	No	No	No	No	Yes	1-2
38	Standard therapy + Lenalidomide	No	Yes	No	Yes	Yes	1-2

4	Standard therapy	Yes	No	No	No	Yes	3-5
40	Standard therapy	No	Yes	No	No	Yes	1-2
42	Standard therapy	No	No	Yes	Yes	Yes	3-5
43	Standard therapy	No	Yes		Yes	No	3-5
44	Standard therapy + Lenalidomide	Yes	No	No	No	Yes	1-2
47	Standard therapy	No	Yes	No	Yes	No	Unknown
51	Standard therapy + Lenalidomide	Yes	Yes	No	Yes	Yes	3-5
52	Standard therapy	No	No	Yes	Yes	Yes	3-5
54	Standard therapy	No	No	No	No	Yes	1-2
57	Standard therapy + Lenalidomide	Yes	No	No	Yes	No	1-2
58	Standard therapy + Lenalidomide	Yes	Yes	No	Yes	Yes	3-5
60	Other	No	No	No	No	Yes	1-2
62	Standard therapy	No	No	No	No	Yes	1-2
64	Standard therapy + Lenalidomide	No	Yes	Yes	No	Yes	1-2
65	Standard therapy	No	Yes	No	No	Yes	1-2
66	Standard therapy	No	Yes	No	No	Yes	1-2
7	Standard therapy	No	Yes		Yes	No	Unknown
70	Standard therapy	No	No	Yes	No	Yes	1-2
71	Standard therapy	Yes	No	No	Yes	Yes	3-5
73	Standard therapy	No	Yes	No	No	Yes	1-2
74	Standard therapy + Lenalidomide	No	Yes	No	Yes	Yes	3-5
75	Standard therapy	Yes	Yes	No	Yes	Yes	3-5
76	Standard therapy	No	Yes	No	Yes	Yes	3-5
78	Standard therapy	No	Yes	No	No	Yes	1-2
79	Standard therapy	Yes	No	No	No	Yes	1-2
80	Standard therapy	Yes	No	No	No	Yes	1-2
82	Other		Yes				1-2
83	Unknown		No				Unknown
84	Standard therapy + Lenalidomide	No	No	No	No	Yes	1-2
85	Standard therapy	No	Yes	No	No	Yes	3-5
88	Standard therapy	No	No	No	Yes	Yes	3-5
89	Standard therapy	Yes	No	No	No	Yes	1-2
9	Standard therapy + Lenalidomide	Yes	No	No	No	Yes	3-5
92	Standard therapy	Yes	No	No	No	Yes	1-2

93	Standard therapy + Lenalidomide	No	Yes	No	Yes	Yes	1-2
95	Standard therapy	Yes	No	No	No	Yes	1-2
98	Other	No	No	Yes	Yes	Yes	1-2

Table S3, Sample and batch information, related to STAR Methods.

ID	Sample ID	Source	Instrument	Instrument serial number	Cells_per_well
100	Patient 100	BM	VictorX	20302104	10000
105	Patient 105	Blood	VictorX	20302104	10000
106	Patient 106	BM	VictorX	20302104	10000
107	Patient 107	Blood	VictorX	20302104	10000
108	Patient 108	BM	VictorX	20302104	10000
111	Patient 111	BM	VictorX	20302104	10000
114	Patient 114	BM	VictorX	20302104	10000
115	Patient 115	BM	VictorX	20302104	406
115	Patient 115_relapse	BM	EnVision	1020030	
116	Patient 116	BM	VictorX	20302104	10000
117	Patient 117	BM	VictorX	20302104	10000
130	Patient 130	BM	VictorX	20302104	2000
15	Patient 15	BM	EnVision	1040142	2200
16	Patient 16	Blood	EnVision	1040142	10000
17	Patient 17	Blood	EnVision	1040142	10000
18	Patient 18	BM	EnVision	1040142	10000
19	Patient 19	BM	EnVision	1040142	10000
21	Patient 21	Blood	EnVision	1040142	10000
23	Patient 23	BM	EnVision	1040142	10000
24	Patient 24	BM	EnVision	1040142	10000
25	Patient 25	BM	EnVision	1040142	10000
27	patient 27	BM	EnVision	1040142	10000
28	patient 28	BM	EnVision	1040142	3676
29	patient 29	BM	EnVision	1040142	8000
30	Patient 30	Blood	EnVision	1040142	10000
30	Patient 30_relapse	Blood	EnVision	1040142	10000
31	Patient 31	Blood	EnVision	1040142	10000
32	Patient 32	BM	EnVision	1040142	10000
33	Patient 33	BM	EnVision	1040142	10000
33	Patient 33_relapse	BM	EnVision	1040142	5000
34	Patient 34	BM	EnVision	1040142	10000
34	Patient 34_relapse	BM	VictorX	20302104	
35	Patient 35	Blood	EnVision	1040142	2823
36	Patient 36	BM	EnVision	1040142	10000
37	Patient 37	BM	EnVision	1040142	2554
38	Patient 38	BM	EnVision	1040142	2382
4	Patient 4	BM	EnVision	1040142	8500
40	Patient 40	Blood	EnVision	1040142	10000
42	Patient 42	BM	EnVision	1040142	10000
42	Patient 42_relapse	BM	EnVision	1040142	10000
43	Patient 43	BM	EnVision	1040142	661
44	Patient 44	BM	EnVision	1040142	10000

47	Patient 47	BM	EnVision	1040142	4200
51	Patient 51	BM	EnVision	1040142	10000
52	Patient 52	BM	EnVision	1040142	10000
54	Patient 54	BM	EnVision	1040142	10000
57	Patient 57	Blood	EnVision	1040142	10000
58	Patient 58	BM	EnVision	1040142	10000
60	Patient 60	BM	EnVision	1040142	1352
62	Patient 62	Blood	EnVision	1040142	10000
64	Patient 64	BM	EnVision	1040142	10000
65	Patient 65	BM	EnVision	1040142	5000
66	Patient 66	BM	EnVision	1040142	1000
7	Patient 7	BM	EnVision	1040142	10000
70	Patient 70	Blood	EnVision	1040142	10000
71	Patient 71	BM	EnVision	1040142	10000
71	Patient 71_relapse	BM	VictorX	20302104	
73	Patient 73	BM	EnVision	1040142	750
74	Patient 74	BM	EnVision	1040142	10000
75	Patient 75	BM	EnVision	1040142	795
76	Patient 76	BM	EnVision	1040142	1205
76	Patient 76_relapse	BM	VictorX	20302104	
78	Patient 78	BM	EnVision	1040142	10000
79	Patient 79	BM	EnVision	1040142	10000
80	Patient 80	BM	VictorX	20302104	1000
82	Patient 82	BM	VictorX	20302104	1500
83	Patient 83	BM	VictorX	20302104	3911
84	Patient 84	BM	VictorX	20302104	2000
85	Patient 85	BM	VictorX	20302104	533
88	Patient 88	BM	VictorX	20302104	10000
88	Patient 88_relapse	BM	VictorX	20302104	
89	Patient 89	BM	VictorX	20302104	10000
9	Patient 9	BM	EnVision	1020030	10000
92	Patient 92	Blood	VictorX	20302104	10000
93	Patient 93	BM	VictorX	20302104	10000
95	Patient 95	Blood	VictorX	20302104	10000
98	Patient 98	BM	VictorX	20302104	10000
