# Supplementary material for: Clinical forecasting of acute myeloid leukemia using ex vivo drug-sensitivity profiling
Source: Cell Rep Methods. 2023 Dec 7;3(12):100654. doi: 10.1016/j.crmeth.2023.100654 (PMC10753296; doi:10.1016/j.crmeth.2023.100654)
Supplement: Document S2. Article plus supplemental information [file mmc2.pdf]

Cell Reports
Methods

Article

Clinical forecasting of acute myeloid leukemia using *ex vivo* drug-sensitivity profiling

Graphical abstract

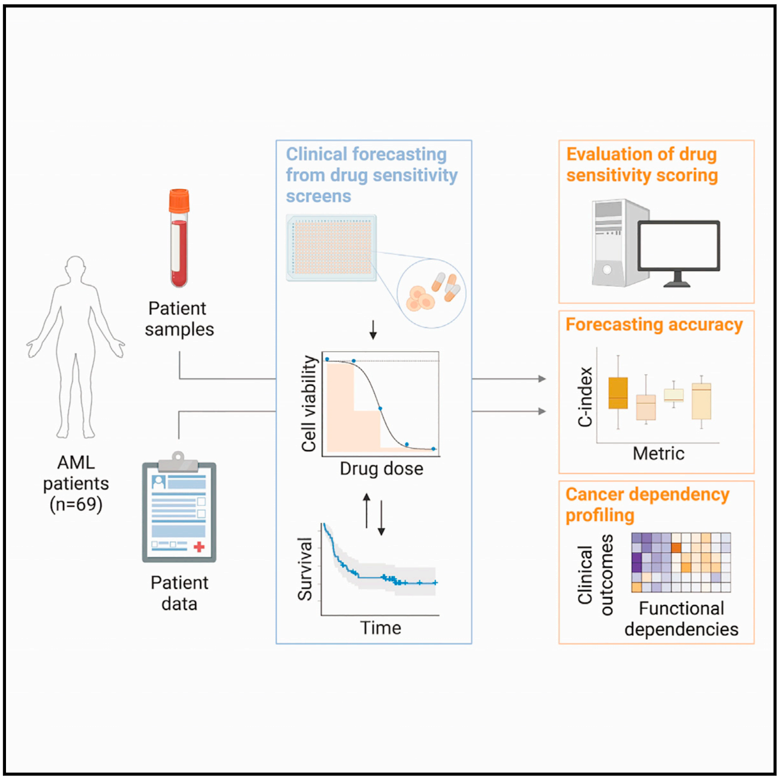

Authors

Aram N. Andersen, Andrea M. Brodersen, Pilar Ayuda-Durán, ..., Yngvar Fløisand, Sigrid S. Skånland, Jorrit M. Enserink

Correspondence

a.n.andersen@ibv.uio.no (A.N.A.),
jorrit.enserink@ibv.uio.no (J.M.E.)

In brief

Predicting the response to chemotherapy for treatment of AML is a major challenge. Mutation analysis and gene panel diagnostics are routinely used to predict treatment outcome, but the predictive power of these methods is limited. Here, Andersen et al. demonstrate that *ex vivo* drug profiling can assess risk of individual AML patients and may guide clinical decision-making.

Highlights

- Systematic evaluation of computational methods to analyze *ex vivo* drug-profiling data
- Identification of a drug fingerprint that forecasts chemotherapy outcome in patients
- Drug profiling predicts treatment outcome at least as well as current AML biomarkers

Andersen et al., 2023, Cell Reports Methods *3*, 100654
December 18, 2023 © 2023 The Author(s).
https://doi.org/10.1016/j.crmeth.2023.100654

CellPress

Cell Reports Methods

CellPress
OPEN ACCESS

Article

Clinical forecasting of acute myeloid leukemia using *ex vivo* drug-sensitivity profiling

Aram N. Andersen,[1,2,3,8,*] Andrea M. Brodersen,[1,2,3,8] Pilar Ayuda-Durán,[1,2,8] Laure Piechaczyk,[1,2] Dagim Shiferaw Tadele,[1,2,7] Lizet Baken,[1,2] Julia Fredriksen,[1,2,4] Mia Stoksflod,[4] Andrea Lenartova,[4] Yngvar Fløisand,[2] Sigrid S. Skånland,[5,6] and Jorrit M. Enserink[1,2,3,9,*]

[1]Department of Molecular Cell Biology, Institute for Cancer Research, Oslo University Hospital, Montebello, 0379 Oslo, Norway
[2]Centre for Cancer Cell Reprogramming, Institute of Clinical Medicine, Faculty of Medicine, University of Oslo, Blindern, 0318 Oslo, Norway
[3]Section for Biochemistry and Molecular Biology, Faculty of Mathematics and Natural Sciences, University of Oslo, Blindernveien 31, 0371 Oslo, Norway
[4]Department of Haematology, Oslo University Hospital, 0372 Oslo, Norway
[5]Department of Cancer Immunology, Institute for Cancer Research, Oslo University Hospital, Montebello, 0379 Oslo, Norway
[6]K.G. Jebsen Centre for B Cell Malignancies, Institute of Clinical Medicine, University of Oslo, 0372 Oslo, Norway
[7]Present address: Cleveland Clinic, Department of Translational Hematology and Oncology Research, Lerner Research Institute, Cleveland, OH 44195, USA
[8]These authors contributed equally
[9]Lead contact
*Correspondence: a.n.andersen@ibv.uio.no (A.N.A.), jorrit.enserink@ibv.uio.no (J.M.E.)
https://doi.org/10.1016/j.crmeth.2023.100654

MOTIVATION *Ex vivo* drug-sensitivity screening may be used to assess treatment options and predict treatment response. However, the effectiveness of this approach relies on the preservation of clinical cancer characteristics in *ex vivo* cell systems, and systematic data on accurate representation of drug-response profiles are lacking. Here, we used forecasting of clinical outcomes as a standard for exploring optimal scoring of drug sensitivities and management of noise and technical confounders. We show that *ex vivo* drug-sensitivity profiles robustly predict AML patient survival and provide valuable insights about treatment response and disease progression.

SUMMARY

Current treatment selection for acute myeloid leukemia (AML) patients depends on risk stratification based on cytogenetic and genomic markers. However, the forecasting accuracy of treatment response remains modest, with most patients receiving intensive chemotherapy. Recently, *ex vivo* drug screening has gained traction in personalized treatment selection and as a tool for mapping patient groups based on relevant cancer dependencies. Here, we systematically evaluated the use of drug sensitivity profiling for predicting patient survival and clinical response to chemotherapy in a cohort of AML patients. We compared computational methodologies for scoring drug efficacy and characterized tools to counter noise and batch-related confounders pervasive in high-throughput drug testing. We show that *ex vivo* drug sensitivity profiling is a robust and versatile approach to patient prognostics that comprehensively maps functional signatures of treatment response and disease progression. In conclusion, *ex vivo* drug profiling can assess risk for individual AML patients and may guide clinical decision-making.

INTRODUCTION

Acute myeloid leukemia (AML) is a heterogeneous cancer where the clonal expansion of myeloid progenitor cells (blasts) in the bone marrow and peripheral blood interfere with healthy hematopoiesis, resulting in immunodeficiency, thrombocytopenia, and anemia.[1] The current 5-year survival rate of patients over 60 years of age is estimated to be 10%–15%.[2] Most treatment-eligible individuals receive standard induction chemotherapy, which consists of a 3-day treatment with either 60 mg/m^2 daunorubicin or 10–12 mg/m^2 idarubicin and 100–200 mg/m^2 cytarabine (Ara-C) intravenously for 7 days.[3] Survival rates of older patients have not substantially improved over the past decades, underscoring

 © 2023 The Author(s).
This is an open access article under the CC BY license (http://creativecommons.org/licenses/by/4.0/).

the need for better clinical assessment and more accurate prognostic approaches.

Current risk stratification methods such as the European LeukemiaNet (ELN) guidelines for AML stratification are mainly based on parameters such as molecular pathology and genomic alterations.[4] However, a substantial number of patients remain difficult to stratify, such as cytogenetically normal AML, and approximately 50% of patients are stratified as intermediate-risk patients for whom selection of the appropriate treatment regimen remains a major challenge.[4–6] Various non-genomic methods have been developed for identification of clinically and biologically relevant molecular subtypes for risk stratification, such as flow and mass cytometry, transcriptomics, and proteomics,[7–12] although clinical implementation of these methods has generally been slow.

Recently, *ex vivo* drug sensitivity profiling has been used as a precision medicine approach to identify potential compounds that may be repurposed for the treatment of various types of cancer, including AML.[13–20] Here, leukemic cells derived from bone marrow aspirates or peripheral blood are incubated in the presence of various drugs at different concentrations. Drug responses are then quantified, and overall drug efficacy is scored from a dose-response relation. This approach allows for screening of a high number of compounds in just 3 days and has identified potential alternative treatment avenues for AML.[13–19]

The validity of drug sensitivity profiling requires that clinically relevant characteristics are conserved in *ex vivo* analyses; i.e., drug responses should reflect cancer dependencies, and drug sensitivities should be measurable reliably despite potential technical confounders and noise. Several quality control practices have been developed to ensure the fidelity of high-throughput drug testing.[21–24] A common procedure is to use model curve fitting to de-noise dose-response data and summarize the drug sensitivities with the half-maximal effective concentration (EC_{50}) or the area under the curve (AUC).[25,26] Development of dose-range standardized function integrals based on the Hill equation, such as the drug sensitivity score (DSS), have improved the reproducibility of drug sensitivity profiles across independent studies.[27–29] However, curve fitting of incomplete or non-sigmoidal drug responses remains a challenge for large-scale drug testing, and single output metrics from Hill models tend to yield incomplete information about the biological variability of a given drug response.[25,30] Therefore, streamlined analysis pipelines typically offer multiple alternative dose-response evaluation and error-reporting tools.[23,31]

While *ex vivo* drug screening of cancer cells using clinically relevant chemotherapeutics has been shown to predict treatment outcome, a systematic evaluation of the methodology and clinical use of drug sensitivity profiling in more expansive drug sets has not been done.[32–35] In this study, we evaluated the clinical information available in *ex vivo* drug sensitivity profiles within a cohort of 69 AML patients by using several statistical techniques and machine learning routines. Regularized regression was used to evaluate the clinical forecasting potential and risk-interpretability from *ex vivo* drug sensitivity profiles. We show that drug sensitivity profiling can be used as a tool for clinical forecasting and decision-making.

RESULTS

Study approach, quality control, and comparison of metrics

To evaluate the clinical utility of *ex vivo* drug sensitivity profiling in AML, we systematically performed drug screens on bone marrow or peripheral blood samples from patients that were obtained at the time of diagnosis (Figure 1A and Table S1). For high-throughput curve fitting and multiparametric scoring of drug sensitivities, we used the Breeze pipeline, which performs Hill curve fitting on inhibitory responses to compute several drug sensitivity metrics, such as the EC_{50}, toxicity EC_{50} (TEC_{50}), and DSS1, DSS2, and DSS3.[23,27] Here, DSS1 is the relative integral for Hill curves that exceed 10% inhibition, DSS2 adjusts DSS1 to normalize for strongly toxic responses at high concentrations, and DSS3 adjusts DSS2 to give weight to responses with high dose sensitivity (Figure 1B, see STAR Methods). We also used Breeze to compute the AUC under a locally estimated scatterplot smoothing (LOESS) curve fit (loess-AUC), as a more flexible model-free alternative to the Hill equation. In addition to these metrics, we calculated a raw AUC based on a stepwise normalized rectangular area under the relative viability dose response along a logarithmic concentration scale (rAUC; Figure 1B). Since the distributions of relative viabilities are positively skewed, we also performed a negative log_2-transformation of the rAUCs, resulting in a weighted-average log_2 fold change in cell viability (rAUC-log_2). This step was equivalently performed on the loess-AUCs.

To obtain a measure of the quality of the drug screens we computed plate-wise Z′-factors and analyzed drug sensitivity profile correlations between patients to detect potential outliers (Figures S1A–S1D). Although some patient-specific plates had a suboptimal Z′-factor (Figure S1A), all negative plate controls (DMSO) were at least two standard deviations above the positive plate controls (BzCl, Figure S1B), and we did not observe any consistent association between plate control noise and patient profile correlations (Figure S1C). However, we did find substantial differences in profile correlations for different metrics, with DSS1-3, rAUC-log_2, and loess-AUC-log_2 yielding the best inter-patient correlations (median Pearson correlation coefficient [PCC] over 75%) and TEC_{50} and EC_{50} giving the worst (Figures S1C and S1D). Furthermore, intra-patient profile correlations between treatment-naive and relapsed samples were higher than inter-patient correlations (Figure S1D). Comparison of the different metrics revealed that low-confidence Hill curve fits reported by Breeze tend to be associated with low-sensitivity dose-responses ($EC_{50} > 10^{-7}$ μM) (Figure S1E), as well as with responses yielding lower correspondence between different AUC metrics. Despite this, the overall correlation between the different metrics was high, indicating that inferences made from the drug screen dataset were sufficiently robust for these analysis methods.

Assessing the predictivity of drug sensitivity metrics

Median survival in the patient cohort was 1,177 days (Figure 1C). To assess *ex vivo* drug sensitivity profiling in predicting patient survival, we compared several regularized Cox models trained on the different drug sensitivity metrics (Figure 1D). Predictive

CellPress
OPEN ACCESS

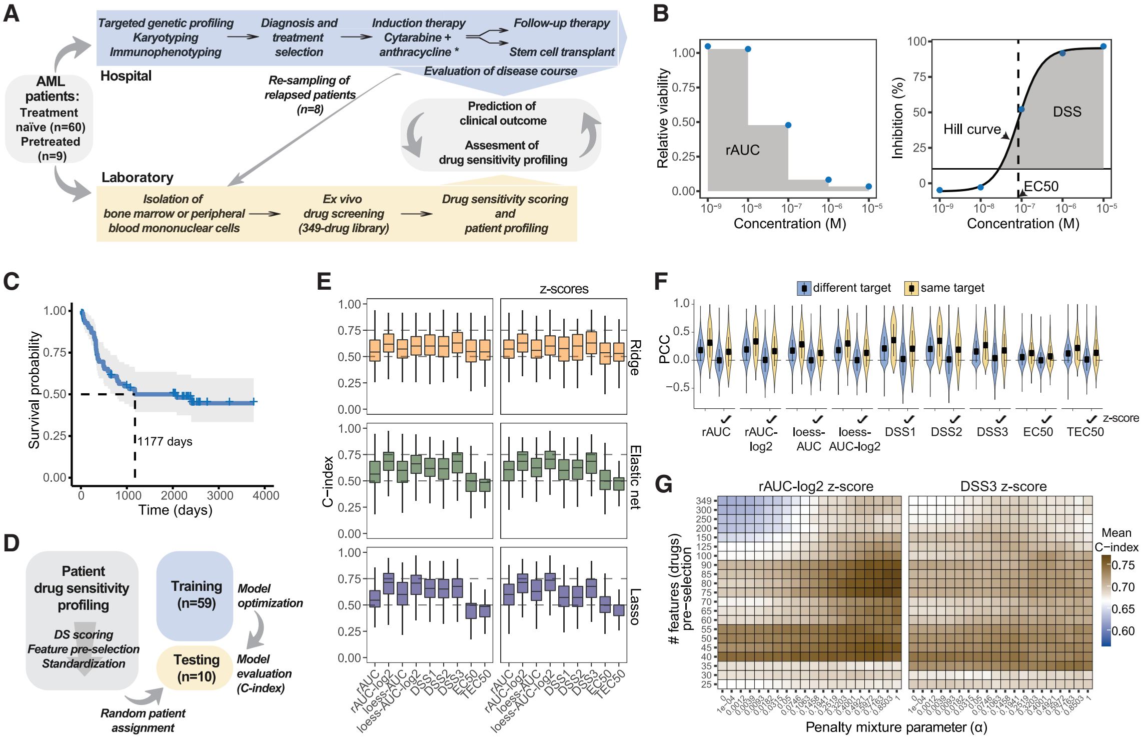

Figure 1. Survival prediction from *ex vivo* drug sensitivity profiles

(A) Study overview and workflow.

(B) Drug sensitivity metric computation. The gray areas indicate how the rAUC and DSS are calculated.

(C) Survival curve of the study cohort. Ticks indicate censoring.

(D) Machine learning routine for testing survival prediction from different drug sensitivity metrics and data processing operations.

(E) C-index results (200 tests) for Cox models trained on different drug sensitivity metrics (left) or drug sensitivity *Z* scores (right).

(F) Pearson correlation coefficients of drug sensitivities between drug pairs having the same or different target.

(G) Mean test C-index results (50 tests) for Cox models trained on rAUC-log_2 or DSS3 *Z* scores with various penalty mixture parameters (α) and feature pre-selection thresholds based on rAUC standard deviations.

accuracy was assessed using a C-index over multiple randomized assignments of patient test data (Figure 1D). We also tested different regularization types, as Ridge and Elastic net regression tend to perform better than Lasso when there are grouped correlations between features (which is expected for drugs with similar modes of action).[36] AUC-based metrics outperformed EC_{50} and TEC_{50} in predicting patient survival regardless of regularization technique (Figure 1E). Interestingly, the loess-AUC, which had a strong linear correlation with the rAUC (Figure S1E), improved the survival prediction for Elastic net and Lasso models, suggesting that de-noising dose-response data using a LOESS curve fit has a beneficial effect (Figure 1E, left). This was also the case for the DSS metrics for all three model types, with DSS3 outperforming DSS1 and DSS2 and resulting in lower overfitting (Figures 1E and S1H). Strikingly, log-transforming the AUCs resulted in a substantial improvement in prediction accuracy, performing at least as good as DSS3 (Figure 1E, left), indicating that distributional skewness in relative viabilities has a negative impact on appropriate drug sensitivity scoring.

To counter potential batch effects, we standardized the drug sensitivity distribution for each patient (Figure S1F). This technique transforms the drug sensitivity metrics to a respective *Z* score, assuming that the major differences in patient distributions mainly reflect technical influences and that only changes in the relative magnitude of specific drug sensitivities are relevant. This operation further improved the prediction accuracy for the AUC-based metrics, with rAUC-log_2 and loess-AUC-log_2 yielding the best performances with respective median C-index scores of 71% and 74% using Lasso (Figure 1E, right). In contrast, patient standardization of the DSS metrics had little effect on the predictivity and caused a slight reduction in performance for models trained with the Lasso penalty (Figure 1E). To better understand these effects, we measured the inter-drug profile correlations, which revealed library-wide multicollinearity that was removed when standardizing the metrics but maintained to a certain degree for drug pairs targeting similar proteins (Figure 1F). Here, DSS1 and DSS2 showed a slightly stronger correlation than DSS3 and other AUC metrics.

Scaling the drug distributions (Figure S1F) to equalize the penalty of drugs with differences in drug sensitivity variance caused a reduction in prediction accuracy using most metrics except for EC_{50} and TEC_{50}, where it caused an improvement (Figure S1I). This suggests that drugs generating weak and noisy responses have a negative impact on the model performances. Given the large number of drugs relative to the number of patients, we

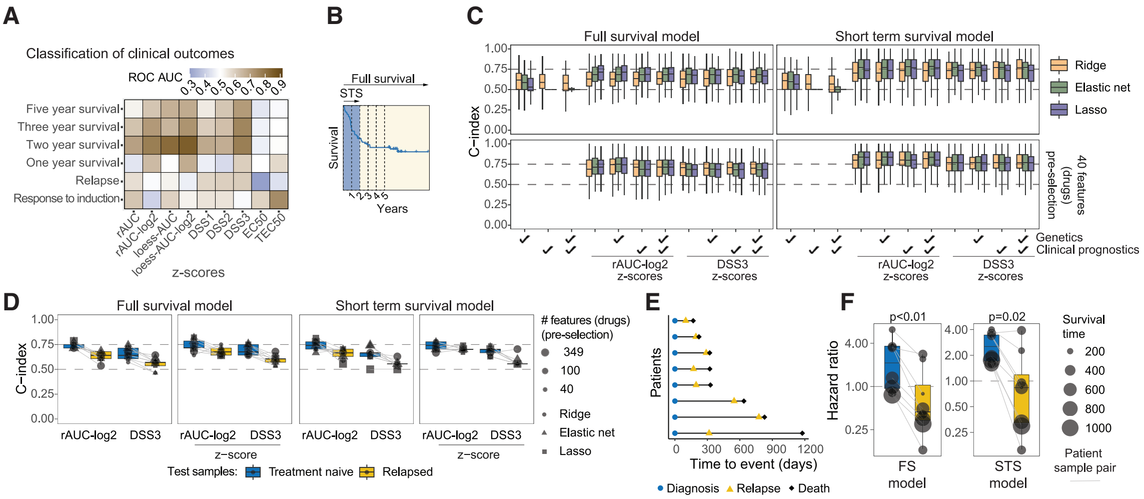

Figure 2. Versatility of drug sensitivity profiling for clinical outcome predictions

(A) Average ROC-AUC for classification of various binarized clinical outcomes using different drug sensitivity metrics. The values are averaged over all the ROC-AUC scores from models with different penalties and pre-selection thresholds in Figure S2A.

(B) The first 2 years after diagnosis characterized by a 40% drop in survival was defined as an initial phase for short-term survival (STS) modeling using Cox regression. The STS models were compared with the full survival (FS) models that were trained on data from the entire study interval.

(C) Test C-index results (200 tests) for Cox models trained on different dataset compositions based on clinical feature sets and rAUC-log$_2$ or DSS3 *Z* scores. The lower panels represent pre-selection of 40 features based on rAUC standard deviations, and the right panels represent prediction results from short-term survival modeling.

(D) Test C-index on samples from treatment-naive and relapsed patients, for different models trained on rAUC-log$_2$ or DSS3, or their corresponding *Z* scores. The two right panels represent prediction results from short-term survival modeling.

(E) Overview of the time of relapse in relation to the time of death for the eight patients in the relapse cohort.

(F) Predicted hazard ratio (relative to population median) on samples from treatment-naive and relapsed patients for Elastic net models trained on rAUC-log$_2$ using 100 pre-selected features. Right panel represents short-term survival modeling. p values were computed using a paired Wilcoxon test.

therefore tested the effect of pre-selecting features ranked by their standard deviations (to remove weak drug responses) as well as a greater range of Elastic net penalties (Figure S1G). These operations, in particular feature pre-selection, improved the survival predictions further, with rAUC-log$_2$ yielding C-index averages over 77% for several Elastic net models (Figures 1G and S1J). Altogether, these results show that there is high prognostic value in patients' *ex vivo* drug sensitivity profiles.

Versatility of clinical forecasting and integration with AML biomarkers

To evaluate the prognostic versatility and robustness of *ex vivo* drug profiling, we also tested the forecasting potential of other clinical outcomes, including survival status after different periods. We found that the accuracy of AUC-based metrics at predicting the initial response to induction therapy (as measured by the presence of persistent leukemia in the patients), long-term survival, or probability of relapse was limited (Figures 2A and S2A). In contrast, the survival rates during the first 2 years after diagnosis, which is characterized by a sharp reduction in survival (Figure 2B), were predicted by AUC-based metrics with very high accuracy (Figures 2A and S2A). Furthermore, while rAUC-log$_2$ and loess-AUC-log$_2$ excelled over DSS metrics at short-term survival prediction, DSS3 robustly performed better than all other metrics in predicting long-term survival (Figures 2A and S2A). These results show that drug sensitivity profiles predict well the initial treatment phase but also have potential for long-term survival forecasting.

We next compared the effectiveness of *ex vivo* drug sensitivity profiling in predicting survival to the predictiveness of other AML patient data, such as genetic features and/or prognostic clinical features that included ELN risk stratifications, age, and sex. We also performed the analysis on 2-year truncated short-term survival data to evaluate the performance on the initial treatment response phase.

As expected, due to coverage sparsity, the Ridge models performed better on the genetic and clinical feature sets (Figure 2C). The genetic feature set provided a prediction with a median C-index of 59% overall and 60% short term, which is close to what has been reported previously using much larger cohorts.[37] Combining the different clinical feature sets did not improve predictivity, most likely due to redundancy in information and low sample size (Figures 2C, S2B, and S2C). Interestingly, *ex vivo* drug screening performed at least as well as or better than the genetic or clinical feature sets at predicting treatment outcome (Figure 2C). Combining genetic or clinical feature sets with the entire drug dataset did not substantially improve predictivity, and combining genetic features with a reduced drug dataset using feature pre-selection only resulted in a slightly improved but non-significant combination effect (Figure 2C, left lower panel). Furthermore, the feature-reduced rAUC-log$_2$ dataset showed prediction accuracy of 83% for two-year survival (Figure 2C, right lower panel), which did not improve further when combined with the genetic or clinical feature sets. There were also no complementary effects between the datasets when testing

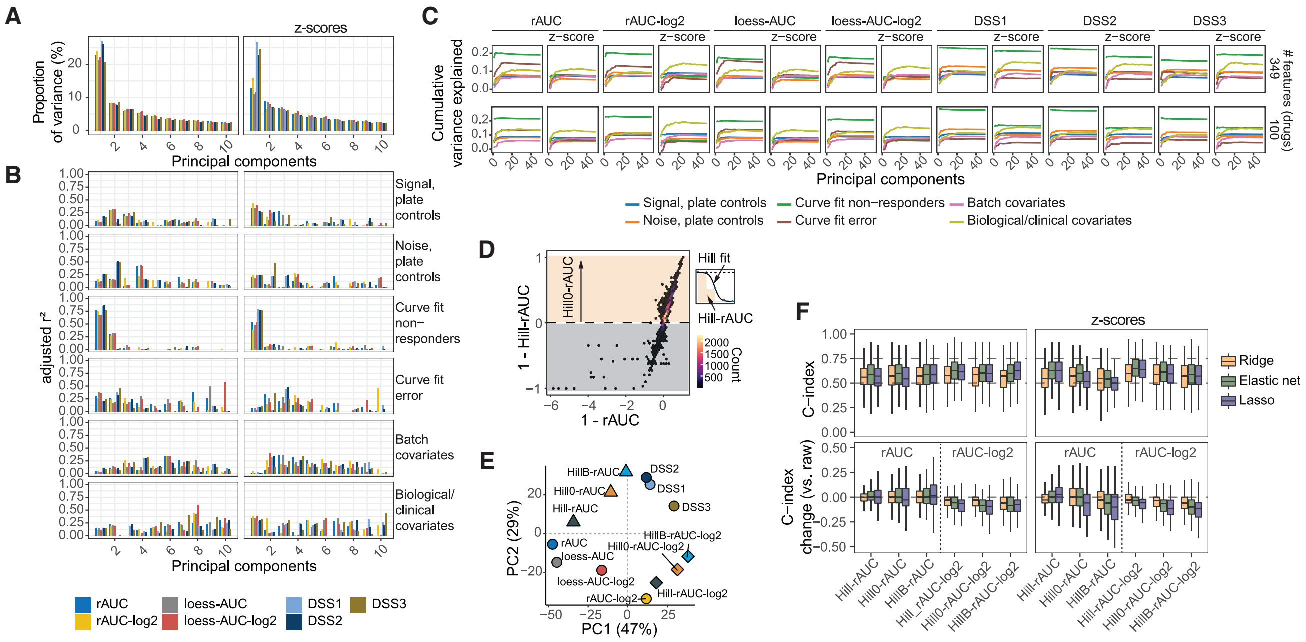

Figure 3. Exploring confounding factors in *ex vivo* drug sensitivity profiles

(A) Percent variance explained by principal components for different drug sensitivity metrics and *Z* scores.

(B) Principal component variance explained by different patient sample characteristics for different drug sensitivity metrics and *Z* scores (to the right).

(C) Cumulative variance explained by different patient sample characteristics, for different drug sensitivity metrics and *Z* scores, with or without feature pre-selection based on rAUC standard deviations to remove weak and noisy drug responses.

(D) Correlation between rAUC and Hill-rAUC estimated from a stepwise normalized rectangular area under the relative viability dose-response predicted by a Hill curve fit. Hill0-rAUC represents only inhibitory responses by setting all non-inhibitory values to zero.

(E) PCA plot of the first two principal components indicating the similarity between the different AUC- and DSS-based drug sensitivity metrics compared in the study. The direction (sign) of all the metrics were harmonized, and the PCA was done without scaling.

(F) Test C-index (200 tests) for Cox models trained on Hill-based rAUC or rAUC-log$_2$ scores or corresponding Z scores (upper panels) and their computed C-index change from the non-curve fit raw rAUC or rAUC-log$_2$ counterparts (lower panels).

for other clinical outcomes (Figure S2D). This marked a 23% improvement over the prediction using genetic features alone.

Since drug sensitivities may change during the progression of a disease and in response to treatment, we evaluated the survival prediction on relapsed patient samples and compared to their treatment-naive counterparts. Here, the survival models were trained by completely excluding data from both treatment-naive and relapsed samples for the patients that were tested. Interestingly, despite a major reduction in predictivity, standardizing the rAUC-log$_2$ scores, but not DSS3, was able to nearly rescue the performance on relapsed samples (Figures 2D and 2E), indicating that the relative risk prediction between patients sampled from the same disease stage is preserved. Surprisingly, this was despite a significant reduction in predicted patient hazard for relapsed samples overall (Figures 2F and S2E). This suggests that whereas the cancer dependencies relevant for initial survival prognostics of high-risk patients evolve during the course of therapy, the relative patient identity characteristics remain preserved.

Removal of confounding factors in *ex vivo* drug sensitivity profiles

Due to the major differences in clinical forecasting performance between the different drug sensitivity metrics, we performed principal component analysis (PCA; Figure S3A) to identify potential confounding factors that may cause variability in the datasets, such as artifacts associated with curve fitting, batch covariates, instrument and sample type, and biological/clinical covariates based on genetic and diagnostic phenotypes.

The first principal component identified over 20% of the total variance (Figure 3A, left) and was strongly associated with curve fit non-responders (>75%, Figure 3B, left). Moreover, noise in plate controls and curve fit error had strong associations with the first two to four components of DSS1, DSS2, and DSS3, whereas the rAUCs and loess-AUCs were affected across multiple components throughout the dataset (Figure 3B, left). Strikingly, for any drug sensitivity metric, the first few components had very weak association with biological/clinical covariates, suggesting that the major cause of variability in drug sensitivities stems from patient response averages, noise, and potential curve fit issues. These effects were rectified to some extent by standardizing the drug sensitivities per patient and removing weak and noisy responses using feature pre-selection, particularly for rAUC and rAUC-log$_2$ (Figures 3A–3C and S3B). These operations also increased the variance explained by biological/clinical covariates, while decreasing the effect of other sample characteristics, such as number of patient non-responders, curve fit error, and noise in the plate controls (Figures 3B and 3C). For the rAUCs and loess-AUCs metrics, these results

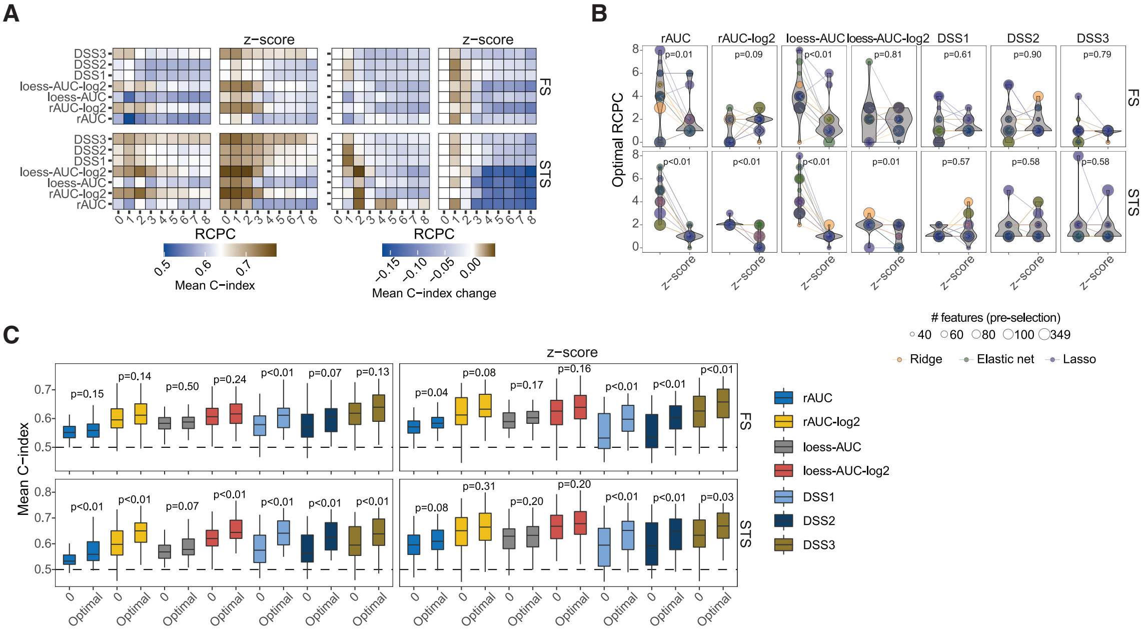

Figure 4. Effect of removing confounding principal components on survival prediction

(A) Mean test C-index (left four panels) or C-index change (right four panels) for different survival models trained on different drug sensitivity metrics or *Z* scores with different numbers of principal components removed. The C-index changes are computed from the respective reference datasets (RCPC = 0). The values are averaged over all the C-index test scores from models with different penalties and pre-selection thresholds in Figure S4A. The lower panels represent results for short-term survival (STS) models.

(B) Number of components removed to achieve the highest mean test C-index for different drug sensitivity metrics or *Z* scores shown in Figure S4A. The lower panels represent results for short-term survival (STS) models. p values were computed using a paired Wilcoxon test.

(C) Mean test C-index results (50 tests) for Lasso survival models comparing zero or the optimal number of components removed for 50 datasets generated under weighted random sampling of features for different drug sensitivity metrics or *Z* scores (shown in Figure S4C). The lower panels represent results for short-term survival (STS) models. p values were computed using a paired Wilcoxon test.

highlighted their sensitivity to noise (represented by curve fit error), which was strongly reduced when the metrics were standardized (Figure 3C).

Interestingly, considering the strongest drug responses (with feature pre-selection) improved the relative variance explained by biological/clinical covariates only for rAUC-log_2 and loess-AUC-log_2 but not the DSS metrics (Figure 3C). To investigate whether this was related to loss of information from curve fitting or removing non-inhibitory responses, we performed Hill curve fitting and computed AUCs from the rectangular area under the predicted dose-responses (Hill-rAUC; Figures 3D and S3C–S3E). The Hill-rAUC was further adjusted for non-inhibitory responses (Hill0-rAUC) for a more direct comparison with the inhibition-only curve fits from Breeze protocol (HillB-rAUC) and the DSS metrics (Figure 3E). All of these metrics were also log_2-transformed to enable comparison with the corresponding non-curve fit AUCs. These operations indicated that there was little benefit on survival prediction from curve fitting alone and a further negative influence of zero-inflating non-inhibitory dose-responses (Figure 3F). Moreover, in particular, removing variation from non-inhibitory responses replicated many of the covariance characteristics observed for the DSS metrics using PCA (Figure S3G).

To test whether the dominant covariances in the data represented confounding factors that interfered with accurate prediction of patient survival, we performed a removal of confounding principal components (RCPC) procedure.[38] For most drug sensitivity metrics, removing at least one or two principal components improved prediction accuracy (Figures 4A, 4B, S4A, and S4B), which was more profound for short-term survival predictions. Interestingly, both log_2-transformation and standardization appeared to negate the beneficial effect of RCPC on rAUC and loess-AUC (Figures 4B, 4C, and S4B–S4E), which compared to DSS metrics had a sharper decline in prediction performance when removing a greater number of principal components (Figures 4A and S4D), indicating loss of valuable information about patient survival. Finally, standardizing the rAUCs and loess-AUCs resulted in a significant decrease in the number of component subtractions required for achieving optimal performance for short-term survival (Figures 4B and S4E). These results indicate that processed *ex vivo* drug screening data can contain consequential confounders that may bias the dose-response curve fit and drug sensitivity scoring, and that standardization or RCPC can be used as a straightforward and reliable method for diagnosing and de-confounding the data.

Drug sensitivity risk associations

Statistical analysis of the library-wide survival associations revealed that different models showed overall good correlation in their learned coefficients (Figures 5A, 5B, S5A, and S5B), where

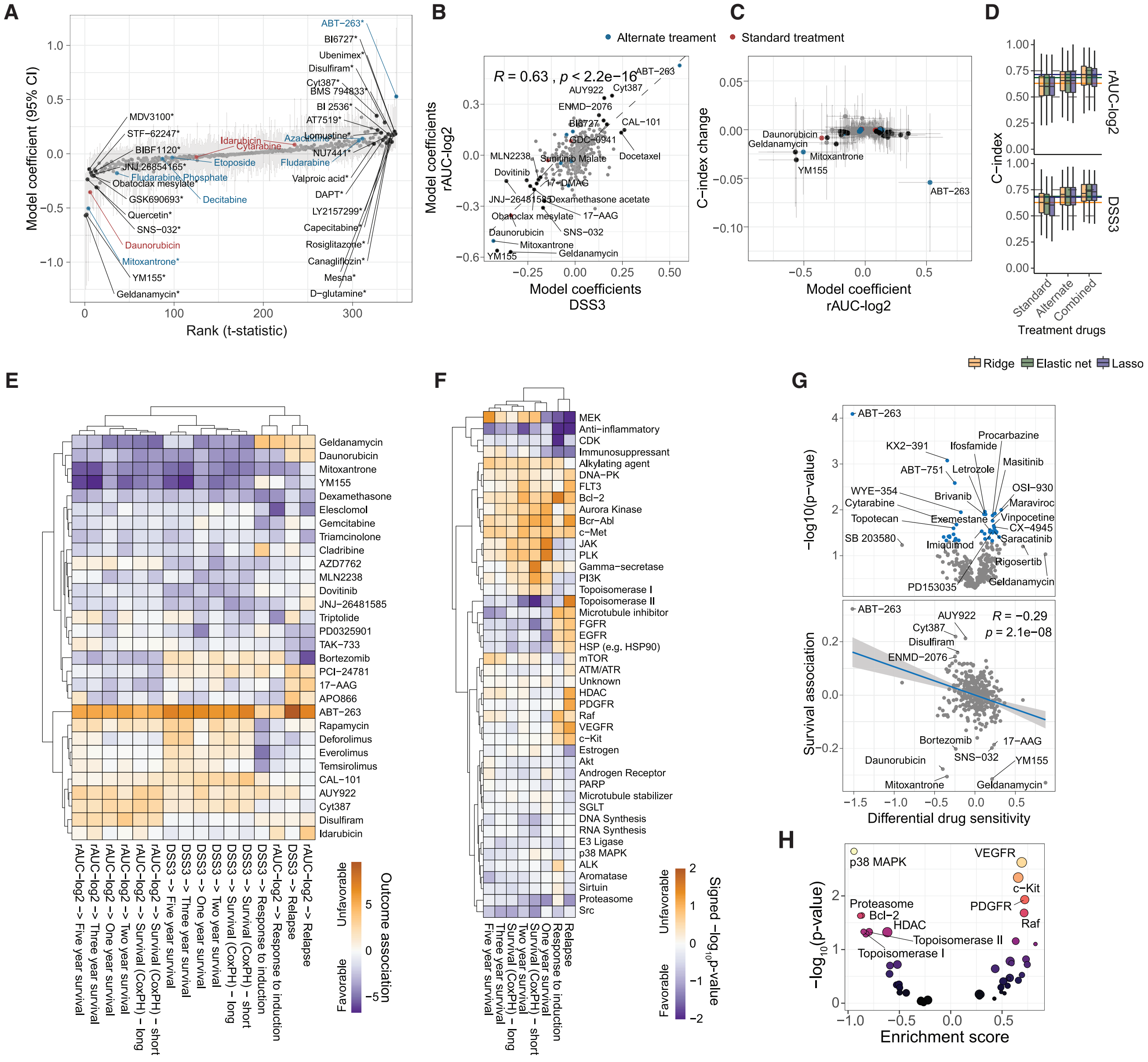

Figure 5. Clinical associations of *ex vivo* drug sensitivities

(A) Bootstrapped ridge survival coefficients representing risk association of AUC-log_2 Z scores for 349 drugs. Gray vertical bars indicate 95% confidence intervals for each individual compound. Significance (*) was determined when 95% of the bootstrapped coefficients did not include or cross zero.

(B) Correlation between estimated ridge survival coefficients for rAUC-log_2 Z scores and DSS3 Z scores. The drugs with the strongest coefficients in both models are labeled.

(C) Association between mean survival coefficients and mean test C-index change (50 tests) in response to drug withdrawal using ridge regression on rAUC-log_2 Z scores. Horizontal and vertical bars indicate standard deviations. Selected outliers are labeled. Standard or alternative AML treatment drugs are color-coded in (A)–(C). Significant drugs from (A) are color-coded as black in (C).

(D) C-index results (200 tests) for Cox models trained on rAUC-log_2 and DSS3 Z scores for *ex vivo* treatment drug responses. The horizontal lines indicate the respective C-index test medians for the full models in Figure 1E.

(E) Clustering of normalized ridge coefficients from models trained against different clinical outcomes using rAUC-log_2 and DSS3 Z scores.

(F) Clustering of drug target enrichment p values based on directional drug-set enrichment on ranked coefficients from ridge models trained against different clinical outcomes using rAUC-log_2 Z scores.

(G) Differential drug sensitivity (for rAUC-log_2 Z scores) between samples from relapsed and treatment-naive patients, with paired t test p values (upper) and association with ridge survival coefficients (lower). Blue dots indicate significance. The Pearson correlation coefficient (R) and p-values are indiciated in the lower panel.

(H) Drug target association with differential sensitivity, using directional drug-set enrichment on differential drug sensitivities from (G).

the strongest survival associations also corresponded to high contributions to a model's prediction accuracy (Figures 5C and S5C). Given that many compounds in the library have overlapping targets, there was substantial redundancy in single-drug withdrawals with respect to model performance (Figure 5C). In-depth investigation of specific drug responses that were significantly associated with survival revealed that *ex vivo* sensitivity to anthracyclines predicted a favorable outcome (Figure 5A). Daunorubicin, one of the major components of 7 + 3 chemotherapy, had one of the most significant associations with survival, while idarubicin also had a favorable (albeit statistically non-significant) survival association. Epirubicin, another anthracycline used in treatment of several types of cancers, and the anthracycline analog mitoxantrone were also significantly associated with survival (Figure 5A). Interestingly, we found that *ex vivo* sensitivity to the Bcl-2/Bcl-XL/Bcl-w inhibitor ABT-263 (navitoclax) was strongly associated with increased risk (Figure 5A). ABT-263 is an experimental drug with a mechanism of action similar to the more selective Bcl-2 inhibitor venetoclax, which has been reported to significantly improve survival of AML patients who are ineligible for standard chemotherapy (please note that venetoclax had not been approved for AML therapy at the time of sample collection, which is why it was absent from the drug library).[39] Strikingly, models trained on sets of treatment drugs alone achieved similar survival forecasting performance as the full library, and combining standard and alternate treatment drugs had a complementary effect on prediction accuracy (Figures 5D and S5D). This shows that improved prognostic value from drug sensitivity profiling can be achieved by carefully selecting drug libraries with non-redundant information about key cancer dependencies.

Hierarchical clustering of learned risk associations against different clinical outcomes revealed interesting correlations (Figures 5E and S5E). For instance, sensitivity to the mTOR inhibitors rapamycin, everolimus, deforolimus, and temsirolimus was associated with initial positive response to induction therapy but also with increased risk of relapse and decreased long-term survival. This implies that dependence on mTOR may be an early prognostic marker for acquired resistance to chemotherapy. Similarly, whereas sensitivity to daunorubicin was most positively associated with short-term survival and response to induction therapy, it was also associated with increased risk for relapse (Figure 5E).

Enrichment analysis of drug targets associated with Cox model coefficients demonstrated a library-wide risk association of sensitivity to kinase inhibitors, including JAK and PI3K signaling (Figures 5F and S5F–S5H). On the other hand, relapse risk was associated with sensitivity to multiple receptor tyrosine kinase inhibitors, and sensitivity was significantly increased in relapsed samples (Figures 5F–5H and S5F–S5I). Conversely, sensitivity to topoisomerase II-targeting chemotherapeutics in treatment-naive samples, which was associated with a favorable short-term survival and increased risk of relapse, was decreased in samples from relapsed patients (Figures 5F–5H). Consistent with previous studies,[40,41] *ex vivo* sensitivity to agents that target anti-apoptotic proteins, particularly ABT-263, was strongly reduced in relapsed samples (Figure 5G), indicating an increased apoptotic threshold or development of cross-resistance. This was also accompanied with a library-wide negative correlation trend between differential relapse sensitivity and risk association in treatment-naive samples, where several prognostic drug markers for adverse response to standard treatment diminished in sensitivity after relapse (Figure 5G, lower panel). We conclude that *ex vivo* drug sensitivity profiles can serve as prognostic markers and may provide insight into relevant cancer dependencies and mechanisms of disease progression.

DISCUSSION

In this retrospective study, we used *ex vivo* drug sensitivity screening to predict the survival of AML patients subjected to standard chemotherapy. An important topic that has been the subject of much discussion is how to appropriately score drug sensitivities in large-scale drug screens. Although sigmoid-constrained curve fitting and response scoring is a valuable tool in large-scale drug screens, it is inherently sensitive to dose coverage in the dynamic range, and model parameters tend to be unreliable.[25,30,42] Consistent with this, we observed that the EC_{50} was inferior in predicting patient survival compared to various scoring metrics based on the average dose-response efficacy. To combat the noisy nature of *ex vivo* drug screens, a common approach is to only consider the area under inhibitory Hill-response curves that cross a certain threshold, exemplified by the DSS.[27] We also observed that this compression of drug response variability resulted in zero-inflated data with substantial loss of information. Some of this is likely due to bias associated with Hill curve fitting, introducing confounding covariation that could be countered by removing the leading principal components in the data.

An alternative approach to the DSS that worked well without or in combination with model-free curve fitting was log-transforming averages of the relative viabilities. This is a common re-scaling procedure that when used in this context effectively reduces the leverage of noisy and weak data points around one or above (non-inhibitory responses) and improves the separability of strongly inhibiting drug responses. For these drug sensitivity metrics, which retained the full range of drug response variation, patient standardization (*Z* scoring) generally improved representation of clinical information in the data even further. This operation removes patient variation in general levels of drug sensitivity, which is responsible for substantial drug-wise correlations (multicollinearity) as observed by others.[43,44] This property has previously been linked to biological differences in gene expression and where conditioning on this feature can improve the inferences between datasets.[43,44] Here, using RCPC to remove this feature completely improved short-term survival forecasting, indicating that it served as a negative confounder for utility of specific drug sensitivity variations. Moreover, while the burden of confounding factors may vary between datasets, the use of PCA to investigate which sample features dominate the leading components can serve as a valuable tool to determine the approximate number of components to remove.[38] This can further be used in conjunction with other batch correcting methods to harmonize data from different centers.[45]

In our study, we used a drug library that not only includes standard chemotherapeutics but also a wide variety of other

CellPress
OPEN ACCESS

compounds, and we identified informative "drug sensitivity fingerprints" that may not only help guide treatment choice but that can also provide valuable prognostic information. Importantly, merging the data from drug sensitivity profiling with information from genetic biomarkers did not further improve the predictivity of drug profiling alone. This suggests that predictive information in genetic profiles is already captured by *ex vivo* drug sensitivities and indicates that *ex vivo* drug profiling can be further developed into a prognostic tool through rational design of focused drug libraries that fully cover cancer dependencies. Examples of drugs that should be included in such next-generation libraries for risk stratification are BCL2, mTOR, and HSP90 inhibitors, which, as already discussed above, are clearly associated with risk. Furthermore, we found that the five top-ranking drugs associated with high risk include three JAK2 inhibitors and one STAT3 inhibitor, which is consistent with previous findings that high JAK2/STAT3 activity causes resistance to chemotherapy.[46–48] We are currently analyzing data from other large-scale *ex vivo* drug screening efforts, such as the FIMM and BEAT-AML datasets,[49–51] which we expect will identify additional compounds associated with risk. Such analyses will not only be important for inter-center validation of data but also for development of effective harmonization methods.

Although progress has been made to update recommendations for AML risk stratification and treatment guidelines, prediction accuracies in large and heterogeneous cohorts remain modest.[4,37] One reason for this is that AML is in part driven by non-genetic alterations,[52,53] including epigenetic alterations and rewiring of metabolic pathways and signaling circuits, which are not easily detected by more conventional methods, such as genomics and transcriptomics. Functional *ex vivo* drug profiling may be better suited for identifying such cancer dependencies.[50,51] Thus, with further research, we envision that *ex vivo* drug profiling can be a useful tool in the clinical decision-making process to stratify patients into treatment groups.

We conclude that *ex vivo* drug profiling reveals cellular dependencies leading to chemoresistance and cancer progression, and it robustly predicts patient survival and response to induction therapy.

Limitations of the study

This study was conducted within a single center on a relatively homogeneous and small patient cohort. Because AML is genetically a heterogeneous disease, the mutation profiles were sparsely distributed in the patient population, which resulted in low statistical power when cross-examining genetic data with *ex vivo* drug sensitivities for patient risk group stratification and treatment response prediction. Thus, future studies on larger patient cohorts from multiple centers will be instructive for evaluating the clinical forecasting performance of drug sensitivity profiles across different age groups and sub-classes of AML. This may also allow for development of more flexible multimodal machine learning strategies that generalize across cohorts and can provide biologically informed risk categorization based on the optimal combination of genetic and drug response screen results.

In this study, we focused on variations of intensive chemotherapy given as a first-line treatment in the clinic and not on targeted therapies. The accuracy of clinical outcome predictions may vary depending on the selected therapy and the preservation of treatment-specific cancer determinants to *ex vivo* cell cultures. Given that several targeted therapies have entered the clinic, it will be important to perform similar studies on these patient cohorts. Ideally, this will identify a focused set of drugs that are highly predictive of the outcome of chemotherapy as well as targeted therapies. Using the systematic approaches that we have outlined here on more heterogeneous patient groups subjected to a wider array of treatment options may further refine the use of drug sensitivity screens as a tool for stratifying patients in appropriate treatment groups.

STAR★METHODS

Detailed methods are provided in the online version of this paper and include the following:

- KEY RESOURCES TABLE
- RESOURCE AVAILABILITY
  - Lead contact
  - Materials availability
  - Data and code availability
- EXPERIMENTAL MODEL AND STUDY PARTICIPANT DETAILS
  - Patient cohort
- METHOD DETAILS
  - *Ex vivo* drug screening
  - Dose-response analysis
  - Clinical data processing
  - Model training and cross-validation
  - Model testing
  - Analysis of confounding factors
  - Variable importance
  - Model coefficient clustering
  - Enrichment test
- QUANTIFICATION AND STATISTICAL ANALYSIS

SUPPLEMENTAL INFORMATION

Supplemental information can be found online at https://doi.org/10.1016/j.crmeth.2023.100654.

ACKNOWLEDGMENTS

We would like to thank all patients who participated in this study, as well as patient representatives from the Norwegian Blood Cancer Society (Blodkreftforeningen), members of the Enserink and Knævelsrud laboratories for fruitful discussions, and the PERCATHE and PINpOINT environments for feedback on the study. We also thank Dr. M. Zucknick for suggesting the drug withdrawal test. This study was supported by grants from the Norwegian Health Authority South-East, grant numbers 2017064, 2018012, and 2019096; the Norwegian Cancer Society, grant numbers 182524 and 208012; and the Research Council of Norway through its Centers of Excellence funding scheme (262652) and through grants 261936, 294916, and 314811. S.S.S. was supported by the Research Council of Norway under the frame of ERA PerMed (CLL-CLUE: 322898) and the Norwegian Center for Clinical Research (MATRIX: Research Council of Norway and the Norwegian Cancer Society joint grant 328827).

AUTHOR CONTRIBUTIONS

Conceptualization: A.N.A. and J.M.E. Project supervision: A.N.A., S.S.S., and J.M.E. Data analysis: A.M.B. and A.N.A. Prepared figures and tables: A.M.B. and A.N.A. Performed drug screens: P.A.-D. with assistance from L.P., D.S.T., L.B., and J.F. Diagnosed, sampled, and treated patients: Y.F. and A.L. Wrote the first draft: A.N.A., A.M.B., and J.M.E. Edited the manuscript: A.M.B., A.N.A., J.M.E., S.S.S., P.A.-D., Y.F., and A.L. Extracted patient data from records: M.S., P.A.-D., and A.M.B.

DECLARATION OF INTERESTS

A.L. serves on the advisory board of Janssen, AbbVie, Servier, and Astellas and has received research funding from Gilead. S.S.S. has received honoraria from AbbVie and AstraZeneca and research support from BeiGene and TG Therapeutics.

Received: March 15, 2023
Revised: September 16, 2023
Accepted: November 9, 2023
Published: December 7, 2023

REFERENCES

1. Löwenberg, B., Downing, J.R., and Burnett, A. (1999). Acute myeloid leukemia. N. Engl. J. Med. *341*, 1051–1062.
2. Kantarjian, H., Kadia, T., DiNardo, C., Daver, N., Borthakur, G., Jabbour, E., Garcia-Manero, G., Konopleva, M., and Ravandi, F. (2021). Acute myeloid leukemia: current progress and future directions. Blood Cancer J. *11*, 41.
3. Dombret, H., and Gardin, C. (2016). An update of current treatments for adult acute myeloid leukemia. Blood *127*, 53–61.
4. Döhner, H., Wei, A.H., Appelbaum, F.R., Craddock, C., DiNardo, C.D., Dombret, H., Ebert, B.L., Fenaux, P., Godley, L.A., Hasserjian, R.P., et al. (2022). Diagnosis and management of AML in adults: 2022 recommendations from an international expert panel on behalf of the ELN. Blood *140*, 1345–1377.
5. Falini, B., Nicoletti, I., Martelli, M.F., and Mecucci, C. (2007). Acute myeloid leukemia carrying cytoplasmic/mutated nucleophosmin (NPMc+ AML): biologic and clinical features. Blood *109*, 874–885.
6. Mrózek, K., Heinonen, K., and Bloomfield, C.D. (2001). Clinical importance of cytogenetics in acute myeloid leukaemia. Best Pract. Res. Clin. Haematol. *14*, 19–47.
7. Zeng, A.G.X., Bansal, S., Jin, L., Mitchell, A., Chen, W.C., Abbas, H.A., Chan-Seng-Yue, M., Voisin, V., van Galen, P., Tierens, A., et al. (2022). A cellular hierarchy framework for understanding heterogeneity and predicting drug response in acute myeloid leukemia. Nat. Med. *28*, 1212–1223.
8. Tislevoll, B.S., Hellesøy, M., Fagerholt, O.H.E., Gullaksen, S.-E., Srivastava, A., Birkeland, E., Kleftogiannis, D., Ayuda-Durán, P., Piechaczyk, L., Tadele, D.S., et al. (2023). Early response evaluation by single cell signaling profiling in acute myeloid leukemia. Nat. Commun. *14*, 115.
9. Levine, J.H., Simonds, E.F., Bendall, S.C., Davis, K.L., Amir, E.-A.D., Tadmor, M.D., Litvin, O., Fienberg, H.G., Jager, A., Zunder, E.R., et al. (2015). Data-Driven Phenotypic Dissection of AML Reveals Progenitor-like Cells that Correlate with Prognosis. Cell *162*, 184–197.
10. Behbehani, G.K., Samusik, N., Bjornson, Z.B., Fantl, W.J., Medeiros, B.C., and Nolan, G.P. (2015). Mass Cytometric Functional Profiling of Acute Myeloid Leukemia Defines Cell-Cycle and Immunophenotypic Properties That Correlate with Known Responses to Therapy. Cancer Discov. *5*, 988–1003.
11. Irish, J.M., Hovland, R., Krutzik, P.O., Perez, O.D., Bruserud, Ø., Gjertsen, B.T., and Nolan, G.P. (2004). Single cell profiling of potentiated phospho-protein networks in cancer cells. Cell *118*, 217–228.
12. Heuser, M., Wingen, L.U., Steinemann, D., Cario, G., von Neuhoff, N., Tauscher, M., Bullinger, L., Krauter, J., Heil, G., Döhner, H., et al. (2005). Gene-expression profiles and their association with drug resistance in adult acute myeloid leukemia. Haematologica *90*, 1484–1492.
13. Spinner, M.A., Aleshin, A., Santaguida, M.T., Schaffert, S.A., Zehnder, J.L., Patterson, A.S., Gekas, C., Heiser, D., and Greenberg, P.L. (2020). Ex vivo drug screening defines novel drug sensitivity patterns for informing personalized therapy in myeloid neoplasms. Blood Adv. *4*, 2768–2778.
14. Pemovska, T., Kontro, M., Yadav, B., Edgren, H., Eldfors, S., Szwajda, A., Almusa, H., Bespalov, M.M., Ellonen, P., Elonen, E., et al. (2013). Individualized systems medicine strategy to tailor treatments for patients with chemorefractory acute myeloid leukemia. Cancer Discov. *3*, 1416–1429.
15. Collignon, A., Hospital, M.A., Montersino, C., Courtier, F., Charbonnier, A., Saillard, C., D'Incan, E., Mohty, B., Guille, A., Adelaïde, J., et al. (2020). A chemogenomic approach to identify personalized therapy for patients with relapse or refractory acute myeloid leukemia: results of a prospective feasibility study. Blood Cancer J. *10*, 64.
16. Swords, R.T., Azzam, D., Al-Ali, H., Lohse, I., Volmar, C.-H., Watts, J.M., Perez, A., Rodriguez, A., Vargas, F., Elias, R., et al. (2018). Ex-vivo sensitivity profiling to guide clinical decision making in acute myeloid leukemia: A pilot study. Leuk. Res. *64*, 34–41.
17. Lin, L., Tong, Y., Straube, J., Zhao, J., Gao, Y., Bai, P., Li, J., Wang, J., Wang, H., Wang, X., et al. (2020). Ex-vivo drug testing predicts chemosensitivity in acute myeloid leukemia. J. Leukoc. Biol. *107*, 859–870.
18. Kornauth, C., Pemovska, T., Vladimer, G.I., Bayer, G., Bergmann, M., Eder, S., Eichner, R., Erl, M., Esterbauer, H., Exner, R., et al. (2022). Functional Precision Medicine Provides Clinical Benefit in Advanced Aggressive Hematologic Cancers and Identifies Exceptional Responders. Cancer Discov. *12*, 372–387.
19. Tyner, J.W., Yang, W.F., Bankhead, A., 3rd, Fan, G., Fletcher, L.B., Bryant, J., Glover, J.M., Chang, B.H., Spurgeon, S.E., Fleming, W.H., et al. (2013). Kinase pathway dependence in primary human leukemias determined by rapid inhibitor screening. Cancer Res. *73*, 285–296.
20. Tavor, S., Shalit, T., Chapal Ilani, N., Moskovitz, Y., Livnat, N., Groner, Y., Barr, H., Minden, M.D., Plotnikov, A., Deininger, M.W., et al. (2020). Dasatinib response in acute myeloid leukemia is correlated with FLT3/ITD, PTPN11 mutations and a unique gene expression signature. Haematologica *105*, 2795–2804.
21. Haverty, P.M., Lin, E., Tan, J., Yu, Y., Lam, B., Lianoglou, S., Neve, R.M., Martin, S., Settleman, J., Yauch, R.L., and Bourgon, R. (2016). Reproducible pharmacogenomic profiling of cancer cell line panels. Nature *533*, 333–337.
22. Mpindi, J.-P., Swapnil, P., Dmitrii, B., Jani, S., Saeed, K., Wennerberg, K., Aittokallio, T., Östling, P., and Kallioniemi, O. (2015). Impact of normalization methods on high-throughput screening data with high hit rates and drug testing with dose–response data. Bioinformatics *31*, 3815–3821.
23. Potdar, S., Ianevski, A., Mpindi, J.-P., Bychkov, D., Fiere, C., Ianevski, P., Yadav, B., Wennerberg, K., Aittokallio, T., Kallioniemi, O., et al. (2020). Breeze: an integrated quality control and data analysis application for high-throughput drug screening. Bioinformatics *36*, 3602–3604.
24. Safikhani, Z., Smirnov, P., Freeman, M., El-Hachem, N., She, A., Rene, Q., Goldenberg, A., Birkbak, N.J., Hatzis, C., Shi, L., et al. (2016). Revisiting inconsistency in large pharmacogenomic studies. F1000Res. *5*, 2333.
25. Fallahi-Sichani, M., Honarnejad, S., Heiser, L.M., Gray, J.W., and Sorger, P.K. (2013). Metrics other than potency reveal systematic variation in responses to cancer drugs. Nat. Chem. Biol. *9*, 708–714.
26. Garnett, M.J., Edelman, E.J., Heidorn, S.J., Greenman, C.D., Dastur, A., Lau, K.W., Greninger, P., Thompson, I.R., Luo, X., Soares, J., et al. (2012). Systematic identification of genomic markers of drug sensitivity in cancer cells. Nature *483*, 570–575.
27. Yadav, B., Pemovska, T., Szwajda, A., Kulesskiy, E., Kontro, M., Karjalainen, R., Majumder, M.M., Malani, D., Murumägi, A., Knowles, J., et al.

CellPress
OPEN ACCESS

(2014). Quantitative scoring of differential drug sensitivity for individually optimized anticancer therapies. Sci. Rep. *4*, 5193.

28. Mpindi, J.P., Yadav, B., Östling, P., Gautam, P., Malani, D., Murumägi, A., Hirasawa, A., Kangaspeska, S., Wennerberg, K., Kallioniemi, O., and Aittokallio, T. (2016). Consistency in drug response profiling. Nature *540*, E5–E6.
29. Safikhani, Z., El-Hachem, N., Smirnov, P., Freeman, M., Goldenberg, A., Birkbak, N.J., Beck, A.H., Aerts, H.J.W.L., Quackenbush, J., and Haibe-Kains, B. (2016). Safikhani et al. reply. Nature *540*, E11–E12.
30. Wang, D., Hensman, J., Kutkaite, G., Toh, T.S., Galhoz, A., GDSC Screening Team; Dry, J.R., Saez-Rodriguez, J., Garnett, M.J., Menden, M.P., and Dondelinger, F. (2020). A statistical framework for assessing pharmacological responses and biomarkers using uncertainty estimates. Elife *9*, e60352.
31. Ianevski, A., Giri, A.K., and Aittokallio, T. (2020). SynergyFinder 2.0: visual analytics of multi-drug combination synergies. Nucleic Acids Res. *48*, W488–W493.
32. Staib, P., Staltmeier, E., Neurohr, K., Cornely, O., Reiser, M., and Schinköthe, T. (2005). Prediction of individual response to chemotherapy in patients with acute myeloid leukaemia using the chemosensitivity index Ci. Br. J. Haematol. *128*, 783–791.
33. Onecha, E., Ruiz-Heredia, Y., Martínez-Cuadrón, D., Barragán, E., Martinez-Sanchez, P., Linares, M., Rapado, I., Perez-Oteyza, J., Magro, E., Herrera, P., et al. (2020). Improving the prediction of acute myeloid leukaemia outcomes by complementing mutational profiling with ex vivo chemosensitivity. Br. J. Haematol. *189*, 672–683.
34. Zhang, Y., Ji, M., Zhao, J.-Y., Wang, H.-F., Wang, C.-W., Li, W., Ye, J.-J., Lu, F., Lin, L.-H., Gao, Y.-T., et al. (2021). Ex Vivo Chemosensitivity Profiling of Acute Myeloid Leukemia and Its Correlation With Clinical Response and Outcome to Chemotherapy. Front. Oncol. *11*, 793773.
35. Martínez-Cuadrón, D., Gil, C., Serrano, J., Rodríguez, G., Pérez-Oteyza, J., García-Boyero, R., Jiménez-Bravo, S., Vives, S., Vidriales, M.B., Lavilla, E., et al. (2019). A precision medicine test predicts clinical response after idarubicin and cytarabine induction therapy in AML patients. Leuk. Res. *76*, 1–10.
36. Zou, H., and Hastie, T. (2005). Regularization and variable selection via the elastic net. J. Roy. Stat. Soc. B *67*, 301–320.
37. Tazi, Y., Arango-Ossa, J.E., Zhou, Y., Bernard, E., Thomas, I., Gilkes, A., Freeman, S., Pradat, Y., Johnson, S.J., Hills, R., et al. (2022). Unified classification and risk-stratification in Acute Myeloid Leukemia. Nat. Commun. *13*, 4622.
38. Dempster, J.M., Rossen, J., Kazachkova, M., Pan, J., Kugener, G., Root, D.E., and Tsherniak, A. Extracting Biological Insights from the Project Achilles Genome-Scale CRISPR Screens in Cancer Cell Lines.
39. DiNardo, C.D., Jonas, B.A., Pullarkat, V., Thirman, M.J., Garcia, J.S., Wei, A.H., Konopleva, M., Döhner, H., Letai, A., Fenaux, P., et al. (2020). Azacitidine and Venetoclax in Previously Untreated Acute Myeloid Leukemia. N. Engl. J. Med. *383*, 617–629.
40. Campos, L., Rouault, J.P., Sabido, O., Oriol, P., Roubi, N., Vasselon, C., Archimbaud, E., Magaud, J.P., and Guyotat, D. (1993). High expression of bcl-2 protein in acute myeloid leukemia cells is associated with poor response to chemotherapy. Blood *81*, 3091–3096.
41. Krawiec, K., Strzałka, P., Czemerska, M., Wiśnik, A., Zawlik, I., Wierzbowska, A., and Pluta, A. (2022). Targeting Apoptosis in AML: Where Do We Stand? Cancers *14*, 4995.
42. Haibe-Kains, B., El-Hachem, N., Birkbak, N.J., Jin, A.C., Beck, A.H., Aerts, H.J.W.L., and Quackenbush, J. (2013). Inconsistency in large pharmacogenomic studies. Nature *504*, 389–393.
43. Geeleher, P., Cox, N.J., and Huang, R.S. (2016). Cancer biomarker discovery is improved by accounting for variability in general levels of drug sensitivity in pre-clinical models. Genome Biol. *17*, 190.
44. White, B.S., Khan, S.A., Mason, M.J., Ammad-Ud-Din, M., Potdar, S., Malani, D., Kuusanmäki, H., Druker, B.J., Heckman, C., Kallioniemi, O., et al. (2021). Bayesian multi-source regression and monocyte-associated gene expression predict BCL-2 inhibitor resistance in acute myeloid leukemia. npj Precis. Oncol. *5*, 71.
45. Pacini, C., Dempster, J.M., Boyle, I., Gonçalves, E., Najgebauer, H., Karakoc, E., van der Meer, D., Barthorpe, A., Lightfoot, H., Jaaks, P., et al. (2021). Integrated cross-study datasets of genetic dependencies in cancer. Nat. Commun. *12*, 1661.
46. Mengie Ayele, T., Tilahun Muche, Z., Behaile Teklemariam, A., Bogale Kassie, A., and Chekol Abebe, E. (2022). Role of JAK2/STAT3 Signaling Pathway in the Tumorigenesis, Chemotherapy Resistance, and Treatment of Solid Tumors: A Systemic Review. J. Inflamm. Res. *15*, 1349–1364.
47. Xu, P., Peng, M., Zhou, D., Shao, X., Zhang, Q., Zhou, R., Xu, J., Ouyang, J., and Chen, B. (2017). The polymorphism of JAK2 rs56118985 may be a predictive marker of the treatment responses of acute myeloid leukemia patients. Transl. Cancer Res. *6*, 779–787.
48. Li, F., Lu, Z.-Y., Xue, Y.-T., Liu, Y., Cao, J., Sun, Z.-T., Zhang, Q., Xu, M.-D., Wang, X.-Y., Xu, K.-L., and Wu, Q.Y. (2023). Molecular basis of JAK2 H608Y and H608N mutations in the pathology of acute myeloid leukemia. Int. J. Biol. Macromol. *229*, 247–259.
49. Tyner, J.W., Tognon, C.E., Bottomly, D., Wilmot, B., Kurtz, S.E., Savage, S.L., Long, N., Schultz, A.R., Traer, E., Abel, M., et al. (2018). Functional genomic landscape of acute myeloid leukaemia. Nature *562*, 526–531.
50. Bottomly, D., Long, N., Schultz, A.R., Kurtz, S.E., Tognon, C.E., Johnson, K., Abel, M., Agarwal, A., Avaylon, S., Benton, E., et al. (2022). Integrative analysis of drug response and clinical outcome in acute myeloid leukemia. Cancer Cell *40*, 850–864.e9.
51. Malani, D., Kumar, A., Brück, O., Kontro, M., Yadav, B., Hellesøy, M., Kuusanmäki, H., Dufva, O., Kankainen, M., Eldfors, S., et al. (2022). Implementing a Functional Precision Medicine Tumor Board for Acute Myeloid Leukemia. Cancer Discov. *12*, 388–401.
52. Chen, J., Odenike, O., and Rowley, J.D. (2010). Leukaemogenesis: more than mutant genes. Nat. Rev. Cancer *10*, 23–36.
53. Lewis, A.C., and Kats, L.M. (2021). Non-genetic heterogeneity, altered cell fate and differentiation therapy. EMBO Mol. Med. *13*, e12670.
54. Pietarinen, P.O., Eide, C.A., Ayuda-Durán, P., Potdar, S., Kuusanmäki, H., Andersson, E.I., Mpindi, J.P., Pemovska, T., Kontro, M., Heckman, C.A., et al. (2017). Differentiation status of primary chronic myeloid leukemia cells affects sensitivity to BCR-ABL1 inhibitors. Oncotarget *8*, 22606–22615.
55. Zhang, J.H., Chung, T.D., and Oldenburg, K.R. (1999). A Simple Statistical Parameter for Use in Evaluation and Validation of High Throughput Screening Assays. J. Biomol. Screen *4*, 67–73.
56. Simon, N., Friedman, J., Hastie, T., and Tibshirani, R. (2011). Regularization Paths for Cox's Proportional Hazards Model via Coordinate Descent. J. Stat. Software *39*, 1–13.
57. Harrell, F.E., Califf, R.M., Pryor, D.B., Lee, K.L., and Rosati, R.A. (1982). Evaluating the Yield of Medical Tests. JAMA, J. Am. Med. Assoc. *247*, 2543–2546.
58. Yu, G., Wang, L.-G., Han, Y., and He, Q.-Y. (2012). clusterProfiler: an R Package for Comparing Biological Themes Among Gene Clusters. OMICS A J. Integr. Biol. *16*, 284–287.

STAR★METHODS

KEY RESOURCES TABLE

REAGENT or RESOURCE	SOURCE	IDENTIFIER
Biological samples		
Patient-derived mononuclear cells isolated from bone marrow aspirates and/or peripheral blood samples	Department of Haematology at Oslo University Hospital in Norway	N/A
Chemicals, peptides, and recombinant proteins		
Selleck Anti-Cancer Compound Library L3000, 349 compounds	Selleck Chemicals	Z88971
Benzethonium chloride, BzCl	Santa Cruz Biotechnology	Sc-239299
Critical commercial assays		
LymphoPrep^{TM} gradient centrifugation	Stemcell	# 07801
Mononuclear Cell Medium (MCM)	PromoCell	C-28030
CellTiter-Glo 2.0	Promega	G9243
Deposited data		
Clinical data	This study	Request to lead contact
Dose-response data (Dose response data and Hill curve fits_2023-07.csv)	This study	https://doi.org/10.5281/zenodo.10055032
Drug information data (Selleck_Drug information.xlsx)	This study	https://doi.org/10.5281/zenodo.10055032
Software and algorithms		
Statistical programming environment R	The R Project for Statistical Computing	https://www.r-project.org/, version 4.2.1
R scripts	This study	https://zenodo.org/doi/10.5281/zenodo.10055032
Other		
Echo 550 liquid handling robot	Labcyte Inc.	Center for Molecular Medicine Norway, University of Oslo, https://www.med.uio.no/english/research/core-facilities/chemical-biology-screening/
EnVision 2104 Multilabel plate reader	Perkin Elmer	N/A

RESOURCE AVAILABILITY

Lead contact

Further information and requests should be directed to Jorrit M. Enserink: jorrit.enserink@ibv.uio.no.

Materials availability

This study did not generate new unique reagents.

Data and code availability

- The clinical data in their complete form are freely available from the lead contact upon request.
- The drug screen data and all code is freely available at https://github.com/Enserink-lab/DSCoxTools. DOIs are listed in the key resources table.
- Any additional information required to reanalyze the data reported in this work is available from the lead contact upon request.

EXPERIMENTAL MODEL AND STUDY PARTICIPANT DETAILS

Patient cohort

Bone marrow aspirates and/or peripheral blood samples were collected from 69 adult patients diagnosed with AML and treated at the Department of Haematology at Oslo University Hospital in Norway between 2015 and 2018 (two relapse samples were collected

CellPress
OPEN ACCESS

in 2019). 60 of the patients were treatment naive and nine had undergone previous treatment (Tables S1 and S1A). From eight of the 69 patients we also received a sample at relapse. Drug screening data for the relapse samples were excluded from modeling.

The study was performed in accordance with the Declaration of Helsinki and samples were collected following written informed consent. The study was approved by the Regional Committee for Medical Research Ethics South-East Norway (REK 2015/2012). Patient data that were collected included survival, sex, age, WHO patient performance status, routine diagnostic workup consisting of flow cytometry and genetic biomarkers investigated by G-banding, RT-qPCR, FISH and fragment analysis of FLT3 and NPM1, as well as ELN2022 risk stratification and FAB classification (see Table S1 for an overview of patient characteristics). The median age was 60 years. Most patients were subjected to standard treatment, which consisted of a 30 min infusion of anthracycline [daunorubicin (60 mg/m^2) or idarubicin (10–12 mg/m^2)] for three days in combination with a 24-h infusion of cytarabine (Ara-C) for seven days, although the length and dosage of each treatment were in some cases altered depending on the patient's age and general condition. Some patients received other drugs in addition to standard treatment (see Table S2 for details).

METHOD DETAILS

Ex vivo drug screening

The experimental procedures were designed following methods that were state-of-the-art at the time of study start.[14] In brief, fresh bone marrow or peripheral blood samples were collected in tubes with heparin or EDTA at the Department of Haematology at Oslo University Hospital (see Table S3). Typically, the samples were processed within 60 min after the sample was collected. In a few rare instances, when patient samples were collected at night or during the weekend, the samples were stored at 4°C for up to 16 h before processing. Mononuclear cells were subsequently purified as previously described[8] using Lymphoprep gradient centrifugation (Stemcell) and cultured in Mononuclear Cell Medium (MCM, PromoCell C-28030) supplemented with 1% Penicillin + Streptomycin (PS) (Gibco,15140-122).

A total of 10,000 cells (25 μL) in MCM + PS was added to each well in pre-drugged 384-well plates (Greiner Bio-One) using a Multi Drop Combi peristaltic dispenser (Thermo Scientific). The number of cells was adjusted for specific samples with lower cell counts without a noticeable change in the outcome of the experiments. The Selleck Anti-Cancer Compound Library L3000 was used for drug screening, which consists of 349 anti-cancer drugs dissolved in dimethyl sulfoxide (DMSO) (see Table S2 for the complete list). The compounds were distributed in seven 384-well plates using five 10-fold dilution steps (1 nM–10.000 nM). Eight positive (benzethonium chloride, BzCl) and negative (DMSO only) controls were added to each plate. Drug handling was performed at the Center for Molecular Medicine Norway, University of Oslo, using an Echo 550 liquid handling robot (Labcyte Inc.). After incubation for 72 h at 37°C in a humidified environment with 5% CO_2, relative cell viability was quantified using the CellTiter-Glo 2.0 Cell Viability Assay (Promega), as a fast, sensitive, and high-throughput friendly readout, which is also used as a standard readout in other centers.[14,54] The luminescence readout was measured in an EnVision 2104 Multilabel plate reader (PerkinElmer) in counts per second (CPS). Relative cell viability was computed from CPS values normalized to negative (DMSO) and positive (BzCl) controls within each plate.

Dose-response analysis

Relative cell viabilities (y) were computed from CPS values using min-max normalization to the median CPS of the positive and negative controls within each plate. All negative response values were adjusted to zero. Plate quality was assessed by a Z′-factor and strictly standardized mean difference (SSMD) of the plate controls. The Z′-factor measures the difference between positive plate controls (BzCl) and negative plate controls (DMSO). These controls were included in each assay plate for each patient sample. A Z′-factor between 0.5 and 1 is considered excellent, values between 0 and 0.5 may be acceptable, while values below 0 are indicative of a poor essay.[55] The average drug sensitivity was measured as a normalized rectangular area under the curve (rAUC) of the raw relative viability response for every patient-drug combination using the formula:

$$rAUC = \sum_{c=2}^{5} \frac{y_c(\log x_c - \log x_{c-1})}{\log x_5 - \log x_1}$$

Here x_c indicates a given concentration with relative viability y_c, ranging from the lowest concentration x_1 to the highest concentration x_5. Before their use in models all rAUCs were centered around zero inhibition: $1 - rAUC$. Alternatively, the rAUCs were negative $\log_2$-transformed ($-\log_2(rAUC)$) to counter the skewness of the relative viability scale.

To score drug sensitivities using metrics based on dose-response curve fitting we used routines adapted from the Breeze application.[23] Briefly, Breeze transforms the scale to percent inhibition and the following parametrization of the Hill equation is fitted to the data:

$$f(x) = R_{min} + \frac{(R_{max} - R_{min})}{1 - 10^{n(\log EC50 - \log x)}}$$

where R_{min} is the baseline inhibition (typically held to zero), R_{max} is the maximum inhibitory response for the drug, and n is the slope (Hill coefficient). In Breeze, only growth inhibitory responses are considered. The algorithm constrains the curve fits with $R_{min} = 0$, $n = [0.1, 2.5]$ and $R_{max} = [0, 100]$. All EC50s are constrained to a value between the maximum and minimum concentration of the experiment. EC50 values for non-inhibitory responses are set to the maximum concentration. TEC50 is computed by setting

EC50 to the maximum concentration for the models with R_{max} under 25%. Before further use, both EC50 and TEC50 were log-transformed and mean-subtracted per drug.

The drug sensitivity score (DSS) is computed from an analytical solution to the integral ,

$$I = \int_{x_t}^{x_5} f(x)dx$$

where x_t is the concentration at an activity threshold t set to 10%. Thus, DSS can only take positive values with $DSS1 = \frac{(I - t(\log x_5 - \log x_t))}{((100 - t)(\log x_5 - \log x_1))}$, $DSS2 = DSS\frac{1}{\log R_{max}}$, and $DSS3 = DSS2\frac{(x_5 - x_t)}{(x_5 - x_1)}$[27].

We also used Breeze to compute an AUC based on a LOESS curve fit (referred to as loess-AUC), which models both growth promoting and inhibitory responses. For bidirectional Hill curve fitting, we modified the Breeze algorithm by setting $R_{max} = [-100, 100]$, and thus allowing fitting of non-inhibitory responses. Hill-rAUC was computed by predicting the relative viability and using the rAUC formula as indicated before.

Patient-wise standardization of drug sensitivity metrics (s), was performed as follows to compute a drug sensitivity Z score:

$$z_{dr,pt} = \frac{s_{dr,pt} - \mu_{pt}}{\sigma_{pt}}$$

Here μ_{pt} and σ_{pt} is the drug sensitivity mean and standard deviation per patient respectively. For scaling of the drugs, a drug-wise standardization procedure was done over the drug sensitivity metrics or drug sensitivity z-scores.

Clinical data processing

The clinical data were grouped into two feature sets, with binarized dummy variables for categorical data. Clinical prognostic features included age at the time of diagnosis, sex, WHO patient performance status, and ELN2022 risk stratifications. Genetic features included specific mutations and chromosomal rearrangements with coverage for at least three patients. Missing data were zero-imputed. The survival times were computed from the date of diagnosis until the registered date of death, and censoring times were computed to the last recorded visiting date. Response to induction was binarized based on reaching blast clearance (remission) or not after induction therapy. Relapse was binarized for the sub-population of patients having reached complete remission.

Model training and cross-validation

Regularized Cox models were trained on the different drug sensitivity metrics and the binarized clinical variables using the glmnet package.[56] Ridge, Lasso, or Elastic net models were trained by setting the penalty mixture parameter (α) to 0, 1, or 0.4, respectively, unless otherwise specified, and screening over a sequence of penalties using leave-one-out cross-validation (due to the low sample size). The model with the best average cross-validation (partial likelihood) deviance score was selected for testing or further analysis of model coefficients. The Cox model coefficients represent the change in log-hazard ratio (log-HR) as a function of drug sensitivity. Regularized binomial models were used for logistic regression of binarized clinical outcomes, and optimized using the same procedure.

Pre-selection of model features prior to model training was based on thresholds for standard deviations in drug sensitivity (as indicated), where the number of drugs with the greatest drug sensitivity standard deviations were selected. For a random sampling of features (p = 100), the rAUC standard deviation was used as sampling weights.

Model testing

For testing the models, a random sample of 10 patients was withheld from the training procedure, with a fixed proportion of deceased to surviving patients to maintain the approximate proportions in the complete dataset (5 deceased and 5 surviving for the full survival models, and 4 deceased and 6 surviving for the short term survival models). The prediction accuracy on test data was scored with Harrell's concordance index (C-index), which defines the proportion of patient pairs with concordance between their observed survival times and predicted risk. It is especially suited for clinical studies as it takes into account the censoring of patients where data is not recorded after a given point.[57] The training and testing procedure was repeated 50 or 200 times, as indicated. For testing of models predicting binary clinical outcomes, a 5-fold training and testing procedure was performed and prediction accuracy was measured using an area under the receiver operating characteristic curve (ROC AUC).

Analysis of confounding factors

Principal component analysis (PCA) was performed using singular value decomposition (SVD) on scaled matrices of drug sensitivity metrics or z-scores, and removal of confounding principal components (RCPC) was performed by reconstructing the datasets after removing the leading singular values and singular vectors, followed by rescaling of the metrics (see Figure S3A). SVD was performed on scaled matrices of drug sensitivity metrics (or z-scores) such that $\frac{(s_{dr,pt} - \mu_{dr})}{\sigma_{dr}} = \sum_k u_{dr,k}\sqrt{\lambda_k}v_{k,pt}$, where $u_{dr,k}$ and $v_{k,pt}$ are components of $\boldsymbol{u}_k$ and $\boldsymbol{v}_k$ which are left and right singular vectors of the principal axis k with variance λ_k. The variance explained by each principal component was computed as $\rho_k = \frac{\lambda_k}{\sum_k \lambda_k}$.

CellPress
OPEN ACCESS

The association of specific sample characteristics with a principal component was assessed using linear regression against $\boldsymbol{v}_k$ and measured with the adjusted r-squared (r^2_{adj}). The patient sample characteristics with the following features were evaluated: log average CPS signal for DMSO and BzCl; average CPS noise measured as the coefficient of variation for DMSO and BzCl as well as their SSMD; the number of curve fit non-responders measured as the number of DSS3 values equal zero or EC50 or TEC50 values at maximum; curve fit error features, which included the number of low-confidence curve fits reported by Breeze, and the average log-EC50 standard error, mean absolute error and max residual error per patient reported by Breeze; batch covariates, which included time period clusters for screen executions (defined using k-means clustering), instrument batch, cell seeding number (over or under 10000 cells) and blast source (PBMC or BM); biological/clinical covariates, which included genetic and cytogenetic features, age, sex, ELN2022 risk stratifications, clinical history (primary AML or secondary after antecedent myelodysplastic syndromes or other causes), and AML FAB classifications. Cumulative variance explained by a sample characteristic up to a component K was calculated as $\sum_{k=1}^{K} \rho_k r^2_{adj,k}$.

PCA on dose-response data was done such that $\frac{(y_{(dr,c),pt} - \mu_{(dr,c)})}{\sigma_{(dr,c)}} = \sum_k u_{(dr,c),k} \sqrt{\lambda_k} v_{k,pt}$, where $y_{(dr,c),pt}$ represent the raw, processed or Hill fitted drug response or curve fit residuals of a patient (columns) and a drug at a specific concentration (rows). When indicated, standardization was applied over the columns.

To test the removal of potentially confounding principal components, the drug sensitivity metrics (or z-scores) were reconstructed as $s'_{dr,pt} = \sigma_{dr} \sum_{k>l} u_{dr,k} \sqrt{\lambda_k} v_{k,pt} + \mu_{dr}$, where l represents the minimum number of principal components removed. RCPC testing was performed for different Cox models and different dataset sizes based on the feature pre-selection described earlier (Figures 4A, 4B, S4A, and S4B) and for Lasso trained on 50 randomly sampled datasets (Figures 4C and S4C—S4E).

Variable importance

The importance of individual drugs was assessed by their contribution to predictivity through a variable withdrawal test, and the statistical significance of the model coefficients. The contribution to predictivity was measured by training and testing models leaving out individual drugs one-by-one and computing the change in C-index from the model containing all drugs. The statistical significance of model coefficients was estimated by training 200 models on bootstrapped datasets and computing the mean and confidence interval for the fitted coefficients.

Model coefficient clustering

Estimated Ridge coefficients from models predicting different clinical outcomes were normalized by the standard deviation, and drugs with a normalized coefficient greater than three were selected for hierarchical clustering using euclidean distance and ward.D2. Before the clustering, the sign of coefficients for the different models were harmonized such that positives and negatives were associated with unfavorable and favorable clinical outcomes, respectively.

Enrichment test

Parametric enrichment analysis was performed using the GSEA function from ClusterProfiler based on ranked Ridge model coefficients or differential drug sensitivity z-scores, where drug sets were defined based on drug target or class association.[58]

QUANTIFICATION AND STATISTICAL ANALYSIS

Statistical significance was assessed using paired Wilcoxon test for Figures 2F, 3B, 3C, S2E, S4B, and S4E. For predicted hazard ratio comparison between relapse and treatment-naïve samples in Figures 2F and S2E, data points were paired based on patient identity (n = 8). For the other Fig., data points were paired based on methodological parameters (Figures 3B and S4B) or random sample identity (Figures 3C and S4E). Significance was indicated for p values ≤ 0.05.

The similarities in drug sensitivity profiles between drugs or between samples/patients in Figures 1F, S1C, S1D, S1E, S1C, and S3E, were measured using the Pearson correlation coefficient (PCC, or denoted as R in the scatterplots).

Statistical significance of drug-wise survival associations based on Ridge model coefficients in Figures 5A and S5, was determined based on the 95% confidence intervals, where significance (*) was determined when 95% of the bootstrapped coefficients did not include or cross zero.

For analysis of differential drug sensitivity z-scores between relapse and treatment-naïve samples in Figure 5G, statistical significance was evaluated using a paired t-test, with pairing based on patient identity (n = 8). Significance was indicated for p values $\leq$ 0.05. Statistical significance of enriched drug sets in Figures 5F, 5H, and S5F—S5I were assessed using the permutation test option of GSEA.

Cell Reports Methods, Volume *3*

Supplemental information

Clinical forecasting of acute myeloid leukemia

using *ex vivo* drug-sensitivity profiling

Aram N. Andersen, Andrea M. Brodersen, Pilar Ayuda-Durán, Laure Piechaczyk, Dagim Shiferaw Tadele, Lizet Baken, Julia Fredriksen, Mia Stoksflod, Andrea Lenartova, Yngvar Fløisand, Sigrid S. Skånland, and Jorrit M. Enserink

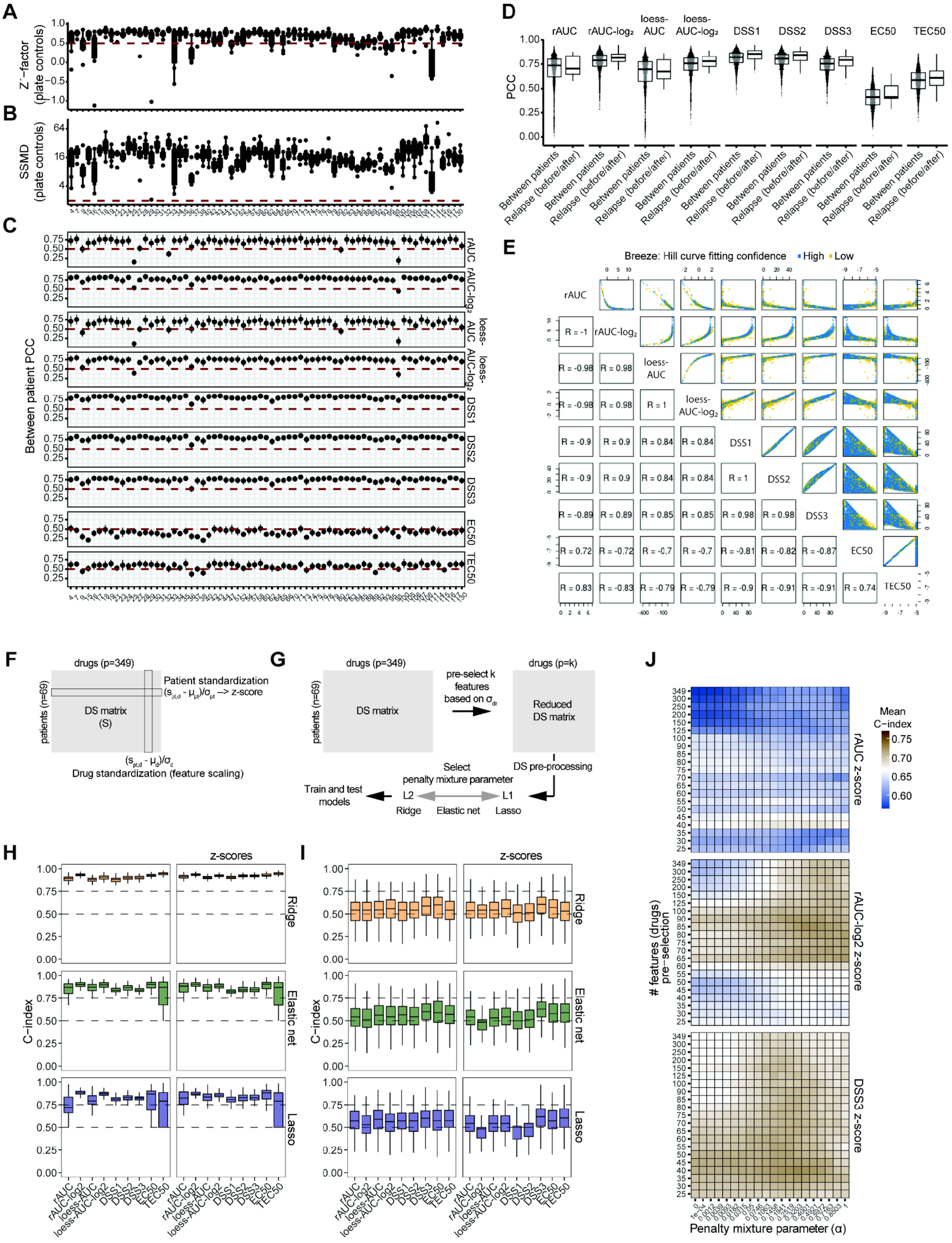

Figure S1. Drug screen quality controls, related to Figure 1. ***A,*** Z′-factor for DMSO and BzCl plate controls per patient. ***B,*** SSMD for DMSO and BzCl plate controls per patient. ***C,*** Mean between-patient drug sensitivity profile correlation per patient for different drug

sensitivity metrics. Dot and bar represent the mean and standard deviation for one patient against the remaining cohort. ***D,*** Correlations in drug sensitivity profiles, either between patients or for the same patient before and after relapse. ***E,*** Correlations between the different drug sensitivity metrics. High and low confidence Hill curve fits reported by Breeze are color-coded. ***F,*** Standardization procedures used in the study. Drug sensitivity z-scores were generated by standardizing each patient distribution. Drug sensitivity feature scaling was done by standardizing each drug distribution. ***G,*** Overview of feature pre-selection procedure and penalty type testing. ***H,*** Training C-index results (200 tests) for Cox models trained on different drug sensitivity metrics (*left*) or drug sensitivity z-scores (*right*). ***I,*** Test C-index results (200 tests) for Cox models trained on different drug sensitivity metrics (*left*) or drug sensitivity z-scores (*right*) with feature scaling (drug-wise standardization). ***J,*** Mean test C-index results (50 tests) for Cox models trained on rAUC, rAUC-$\log_2$, or DSS3 z-scores with various penalty mixture parameters (α) and feature pre-selection thresholds based on standard deviations of the respective drug sensitivity metric (rAUC, rAUC-$\log_2$, or DSS3).

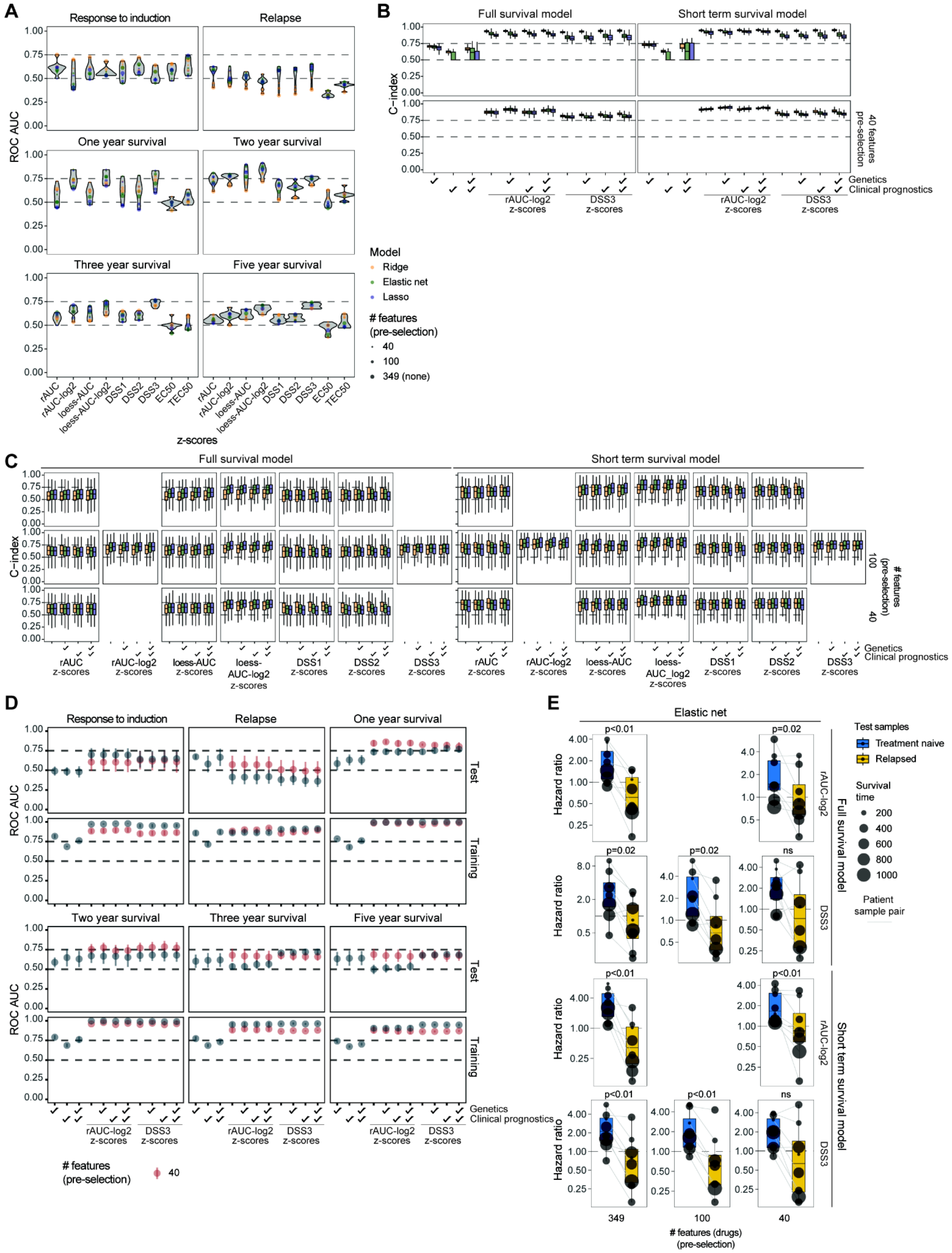

Figure S2. Supplement to versatility of drug sensitivity profiling for clinical outcome predictions, related to Figure 2. ***A,*** Average ROC-AUC score from 5-fold testing of classification of various binarized clinical outcomes using different drug sensitivity metrics,

model penalties and pre-selection thresholds. ***B,*** Training C-index results (200 tests) for Cox models trained on different dataset compositions based on clinical feature sets and rAUC-log_2 or DSS3 z-scores. The lower panels represents pre-selection of 40 features based on rAUC standard deviations, and the right panels represent prediction results from short term survival modeling. ***C,*** Test C-index results (200 tests) for Cox models trained on different dataset compositions based on clinical feature sets and drug sensitivity z-scores with different feature pre-selection cutoffs. Results from short term survival modeling are shown on the right. ***D,*** Testing and training ROC-AUC scores for classification of various binarized clinical outcomes using Ridge models trained on different dataset compositions based on clinical feature sets and rAUC-log_2 or DSS3 z-scores. The dot and bar indicate mean and standard deviation from 5-fold testing and training. E, Predicted hazard ratio (relative to population-median) on treatment-naive and relapsed samples for Elastic Net survival models trained on rAUC-log_2 or DSS3 z-scores with or without feature pre-selection. Bottom two panel rows represent short term survival modeling.

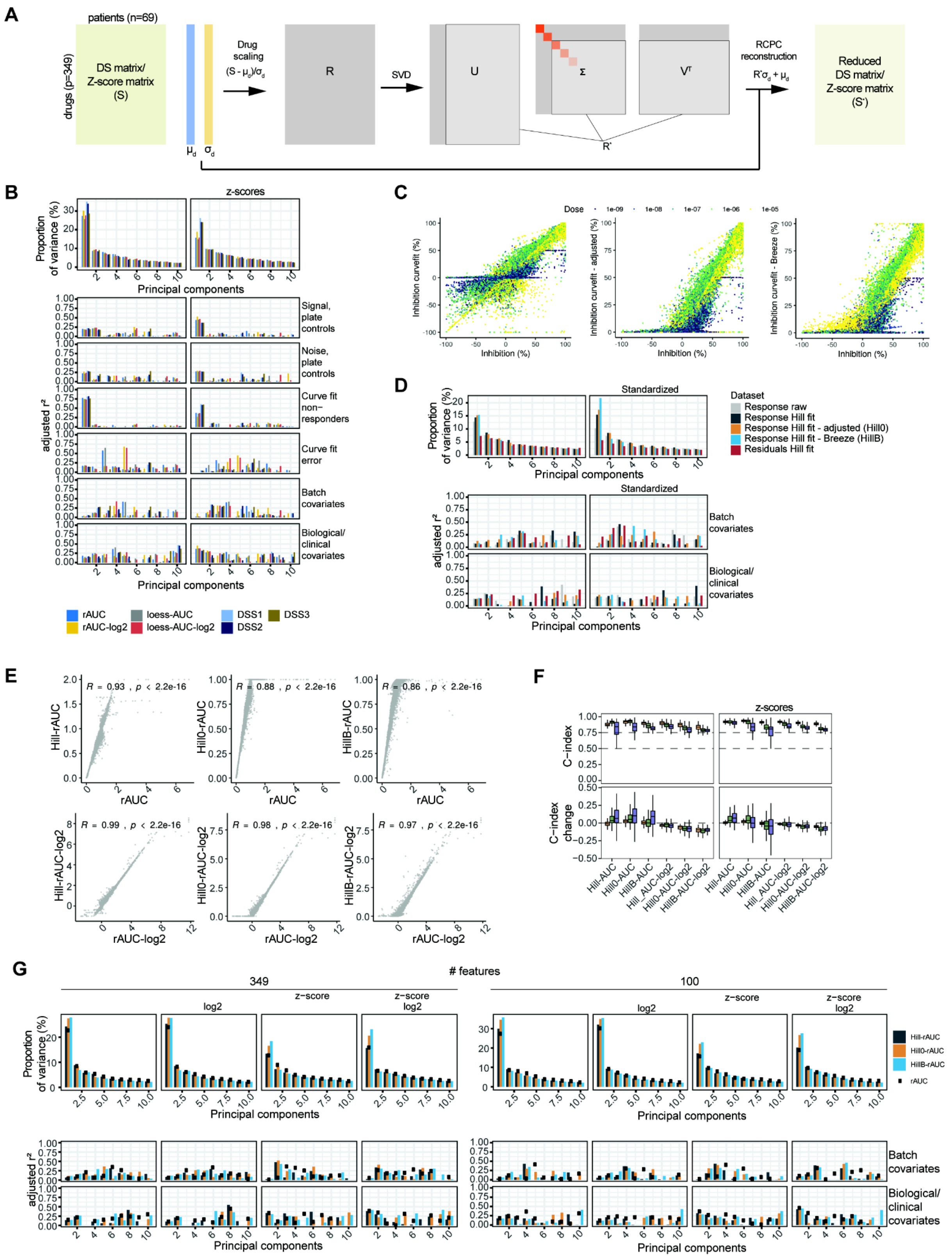

Figure S3. Supplement to exploration of confounding factors with PCA, related to Figure 3. ***A,*** Overview of PCA procedure based on SVD, and reconstruction using RCPC. ***B,*** Percent variance explained by principal components (*upper panels*) and principal component

variance explained by different patient sample characteristics (*lower panels*) for different drug sensitivity metrics and z-scores with feature pre-selection (100 features) based on rAUC standard deviations. ***C,*** Comparison between observed inhibition and Hill curve predicted inhibition. *Left panel* compares with Hill curve fitting for both inhibitory and non-inhibitory drug responses, *middle panel* compares with Hill curve fitting where non-inhibitory responses are set to zero, and the *right panel* compares with Hill curve fitting of inhibitory responses using Breeze. ***D,*** Percent variance explained by principal components (*upper panels*) and principal component variance explained by different patient sample characteristics (*lower panels*) for different non-standardized (*left*) and standardized (*right*) dose response datasets from ***C***, as well as the Hill curve fitting residuals. ***E,*** Comparison between Hill-based rAUC or rAUC-log_2 scores with non-curve fit raw rAUC or rAUC-log_2 scores. ***F,*** Training C-index results (200 tests) for Cox models trained on Hill-based rAUC or rAUC-log_2 scores or corresponding z-scores (*upper panels*), and their computed C-index change from the non-curve fit raw rAUC or rAUC-log_2 counterparts (*lower panels*). ***G,*** Percent variance explained by principal components (*upper panels*) and principal component variance explained by different patient sample characteristics (*lower panels*) for different Hill-based rAUC or rAUC-log_2 scores or corresponding z-scores with or without feature pre-selection based on rAUC standard deviations. The dots represent the corresponding results from the non-curve fit raw rAUC or rAUC-log_2 counterparts.

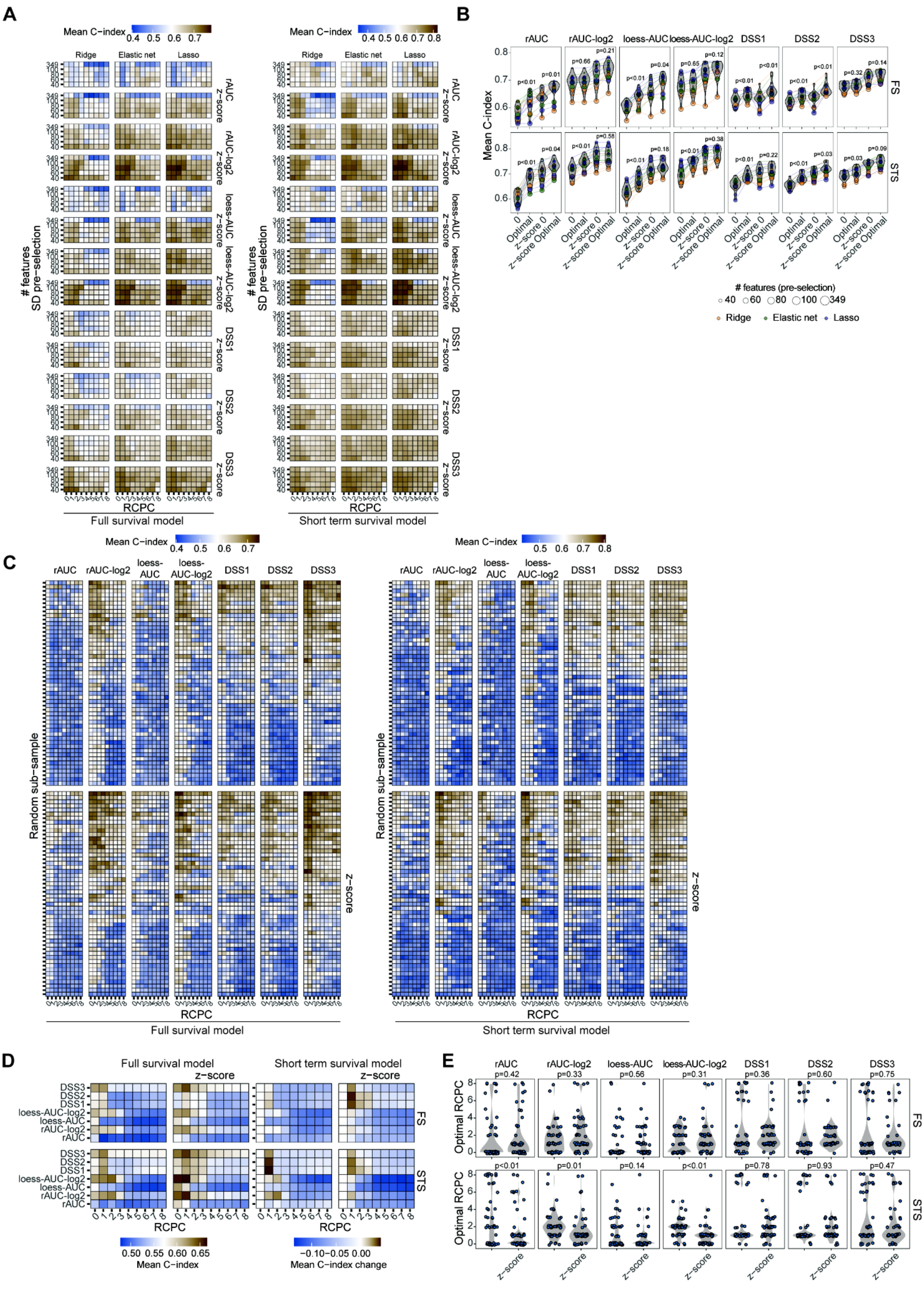

Figure S4. Supplement for testing removal of confounding principal components, related to Figure 4. ***A,*** Mean test C-index results (50 tests) for Ridge, Elastic net, and Lasso

survival models trained on different drug sensitivity metrics or z-scores with various feature pre-selection thresholds (based on rAUC standard deviations), and different numbers of principal components removed. *Left panels* represent results for full survival models and *right panels* represent results for short term survival models. ***B,*** Mean test C-index results (50 tests) for different survival models comparing zero or the optimal number of components removed for different drug sensitivity metrics or z-scores (shown in S6A). The *lower panels* represent results for short term survival models. p-values were computed using a paired Wilcoxon test. ***C,*** Mean test C-index results (50 tests) for Lasso survival models trained on 50 datasets generated under weighted random sampling of features, using different drug sensitivity metrics or z-scores, and different numbers of principal components removed. *Left panels* represent results for full survival models and *right panels* represent results for short term survival models. ***D,*** Sample average for test C-index (*left four panels*) or C-index change (*right four panels*) from the results in S6C. The C-index changes are computed from the respective reference datasets (RCPC = 0). ***E,*** Number of components removed to achieve the highest mean test C-index for different drug sensitivity metrics or z-scores in S6C. The *lower panels* represent results for short term survival models (STS). p-values were computed using a paired Wilcoxon test.

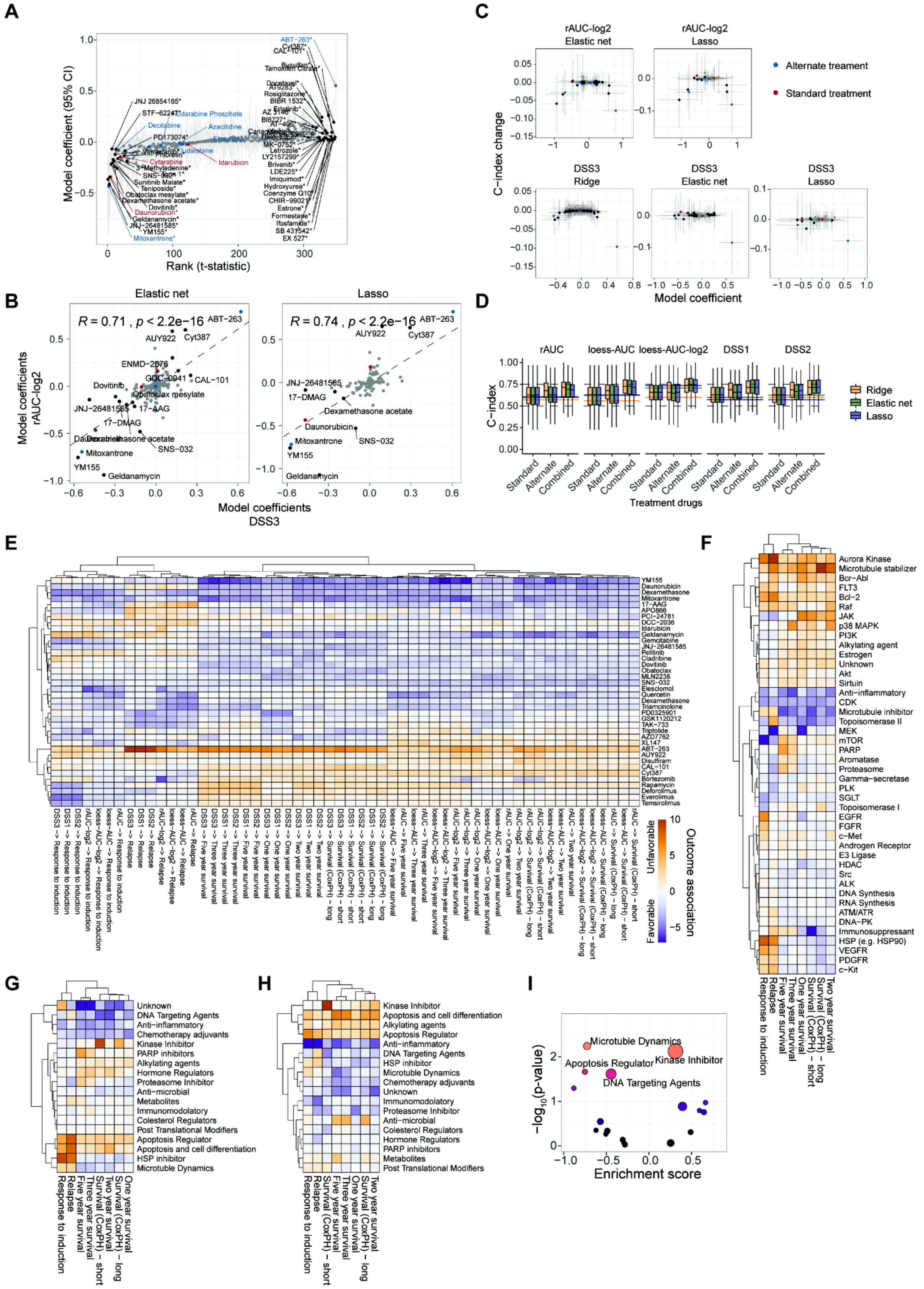

Figure S5 Supplement to clinical associations of *ex vivo* drug sensitivities, related to Figure 5. ***A*,** Bootstrapped Ridge survival coefficients representing risk association of DSS3

z-scores for 349 drugs. The vertical bars indicate the 95% confidence interval. Significance (*) was determined when 95% of the bootstrapped coefficients did not include or cross zero. ***B,*** Correlation between estimated Lasso and Elastic net survival coefficients for rAUC-$\log_2$ z-scores or DSS3 z-scores. The drugs with the strongest coefficients in both models are labeled. ***C,*** Association between mean survival coefficients and mean test C-index change (50 tests) in response to drug withdrawal for different models. The horizontal and vertical bars indicate the standard deviations, respectively. Standard or alternate AML treatment drugs are color-coded in *A-C*. The outliers are the same as marked in figure B. ***D,*** C-index results (200 tests) for Cox models trained on different drug sensitivity z-scores for treatment drugs. The horizontal lines indicate the respective C-index test medians for the full models in *Fig. 1E*. ***E,*** Clustering of normalized Ridge coefficients from models against different clinical outcomes using different drug sensitivity z-scores. ***F,*** Clustering of drug target enrichment p-values based on directional drug-set enrichment on ranked coefficients from Ridge models against different clinical outcomes using DSS3 z-scores. ***G,*** Clustering of drug class enrichment p-values based on directional drug-set enrichment on ranked coefficients from Ridge models against different clinical outcomes using rAUC-$\log_2$ z-scores. ***H,*** Clustering of drug class enrichment p-values based on directional drug-set enrichment on ranked coefficients from Ridge models against different clinical outcomes using DSS3 z-scores. ***I,*** Drug class association with differential sensitivity, using directional drug-set enrichment on differential drug sensitivities from *Fig. 5G*.

Table S1, Patient characteristics, related to STAR Methods.

ID	Age	Sex	Leukemia status	FAB class	Karyotype	ELN2022
4	40-49	Female	Primary	M1	Normal	Intermediate
7	50-59	Male	Primary	M2	Normal	Intermediate
9	60-69	Male	Primary	M5	Other	Intermediate
15	60-69	Male	Primary	M2		Adverse
16	60-69	Female	Secondary	Other	Normal	Intermediate
17	60-69	Male	Secondary	Other	Normal	Intermediate
18	40-49	Female	Primary	M1	Complex	Adverse
19	60-69	Female	Primary	M1	Normal	Intermediate
21	60-69	Male	Primary	M1		Intermediate
23	60-69	Female	Primary	M4	Normal	Intermediate
24	<40	Female	Primary	M1	Other	Intermediate
25	>70	Male	Unknown	M0		Favorable
27	60-69	Female	Secondary	M4/M5	Normal	Intermediate
28	50-59	Male	Primary	M4/M5	Normal	Intermediate
29	50-59	Female	Primary	M4 eos	Other	Favorable
30	40-49	Female	Primary	M4	Normal	Intermediate
31	50-59	Female	Primary	M2	Normal	Intermediate
32	60-69	Male	Primary	M1	Normal	Intermediate
33	60-69	Female	Primary	M2	Complex	Adverse
34	60-69	Male	Primary	M1	Normal	Intermediate
35	60-69	Female	Secondary		Complex	Adverse
36	<40	Female	Primary	M5	Normal	Intermediate
37	50-59	Male	Primary	M0	Other	Adverse
38	60-69	Male	Primary	M0	Normal	Intermediate
40	60-69	Male	Primary	M5	Other	Intermediate
42	60-69	Female	Primary	M2	Other	Adverse
43	60-69	Male	Secondary	M2	Normal	Intermediate
44	60-69	Male	Primary	M6	Normal	Favorable
47	40-49	Male	Primary	M5	Other	Adverse
51	40-49	Female	Primary	M4		Favorable
52	<40	Female	Primary	M1		Adverse
54	60-69	Female	Secondary	M4	Normal	Favorable
57	50-59	Female	Secondary	M5	Normal	Favorable
58	50-59	Female	Primary	M4	Normal	Intermediate
60	60-69	Female	Primary	M3	Other	Favorable
62	60-69	Female	Secondary	M5	Normal	Intermediate
64	50-59	Female	Primary	M5	Other	Intermediate
65	60-69	Male	Primary	M5	Normal	Adverse
66	60-69	Male	Primary	Other	Other	Adverse
70	50-59	Female	Primary	M3	Normal	Favorable

71	40-49	Female	Primary	M5	Other	Intermediate
73	<40	Female	Primary	M2	Other	Adverse
74	50-59	Male	Primary	M4	Other	Adverse
75	40-49	Female	Primary	M1	Normal	Intermediate
76	50-59	Male	Primary	M1	Normal	Intermediate
78	60-69	Male	Secondary	M4	Normal	Intermediate
79	50-59	Male	Primary	M7	Other	Favorable
80	<40	Male	Primary	M6	Normal	Favorable
82	60-69	Male	Primary	Other	Complex	Adverse
83	>70	Female	Unknown		Normal	Intermediate
84	60-69	Female	Primary	M2	Other	Favorable
85	40-49	Female	Primary	M0	Other	Adverse
88	>70	Female	Primary	M4	Normal	Intermediate
89	50-59	Female	Primary	M2	Normal	Favorable
92	<40	Female	Primary	M1	Normal	Intermediate
93	<40	Male	Primary	M2	Normal	Favorable
95	50-59	Female	Primary	M4/M5	Normal	Favorable
98	50-59	Male	Secondary	Other	Normal	Intermediate
100	50-59	Male	Primary	M4 eos	Other	Intermediate
105	60-69	Male	Primary	M1/M2	Complex	Adverse
106	>70	Female	Primary	M1/M2	Normal	Intermediate
107	>70	Male	Primary	M4/M5	Other	Favorable
108	50-59	Male	Primary	M3	Normal	Favorable
111	60-69	Female	Primary		Normal	Intermediate
114	40-49	Female	Primary	M1/M2	Other	Adverse
115	60-69	Male	Primary	M1/M2	Complex	Adverse
116	60-69	Male	Primary	M1/M2	Normal	Intermediate
117	>70	Male	Primary		Other	Adverse
130	>70	Female	Secondary	Other	Other	Adverse

Table S2, Overview of treatment and disease course, related to STAR Methods.

ID	Induction	HMAS	Allogenic BM transplant	Persistent leukemia post first induction treatment	Relapse	Treatment Naive	Cures given
100	Standard therapy	Yes	No	No	No	Yes	1-2
105	Standard therapy	No	Yes	No	No	Yes	1-2
106	Standard therapy	No	No	Yes	Yes	Yes	3-5
107	Standard therapy	No	No	No	No	Yes	1-2
108	Other	No	No	No	No	Yes	1-2
111	Standard therapy	Yes	No	No	Yes	Yes	1-2
114	Standard therapy	No	Yes	Yes	Yes	Yes	1-2
115	Standard therapy	No	Yes	Yes	Yes	Yes	3-5
116	Standard therapy	No	Yes	Yes	Yes	Yes	3-5
117	Standard therapy	No	No	Yes	Yes	Yes	3-5
130	Other	No	No	Yes	Yes	No	3-5
15	Standard therapy + Lenalidomide	No	Yes	No	No	Yes	1-2
16	Other	No	No	No	Yes	Yes	1-2
17	Standard therapy	No	Yes	No	Yes		1-2
18	Standard therapy	No	Yes	No	No	Yes	1-2
19	Standard therapy	No	Yes	No	Yes	Yes	3-5
21	Standard therapy	No	No	No	Yes	Yes	1-2
23	Standard therapy + Lenalidomide	No	No	No	No	Yes	1-2
24	Standard therapy + Lenalidomide	No	Yes	No	No	Yes	1-2
25	Standard therapy	No	No	Yes	No	Yes	1-2
27	Standard therapy	No	Yes	Yes	Yes		3-5
28	Standard therapy	No	Yes	No	Yes	No	3-5
29	Standard therapy + Lenalidomide	No	No	No	No	Yes	1-2
30	Standard therapy	No	No	No	Yes	Yes	3-5
31	Standard therapy	No	Yes	No		Yes	3-5
32	Standard therapy	No	Yes	Yes	No	Yes	3-5
33	Standard therapy	No	No	No	No	Yes	1-2
34	Standard therapy	No	Yes	No	Yes	Yes	3-5
35	Other	No	Yes	Yes	Yes	Yes	3-5
36	Standard therapy	No	Yes	Yes	Yes	No	3-5
37	Standard therapy	No	No	No	No	Yes	1-2
38	Standard therapy + Lenalidomide	No	Yes	No	Yes	Yes	1-2

4	Standard therapy	Yes	No	No	No	Yes	3-5
40	Standard therapy	No	Yes	No	No	Yes	1-2
42	Standard therapy	No	No	Yes	Yes	Yes	3-5
43	Standard therapy	No	Yes		Yes	No	3-5
44	Standard therapy + Lenalidomide	Yes	No	No	No	Yes	1-2
47	Standard therapy	No	Yes	No	Yes	No	Unknown
51	Standard therapy + Lenalidomide	Yes	Yes	No	Yes	Yes	3-5
52	Standard therapy	No	No	Yes	Yes	Yes	3-5
54	Standard therapy	No	No	No	No	Yes	1-2
57	Standard therapy + Lenalidomide	Yes	No	No	Yes	No	1-2
58	Standard therapy + Lenalidomide	Yes	Yes	No	Yes	Yes	3-5
60	Other	No	No	No	No	Yes	1-2
62	Standard therapy	No	No	No	No	Yes	1-2
64	Standard therapy + Lenalidomide	No	Yes	Yes	No	Yes	1-2
65	Standard therapy	No	Yes	No	No	Yes	1-2
66	Standard therapy	No	Yes	No	No	Yes	1-2
7	Standard therapy	No	Yes		Yes	No	Unknown
70	Standard therapy	No	No	Yes	No	Yes	1-2
71	Standard therapy	Yes	No	No	Yes	Yes	3-5
73	Standard therapy	No	Yes	No	No	Yes	1-2
74	Standard therapy + Lenalidomide	No	Yes	No	Yes	Yes	3-5
75	Standard therapy	Yes	Yes	No	Yes	Yes	3-5
76	Standard therapy	No	Yes	No	Yes	Yes	3-5
78	Standard therapy	No	Yes	No	No	Yes	1-2
79	Standard therapy	Yes	No	No	No	Yes	1-2
80	Standard therapy	Yes	No	No	No	Yes	1-2
82	Other		Yes				1-2
83	Unknown		No				Unknown
84	Standard therapy + Lenalidomide	No	No	No	No	Yes	1-2
85	Standard therapy	No	Yes	No	No	Yes	3-5
88	Standard therapy	No	No	No	Yes	Yes	3-5
89	Standard therapy	Yes	No	No	No	Yes	1-2
9	Standard therapy + Lenalidomide	Yes	No	No	No	Yes	3-5
92	Standard therapy	Yes	No	No	No	Yes	1-2

93	Standard therapy + Lenalidomide	No	Yes	No	Yes	Yes	1-2
95	Standard therapy	Yes	No	No	No	Yes	1-2
98	Other	No	No	Yes	Yes	Yes	1-2

Table S3, Sample and batch information, related to STAR Methods.

ID	Sample ID	Source	Instrument	Instrument serial number	Cells_per_well
100	Patient 100	BM	VictorX	20302104	10000
105	Patient 105	Blood	VictorX	20302104	10000
106	Patient 106	BM	VictorX	20302104	10000
107	Patient 107	Blood	VictorX	20302104	10000
108	Patient 108	BM	VictorX	20302104	10000
111	Patient 111	BM	VictorX	20302104	10000
114	Patient 114	BM	VictorX	20302104	10000
115	Patient 115	BM	VictorX	20302104	406
115	Patient 115_relapse	BM	EnVision	1020030	
116	Patient 116	BM	VictorX	20302104	10000
117	Patient 117	BM	VictorX	20302104	10000
130	Patient 130	BM	VictorX	20302104	2000
15	Patient 15	BM	EnVision	1040142	2200
16	Patient 16	Blood	EnVision	1040142	10000
17	Patient 17	Blood	EnVision	1040142	10000
18	Patient 18	BM	EnVision	1040142	10000
19	Patient 19	BM	EnVision	1040142	10000
21	Patient 21	Blood	EnVision	1040142	10000
23	Patient 23	BM	EnVision	1040142	10000
24	Patient 24	BM	EnVision	1040142	10000
25	Patient 25	BM	EnVision	1040142	10000
27	patient 27	BM	EnVision	1040142	10000
28	patient 28	BM	EnVision	1040142	3676
29	patient 29	BM	EnVision	1040142	8000
30	Patient 30	Blood	EnVision	1040142	10000
30	Patient 30_relapse	Blood	EnVision	1040142	10000
31	Patient 31	Blood	EnVision	1040142	10000
32	Patient 32	BM	EnVision	1040142	10000
33	Patient 33	BM	EnVision	1040142	10000
33	Patient 33_relapse	BM	EnVision	1040142	5000
34	Patient 34	BM	EnVision	1040142	10000
34	Patient 34_relapse	BM	VictorX	20302104	
35	Patient 35	Blood	EnVision	1040142	2823
36	Patient 36	BM	EnVision	1040142	10000
37	Patient 37	BM	EnVision	1040142	2554
38	Patient 38	BM	EnVision	1040142	2382
4	Patient 4	BM	EnVision	1040142	8500
40	Patient 40	Blood	EnVision	1040142	10000
42	Patient 42	BM	EnVision	1040142	10000
42	Patient 42_relapse	BM	EnVision	1040142	10000
43	Patient 43	BM	EnVision	1040142	661
44	Patient 44	BM	EnVision	1040142	10000

47	Patient 47	BM	EnVision	1040142	4200
51	Patient 51	BM	EnVision	1040142	10000
52	Patient 52	BM	EnVision	1040142	10000
54	Patient 54	BM	EnVision	1040142	10000
57	Patient 57	Blood	EnVision	1040142	10000
58	Patient 58	BM	EnVision	1040142	10000
60	Patient 60	BM	EnVision	1040142	1352
62	Patient 62	Blood	EnVision	1040142	10000
64	Patient 64	BM	EnVision	1040142	10000
65	Patient 65	BM	EnVision	1040142	5000
66	Patient 66	BM	EnVision	1040142	1000
7	Patient 7	BM	EnVision	1040142	10000
70	Patient 70	Blood	EnVision	1040142	10000
71	Patient 71	BM	EnVision	1040142	10000
71	Patient 71_relapse	BM	VictorX	20302104	
73	Patient 73	BM	EnVision	1040142	750
74	Patient 74	BM	EnVision	1040142	10000
75	Patient 75	BM	EnVision	1040142	795
76	Patient 76	BM	EnVision	1040142	1205
76	Patient 76_relapse	BM	VictorX	20302104	
78	Patient 78	BM	EnVision	1040142	10000
79	Patient 79	BM	EnVision	1040142	10000
80	Patient 80	BM	VictorX	20302104	1000
82	Patient 82	BM	VictorX	20302104	1500
83	Patient 83	BM	VictorX	20302104	3911
84	Patient 84	BM	VictorX	20302104	2000
85	Patient 85	BM	VictorX	20302104	533
88	Patient 88	BM	VictorX	20302104	10000
88	Patient 88_relapse	BM	VictorX	20302104	
89	Patient 89	BM	VictorX	20302104	10000
9	Patient 9	BM	EnVision	1020030	10000
92	Patient 92	Blood	VictorX	20302104	10000
93	Patient 93	BM	VictorX	20302104	10000
95	Patient 95	Blood	VictorX	20302104	10000
98	Patient 98	BM	VictorX	20302104	10000
